# A Multifunctional Hydrogel with Multimodal Self-Powered Sensing Capability and Stable Direct Current Output for Outdoor Plant Monitoring Systems

**DOI:** 10.1007/s40820-024-01587-y

**Published:** 2024-11-27

**Authors:** Xinge Guo, Luwei Wang, Zhenyang Jin, Chengkuo Lee

**Affiliations:** 1 Department of Electrical & Computer Engineering, National University of Singapore, 4 Engineering Drive 3, Singapore, 117576 Singapore; 2 Center for Intelligent Sensors and MEMS (CISM), National University of Singapore, 5 Engineering Drive 1, Singapore, 117608 Singapore; 3 NUS Graduate School - Integrative Sciences and Engineering Program (ISEP), National University of Singapore, Singapore, 119077 Singapore; 4 Research Center for Sustainable Urban Farming (SUrF), National University of Singapore, Singapore, 117558 Singapore

**Keywords:** Self-powered sensor, Hydrogel, Energy harvester, Outdoor farming, Self-sustainable IoT

## Abstract

Smart farming with outdoor monitoring systems is critical to address food shortages and sustainability challenges. These systems facilitate informed decisions that enhance efficiency in broader environmental management. Existing outdoor systems equipped with energy harvesters and self-powered sensors often struggle with fluctuating energy sources, low durability under harsh conditions, non-transparent or non-biocompatible materials, and complex structures. Herein, a multifunctional hydrogel is developed, which can fulfill all the above requirements and build self-sustainable outdoor monitoring systems solely by it. It can serve as a stable energy harvester that continuously generates direct current output with an average power density of 1.9 W m^−3^ for nearly 60 days of operation in normal environments (24 °C, 60% RH), with an energy density of around 1.36 × 10^7^ J m^−3^. It also shows good self-recoverability in severe environments (45 °C, 30% RH) in nearly 40 days of continuous operation. Moreover, this hydrogel enables noninvasive and self-powered monitoring of leaf relative water content, providing critical data on evaluating plant health, previously obtainable only through invasive or high-power consumption methods. Its potential extends to acting as other self-powered environmental sensors. This multifunctional hydrogel enables self-sustainable outdoor systems with scalable and low-cost production, paving the way for future agriculture.

**Supplementary Information:**

The online version contains supplementary material available at 10.1007/s40820-024-01587-y.

## Highlights

- A simple and scalable fabricated hydrogel with multiple functionalities to realize self-sustainable outdoor monitoring solely by it for large-scale applications in precision agriculture.
- Stable direct-current output without requirement on stochastic or temporal environmental energies, achieving an energy density of 1.36 × 10^7^ J m^–3^ with continuous operation of 56.25 days in normal outdoor environment.
- Self-powered noninvasive leaf relative water content monitoring and environmental sensing to evaluate plant health status with high durability and self-recoverability in severe environments.

**Supplementary Information:**

The online version contains supplementary material available at 10.1007/s40820-024-01587-y.

## Graphical abstract

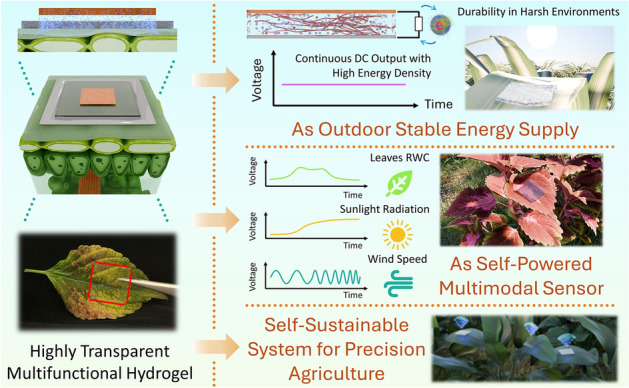

## Introduction

Climate change is dramatically impacting agricultural productivity. Global warming, unpredictable rainfall, and severe weather conditions threaten crop yields and cropping frequency when food demand rises due to changing diets and a growing global population, projected to reach 9.7 billion by 2050 [1–3]. In response, smart farming is emerging as a crucial innovation, offering sophisticated data management systems that deliver precise information directly to agricultural decision-makers [4, 5]. While smart sensing technologies have already proven effective in indoor agriculture, particularly in monitoring environmental parameters and plant health, their expansion into outdoor settings is still in its formative stages. This development is driven by the demand for cost-effective agriculture technology for the vast expanses of unexploited farmland and the significant potential for improvements in yield and for improvements in productivity and sustainability of outdoor farming [6, 7]. Further enhanced by smart sensing technology, the sophisticated practices create an imperative transition from current farming technology to future sensing-enhanced technology such that food security and self-sustainable green earth will be achieved [8–12].

The Internet of Things (IoT) is pivotal in modern precise agriculture, merging physical and digital realms to enhance production efficiency [13]. Through cloud-assisted wireless sensor networks [14–16], IoT enables real-time remote monitoring, providing critical information on environmental conditions, crop status, and autonomous agricultural machinery [17, 18]. However, these systems encounter challenges for outdoor applications. IoT struggles to adapt to dynamic, unpredictable outdoor conditions without human intervention, suffering performance setbacks from environmental disruptions like soil erosion and rainstorms [19]. Moreover, the maintenance demands, such as repairs, reconfigurations, and, most importantly, battery replacements, of traditional IoT systems are complicated and even become a concern due to the widespread distribution of IoT nodes over large remote areas [20–22].

Wearable electronics and sensors have attracted increasing research interest owing to their promising applications in real-time and precise monitoring and tracking of critical information to help provide timely suggestions and avoid potential dangers [23–30]. Self-powered sensors are favored in the development of wearable technologies for their autonomous operation without the need for an external power supply, which may further reduce overall power consumption and the frequency of maintenance interventions of IoT sensing systems [31–39]. These self-powered sensors typically employ triboelectric, piezoelectric, pyroelectric principles, etc., to detect external physical properties such as motion, force, strain, and temperature [40–49]. However, monitoring internal characteristics of the human body or plants, which are equally important, often requires specific high-power devices, which significantly limits their widespread application, especially in outdoor environments.

Energy harvesting emerges as a promising eco-friendly strategy in outdoor IoT smart farming that enhances the operation longevity of systems by minimizing battery depletion [50] and ultimately realizing IoT-system-level self-sustainability [51–57]. In outdoor environments, various energy scavenging principles have been developed to capture solar energy from sun radiation, mechanical energy from wind and vibration, and thermal energy from temperature gradients [58–60]. Triboelectric nanogenerators (TENGs), in particular, have shown superiority due to their simple fabrication, lightweight, versatility, and availability of diverse material choices, including flexible and biocompatible materials suitable for smart farming applications [61–63]. However, maintaining average power density over extended periods, such as 1–2 months, remains challenging for these energy harvesters (EHs). Besides, TENGs are highly dependent on intermittent high-frequency vibrational energy present in the environment and are susceptible to humidity and mechanical wear due to their friction and contact-based charge generation [64–66]. Additional designs in TENG structures or circuits are also required to generate a direct current (DC) output [67].

Similarly, electromagnetic and piezoelectric-based EHs require excitation attributed to vibration sources with continuous or intermittent vibrations available in the environment, where such vibration could not always exist, and the vibration frequency and amplitude are irregular [68–70]. Furthermore, complex micro-electro-mechanical system designs are prone to damage in harsh outdoor environments. On the other hand, the lead-content piezoelectric EHs, e.g., PZT, face regulation limitations because of using lead, which shows concern in terms of environmentally harmful materials [71–73]. Recently proposed hybrid energy harvesters attempt to address the instability and episodic nature of required energy in the environment by integrating multiple energy conversion principles [74, 75]. However, their complexity and impedance matching issues among different principles still lead to low durability and low efficiency when hybrid EHs are used in the outdoor environment. To replace the battery for the operation of IoT sensing nodes, hybrid EHs with steady DC output in diverse outdoor conditions without reliance on intermittent and episodic energy sources remain as technical challenges [76, 77].

On the other hand, plant wearable sensors for site-specific monitoring with high spatiotemporal resolution epitomize a leap in precision agriculture [78–81]. Because of proximity to individual plants, such sensors enable more intuitive multifactorial monitoring of intricate plant physiology and localized microclimate, especially regarding the detection of nuanced shifts in plant water status critical for photosynthesis and transpiration [78, 82–84]. At the same time, self-powered sensors also boost energy sustainability in smart farming [85–87]. However, the integration of self-powered features with wearable plant sensors remains challenged, for the commonly applied piezoelectric, triboelectric, and pyroelectric principles are hard to measure vital biological signals for evaluating plant health status, such as the photosynthetic rate, chlorophyll content, or relative water content (RWC) [88, 89]. Furthermore, these previously widely applied principles are also difficult to build up a self-sustainable outdoor monitoring system in which the self-powered sensors and EHs are fully based on the same device, leading to structure, fabrication, and circuit complexity. These materials also typically lack transparency or a soft Young’s modulus matching that of plants. Recently, hydrogels as organic materials with low cost, good flexibility, biodegradability, and biocompatibility have attracted growing attention [90–94] and have been applied in plant monitoring systems [95–97]. However, most previous studies focus on using it as resistive or capacitive-based plant sensors or only as electrodes or interfaces for self-powered plant sensors [98–100]. Their capabilities to perform simultaneously as EHs and self-powered sensors in outdoor smart farming, with the potential to monitor vital plant biological signals, have often been overlooked.

**Fig. 1:**
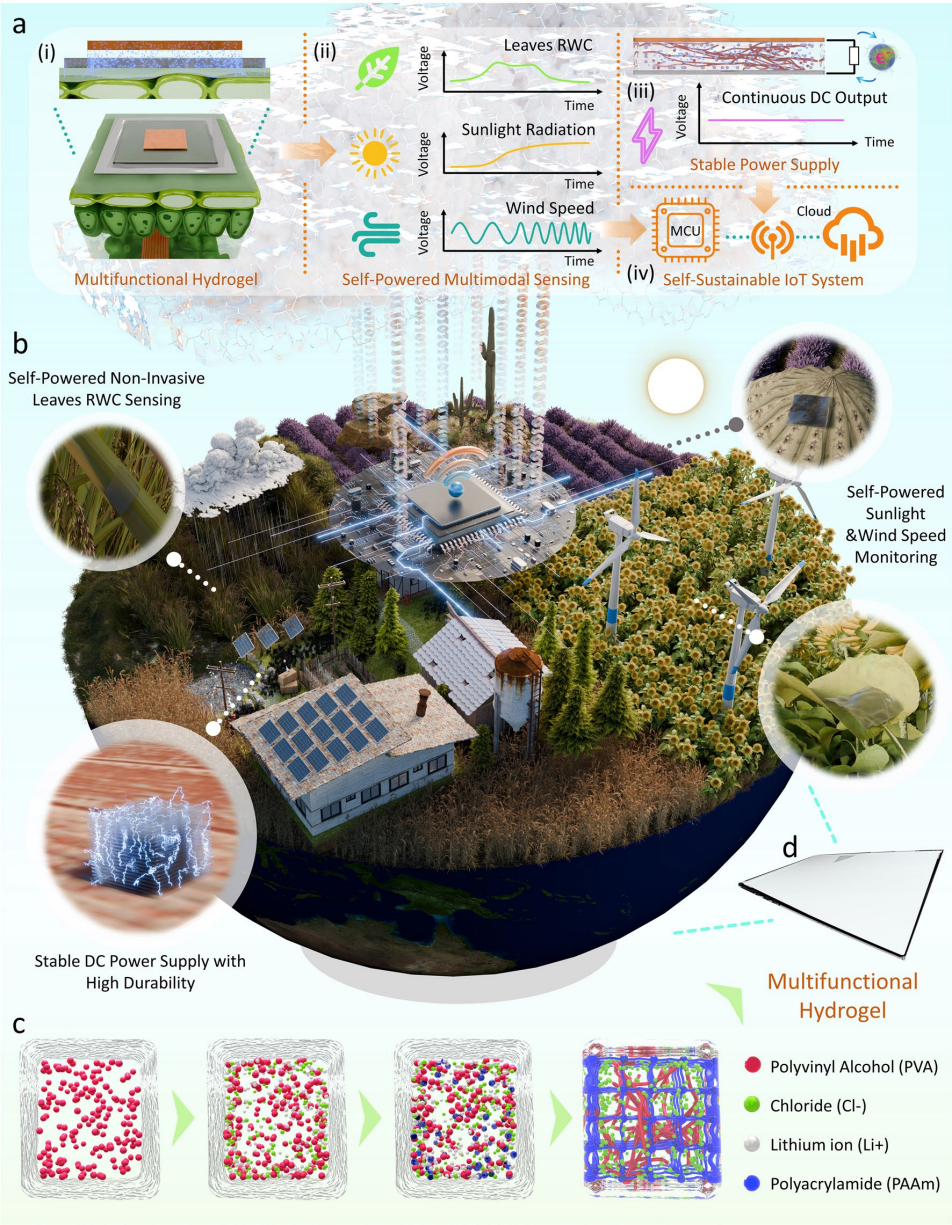
Self-sustainable outdoor plant monitoring system for future smart agriculture empowered by the multifunctional hydrogel. **a** Structure of the proposed self-sustainable IoT system: **(i)** the ionic hydrogels can be fabricated scalable and attached to plant leaves, **(ii)** the multifunctional hydrogels can realize multimodal sensing including noninvasive plant RWC sensing to monitor plant health and environmental sensing of sunlight radiation monitoring and wind speed, **(iii)** the ionic hydrogel can also serve as energy harvester to provide continuous DC output to power the whole system; **(iv)** the sensing signals can be read by designed low-power MCU and sent out wirelessly to cloud for agricultural decision-makers. **b** Illustration of the proposed self-sustainable outdoor monitoring system for future outdoor smart agriculture in different environments. **c** Illustration of the fabrication process of the multifunctional hydrogel (details provided in the Experimental Section) and **d** the illustration of hydrogel after molding

Herein, a multifunctional hydrogel is proposed, as shown in Fig. 1a(i). It is mainly comprised of a polyvinyl alcohol (PVA)/polyacrylamide (PAAm)/lithium chloride (LiCl)/glycerol (Gly) ionic hydrogel and Cu-Al metal pairs as the electrode layers, with the influence from different metals and hydrogel thickness, and corresponding electrochemical model being fully studied. The fabricated hydrogel has good transparency and flexibility, as shown in Fig. S1. This multifunctional hydrogel can not only act as a self-powered sensor to monitor environmental signals such as sunlight and wind speed but also can firstly achieve the noninvasive monitoring of leaf RWC to determine the plant health status (Fig. 1a(ii)). Furthermore, it can also serve as an energy harvester that generates a stable DC output to power the IoT systems (Fig. 1a(iii)). It has an average power density of 1.9 W m^−3^ with good durability, proved by such value can be maintained even after 60 days of continuous powering in normal environments (24 °C, 60% RH). During this process, the whole energy generated has a density of more than 1.36 × 10^7^ J m^−3^, proving its high efficiency in powering outdoor IoT systems. It also possesses good resilience to severe conditions (45 °C, 30% RH) in outdoor environments, with the ability to self-recover to normal power density on rainy days (24 °C, 99% RH). Such fatigue-recover cycles have been tested 13 times in 40 days, with its output power density still not being compromised. Lastly, a self-sustainable IoT system has been built (Fig. 1a(iv)) to demonstrate the capability and applications of such multifunctional hydrogel in the future outdoor plant monitoring realm for smart farming (Fig. 1b). The ionic hydrogel possesses a simple and scalable one-pot fabrication process, as depicted in Fig. 1c, making it easily applied on a large scale in outdoor agriculture. The proposed multifunctional hydrogel (Fig. 1d), for its self-powered multimodal sensing capability, noninvasive plant health monitoring ability, high durability and resilience as a stable energy source to power the IoT systems in unpredictable outdoor environments, and scalability and low cost in fabrication, may become the ideal choice for achieving the future self-sustainable earth.

## Experimental Section

### Preparation of PVA/PAAm/LiCl/Gly Ionic Hydrogels as Energy Harvesters and Self-Powered Sensors

The synthesis of PVA/PAAm/LiCl/Gly ionic hydrogels is a straightforward and facile process involving several steps. The initial step is dissolving 3.7532 g of poly(vinyl alcohol) polymers (Mw 89,000–98,000, 99 + % hydrolyzed, 341,584, Sigma-Aldrich), in 22.5 mL of deionized (DI) water. PVA serves as the polymer matrix of synthetic hydrogels. Next, 2.0268 g nontoxic glycerin (Gly, for molecular biology, ≥ 99.0%, G5516, Sigma-Aldrich), and 0.09763 g of LiCl powder (ACS reagent, ≥ 99%, 310,468, Sigma-Aldrich) are added to the aqueous solution slowly for the desired Gly concentration of 36% in the ionic electrolyte. Gly has dual functionality as a humectant and plasticizer in the ionic composite, allowing for modulation of water content and electrical impedance, as well as an increase in the flexibility of materials. As for the LiCl powder, it plays the role of ion source for activation and intensification of the charge flowing in our hydrogels, which serves as an electrolyte. The resulting mixture of solution is then placed in a thermostat water bath at 600 rpm stirring and 90 °C condition, for 90 min until all the ingredients completely dissolve in water. The solution is then taken out to be cooled down to room temperature. Following this, 1.8766 g of acrylamide monomers (AAm, for molecular biology, ≥ 99% (HPLC), A9099, Sigma-Aldrich), and 2.5 mg of N, N’-Methylenebis-acrylamide (MBA, powder, for molecular biology, suitable for electrophoresis, ≥ 99.5%, M7279, Sigma-Aldrich), and 7.5 mg of ammonium persulfate (APS, for molecular biology, suitable for electrophoresis, ≥ 98%, A3678, Sigma-Aldrich) are added to the PVA/LiCl/Gly solution as crosslinker and initiator, respectively. The obtained solution is then placed back into the thermostat water bath at 600 rpm stirring and 45 °C condition for another 30 min to completely dissolve. The precursor solution is then poured into customized 3D-printed molds made of PLA (PolyLite, Polymaker) and placed in a vacuum chamber to remove the tiny air bubbles inside the electrolyte solution. The molds used for fabricating the hydrogel are manufactured through 3D printing using fused deposition modeling (FDM) technology. The printing material is PLA (PolyLite™ PLA, Polymaker, 1.75 mm diameter), and the process is carried out with a [Raise3D Pro3] 3D printer. This printer offers a maximum resolution of 0.4 mm in the x and y directions and 0.1 mm in the z direction, ensuring precise and high-quality molds for hydrogel fabrication. Subsequently, the solution is dried in a dry cabinet at 20% RH and an ambient temperature of 24 °C for 24 h. It is then dried in an environmental chamber at 60% RH and 24 °C for an additional 48 h. After drying, the PVA/PAAm/LiCl/Gly ionic hydrogels (~ 300 μm thickness) are peeled off from the molds. By designing different 3D-printed molds, the size, thickness, and patterns of the fabricated hydrogels can be easily altered, enabling scalable fabrication. Therefore, the thickness of the hydrogels does not effect the scalability of fabrication. In addition, the mechanical properties and flexibility of hydrogels with thickness ranging from 300 to 750 μm are provided in Figs. S2 and S3, respectively.

The proposed hydrogel-based energy harvester is also fabricated through a scalable, simple, and low-cost process. The copper and aluminum electrodes are cut to a 20 × 20 mm^2^ square shape from commercial copper foil (Copper Foil Tape 1181, 3 M) and aluminum foil (Aluminum Foil Tape 1436, 3 M). The thickness of the copper layer and the aluminum layer is 66 and 75 μm, respectively. As previously discussed, the PVA/PAAm/LiCl/Gly ionic hydrogels have been pre-formed to a 30 × 30 mm^2^ size using a customized 3D-printed mold. The fabrication begins by attaching the copper and aluminum electrode films to two sides of the ionic hydrogel, respectively. The bottom aluminum electrode layer and hydrogel are adhered together to a substrate by the double-sided tape (3MTM VHBTM Tape, 35 × 35 × 3 mm^3^). Subsequently, multiple pre-made power sources can be cascaded in a manner that the cathode electrode layer (Aluminum Foil Tape 1436, 3 M) extending from the front piece of the hydrogel is in contact with the anode electrode (Copper Foil Tape 1181, 3 M) extending from the back piece, and the contact part of the two electrodes is fixed on the base using scotch tape. In this way, the power source can be connected repeatedly, and the output potential can be increased by such cascading connections.

As self-powered leaf RWC sensors, a 10 × 10 mm^2^ iece of copper foil (Copper Foil Tape 1181, 3 M) serving as the cathode is initially affixed to a 40 × 40 mm^2^ square-shaped piece of Scotch tape. Subsequently, the PVA/PAAm/LiCl/Gly ionic hydrogel is fabricated and molded into a 20 × 20 mm^2^ square shape, which is then adhered to the same Scotch tape atop the copper foil. Following this, a 30 × 30 mm^2^ piece of aluminum foil (Aluminum Foil Tape 1436, 3 M) is cut out, and a 20 × 20 mm^2^ square is symmetrically hollowed out from its center. This aluminum foil piece is also attached to the Scotch tape, positioned at the edge of the hydrogel, effectively enclosing it. The entire sensor is then inverted onto a leaf and securely affixed to measure RWC using the viscosity of the Scotch tape.

The hydrogel-based self-powered wind speed sensor was prepared by first adhering a 10 × 10 mm^2^ piece of copper foil (Copper Foil Tape 1181, 3 M) to the surface of a leaf using double-sided tape. A 20 × 20 mm^2^ piece of PVA/PAAm/LiCl/Gly ionic hydrogel is then attached to the leaf over the copper foil and adhered by the double-sided tape similarly. Subsequently, a 10 × 10 mm^2^ piece of aluminum foil (Aluminum Foil Tape 1436, 3 M) is cut out with a 5 × 50 mm^2^ tail-like extension, which is affixed on the stem of the leaf by the adhesion of the other non-conductive part side of the aluminum electrode itself. This setup allows the 10 × 10 mm^2^ section of the aluminum electrode to remain movable. As the leaf sways in the wind (with corresponding wind strength measured by an anemograph), the aluminum electrode intermittently contacts and separates with the hydrogel surface, generating a contact-separation process along with the voltage spike.

The hydrogel-based self-powered sunlight radiation sensor was fabricated and fixed on a 3D-printed PLA (PolyLite™ PLA, Polymaker, 1.75 mm diameter) substrate (50 × 50 × 3 mm^3^). The sensor comprises two electrodes: a 5 × 30 mm^2^ aluminum foil (Aluminum Foil Tape 1436, 3 M) and a 5 × 30 mm^2^ copper foil (Copper Foil Tape 1181, 3 M). These foils are attached to the substrate using a 30 × 30 mm^2^ piece of double-sided tape. The next step involves applying a PVA/PAAm/LiCl/Gly ionic hydrogel, molded into a 20 × 20 mm^2^ square shape, onto the substrate and covering upon the two electrodes. The upper surface of the hydrogel is completely colored black using a marker to enhance light radiation absorption.

### Electrochemical Impedance Spectroscopy (EIS) and Potential Measurement of the Multifunctional Hydrogel Equipped with Different Pairwise Combinations of Metallic Electrodes

The EIS measurement experiment is conducted to analyze the detailed electrochemical characteristics of the multifunctional hydrogel. The hydrogel uses varying combinations of metallic materials as electrodes. Six different kinds of metallic tapes, including Cu, Al, Mo, Ni, Ag, and Zn, are combined in pairs to form the two electrodes of the hydrogel, which are attached to both sides of a hydrogel with a thickness of 300 μm serving as the electrolyte, respectively. An electrochemical workstation (CHI760E) is configured to apply sinusoidal AC signals with an amplitude of 0.7 V across a frequency range from 10 to 0.464 MHz, and the resulting feedback data of the device’s impedance versus frequency could be output to and displayed on the computer for further processing. Similarly, for the potential measurement experiment, a 300 μm hydrogel is also equipped with different metallic electrodes combined in pairs to form the power source. Subsequently, the voltage across the load resistance is read and recorded from the very beginning of the establishment of the circuit loop by the Electrometer (Keithley, Model 6514 Electrometer).

### Characterization of the Multifunctional Hydrogel as the Energy Harvester

For average power density and the matching resistance measurement of the multifunctional hydrogel with different thicknesses, we prefabricate molds of different depths, each measuring 20 mm by 20 mm, based on a 3D printing method. Based on the previously mentioned hydrogel preparation method, hydrogels with different thicknesses $T$ of 140, 250, 300, 480, and 600 μm, and an area $S$ of 20 mm by 20 mm could be obtained. Each side of these hydrogels will be attached to an aluminum electrode on one side and a copper electrode on the other. The sustained potentials generated by the power source is directly collected across a changeable load resistance, ${R}_{L}$, which is a resistance box whose value could be subtly adjusted in order to find the matching resistance, maximum power point, and the corresponding highest average power density with different thicknesses. The voltage across the load resistance ${R}_{L}$ is read by the Electrometer (Keithley, Model 6514 Electrometer). Considering the multifunctional hydrogel directly generates stable DC output, the average power density could be calculated based on the equation: $P_{avg} = \frac{{V^{2} }}{{R_{L} TS}}$.

For the long-term durability measurements, the hydrogel is maintained in a constant environment at 60% RH and 24 °C and kept operating in the working mode by charging a supercapacitor with a capacity of 4 F. Once the voltage across the capacitor reaches the saturated potential of the hydrogel-based energy harvester, the capacitor would be discharged until the voltage across it drops to zero, and reconnected with the power source. This is to ensure the multifunctional hydrogel is always in the working mode. At each specific interval, the open-circuit potential, the matching resistance, and the corresponding maximum output power are measured using a resistance box and electrometer (Keithley, Model 6514 Electrometer). The interval is lengthened appropriately over time, from an initial 2 h to a maximum of 168 h. The total length in normal environments is 56.25 days.

As for the long-term recoverability measurements of the power source, during each measurement cycle, the hydrogel is first placed in a closed space with a temperature of 45 °C and a humidity of 30% RH for a specified period and then transferred to a sealing chamber with a temperature of 24 °C and a humidity of 99% RH for a specified period. The matching resistance and output power of the multifunctional hydrogel in such a period are measured in the same way as in normal environments. Similarly, both the heating duration and the humidification duration are extended appropriately, from initial 2/2 h to 98/24 h at last, forming a total testing length of 37.67 days and 13 fatigue-recover cycles.

### Characterization of the Multifunctional Hydrogel as Self-Powered Sensors

To calibrate the hydrogel as a self-powered leaf RWC sensor, the accurate leaf RWC value should also be measured. The first step in measuring leaf RWC is to sample the top-most fully expanded leaves unless profiling leaves on the entire plant. For smaller composite leaves like common coleus, sea hibiscus, and dry zone mahogany, combine several leaflets to form a sample. For large, broad leaves like spider lilies, cut leaf discs of about 5–10 cm^2^. Secondly, in the laboratory, the leaves are weighed to determine their fresh weight. Then, hydrate the samples to full turgidity by immersing them in tap water for 4 h at 24 °C and 60% RH. After soaking, dry the leaves gently to remove surface moisture and weigh them immediately to obtain the turgid weight (WT). At this stage, the RWC is considered 100%. Next, dry the leaves partially in an oven at 50 °C. Weigh the samples every 15 min to obtain the intermediate weight (WC) until the weight stabilizes for almost all the water content in the leaves is lost, which is the dry weight (WD). Calculate the RWC immediately at the end of every measurement session using the equation: $\text{RWC}\left(\text{\%}\right)=\frac{{W}_{C}-{W}_{D}}{{W}_{T}-{W}_{D}}\times 100$. The sensor’s output potential corresponding to different RWC levels of the leaf is then measured and recorded. The hydrogel-based sensor is attached and affixed to the leaf, whose voltage output across the cascaded load resistance (2.52 MOhm) is then measured by the Electrometer (Keithley 6514), and an oscilloscope (Agilent, InfiniiVision, DSO-X 3034A) is used to record the self-generated voltage curve.

Regarding the environment design and setup for the long-term IoT monitoring experiment, the experiment commences by attaching four multifunctional hydrogel leaf RWC sensors to the surfaces of four different leaves of a common coleus plant. An additional sensor is placed near the plant, serving as the control group, whose output will only be affected by the environment rather than the manual stimulation of the plants. A development PCB board continuously monitors all five sensors’ outputs through analog–digital conversion (ADC) functions. For the normal environments, the plant is kept in a stable environment with the aforementioned temperature and humidity settings (24 °C and 60% RH), same as the control group, for 48 h, and it is watered moderately at 9 p.m. every alternate night. This watering schedule ensures the soil remains consistently moist but not soggy, promoting healthy plant growth. For two severe environments, manual intervation is applied to simulate such severe conditions. Regarding the arid and hyperthermal environment, a halogen lamp (Philips Halogen 230 V Dimmable Aluminum Reflector, Wattage: 50 W, Luminous Flux: 400 Lumen, Cap Type: GU10, Beam Angle: 36°) is suspended approximately 40 cm above the highest plant tip for 48 h, together with normal outdoor sunlight that varies with the diurnal cycle. Regarding the pluvial and humid environment, the plant is subjected to excessive watering (500 mL every 12 h) for 48 h, intentionally abundant to induce waterlogged conditions. During this normal outdoor sunlight that varies with the diurnal cycle also exists. The data acquired by the PCB board would be further transmitted to the upper computer for postprocessing and illustration.

The preprepared hydrogel-based sunlight radiation sensor, along with its substrate, is positioned under a halogen lamp (Philips Halogen 230 V Dimmable Aluminum Reflector, Wattage: 50 W, Luminous Flux: 400 Lumen, Cap Type: GU10, Beam Angle: 36°) at a distance of 30 cm. An infrared thermometer is utilized to detect the real-time temperature increment on the surface of the sunlight radiation sensor. The sensor is first heated until its surface temperature reaches a nearly steady state, and the corresponding output potential curve is measured and recorded in the way as previously described. Then, the halogen lamp is turned off, and the sensor is allowed to cool down to room temperature. Afterward, the same operation is performed repeatedly, only each time heating the sensor to a progressively lower target temperature. Upon reaching the target temperature, the heat-up time should be recorded as quickly as possible using a second chronograph.

## Results and Discussion

### Electrodes Optimization for the Multifunctional Hydrogel with High Output Potential and Fast Build-Up Rate

**Fig. 2:**
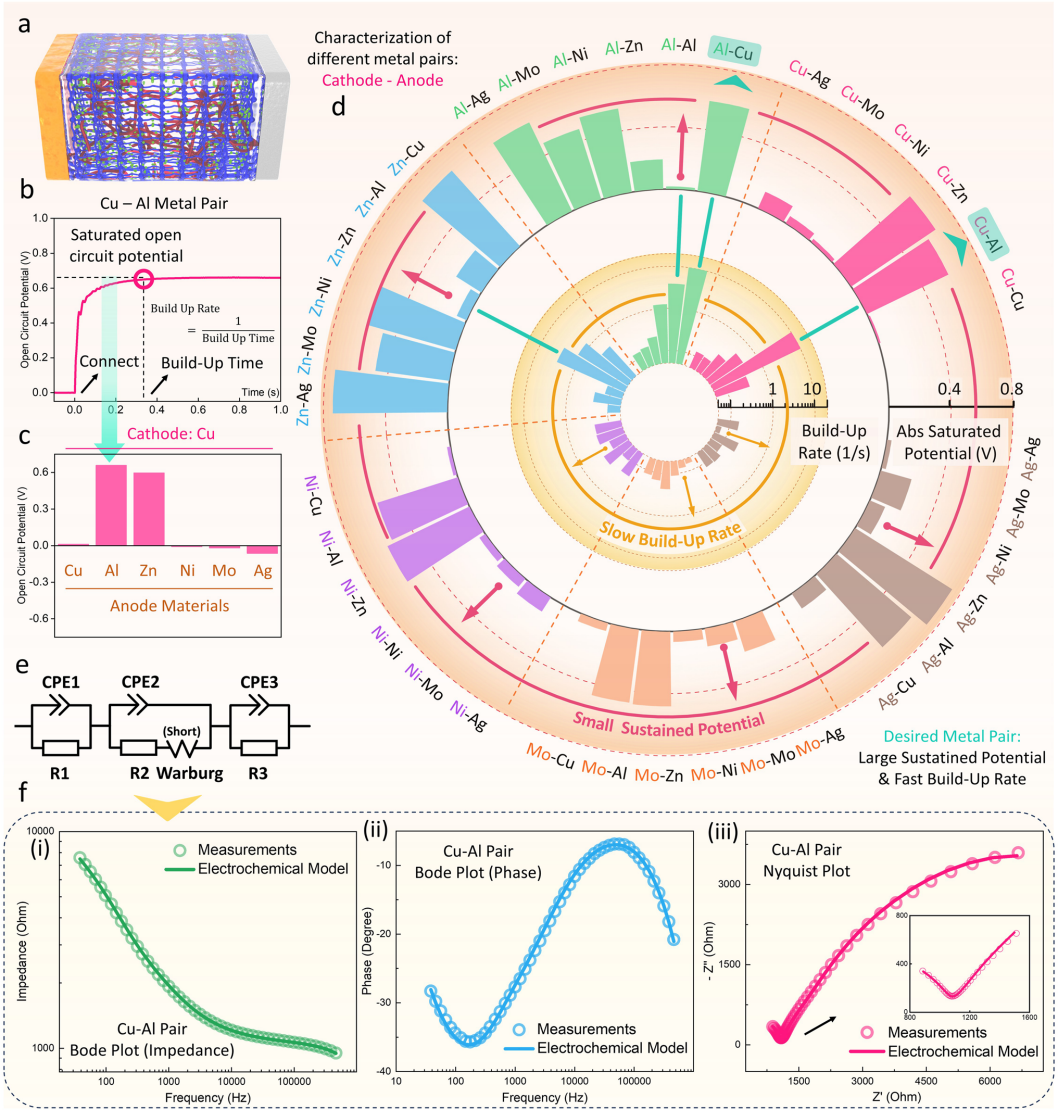
Fundamental Study and Optimization of Multifunctional Hydrogel. **a** Structure and interface illustration of the ionic hydrogel with a metal pair placed on both sides of it. **b** With Cu-Al metal pair as an example (Cu as the cathode electrode and Al as the anode electrode on both sides of PVA/PAAm/LiCl/Gly ionic hydrogel), the ionic hydrogel can build up a stable potential when connected to an external load. The build-up rate (namely the reciprocal of build-up time) and the magnitude of the potential determine the performance of the multifunctional hydrogels. **c** Comparison of the saturated potential of the multifunctional hydrogel with Cu as the cathode material and different metals as the anode materials. **d** Comparison of the absolute saturated potential output and build-up rate by employing different metal pairs for the multifunctional hydrogel. The device with Cu-Al metal pair possesses the largest saturated potential and fastest build-up rate compared to devices with other metal pairs. Detailed curves and values for the testing results can be found in Figs. S4, S5 and Table S1. **e** The corresponding electrochemical model of the device, with detailed discussion in Note S2. **f** Characteristics of the multifunctional hydrogel with Cu-Al metal pairs obtained through electrochemical impedance spectroscopy (EIS) measurements and the comparison with the proposed electrochemical model: **(i)** Bode plot of the absolute amplitude of the impedance under various frequencies; **(ii)** Bode plot of the phase under various frequencies; **(iii)** Nyquist plot of the measured results and modeling results

The characterization of the PVA/PAAm/LiCl/Gly ionic hydrogel-based device with different metal pairs is provided in Fig. 2. It is necessary to find the optimized structure to make it able to act simultaneously as a power supply and a self-powered sensor with high output, good signal-to-noise ratio, and fast response time. Different metal pairs are placed on both sides of it to stimulate the ionic hydrogel to generate a stable potential, with an illustration shown in Fig. 2a. Various ions within the hydrogel, including H^+^, Li^+^, and Cl^−^, etc., can be drawn to participate in reactions due to the different redox properties of the metal pairs on both sides, thereby establishing a stable potential difference. Such reactions vary with different applied metals and, therefore, have different net cell potentials, impedance, and response characteristics. We employ two metals among Al, Ni, Zn, Cu, Ag, and Mo, as the metal pairs (Cathode–Anode) on both sides of the hydrogel, with picking the Cu-Al metal pair as an example (Fig. 2b). It can be noticed that when the device is connected to the circuit, it requires a certain time to reach its stable potential. This build-up time varies between different metal pairs for their specific capacitance properties and exchange current densities. To establish a basic benchmark for comparison, the 95% value of its maximum stable potential is set as the saturated open circuit potential, and the time required to achieve that value is the build-up time. The reciprocal of build-up time is also calculated as the build-up rate. Figure 2c shows that when Cu is picked as the cathode, some metals, such as Ni, Mo, and Ag, show negative open circuit potential. However, in practical applications, such behaviors will not affect the usage by simply reversing the polarity of the wire connections. Therefore, only their absolute values are taken into consideration for further comparison, as shown in Fig. 2d. The detailed curves from which the values in Fig. 2d are obtained are all provided in Figs. S4, S5, and Table S1. From such results, it can be noticed that while some metal pairs possessing higher saturated potential, such as Zn-Ag, Cu–Zn, Al-Ag, etc., they are not suitable to be applied as self-powered sensors for their slower build-up rate, which results from their higher capacitive characteristics and lower exchange current density. Based on the results, the Cu-Al metal pair, which has the optimized balance between the saturated potential and the build-up rate, is picked as the metal pair for further applications of this multifunctional ionic-hydrogel-based device.

Furthermore, an electrochemical model is proposed (Fig. 2e) to study the foundamental characteristics of the PVA/PAAm/LiCl/Gly ionic hydrogel and Cu-Al metal pairs as electrodes. The two interfaces between metal electrodes and hydrogel electrolyte are described by corresponding two Randels circuits where both Faradaic currents (charge transfer resistance, R1, and R3) and non-Faradaic currents (double-layer capacitance, CPE1 (Constant Phase Element 1) and CPE3) flow through the metal-hydrogel interface. These two elements (R and CPE) are represented in parallel to reflect that the total current is the sum of both Faradic and non-Faradaic pathways. The detailed discussion of CPE in the proposed multifunctional hydrogel can be found in Fig. S11 and Note S2. The hydrogel between the metal pair is conceptualized as a thin-film electrolyte solution with increased resistance, ranging from hundreds of kOhms to several MOhms. Since there are electroactive species diffuse to the electrode’s surface, where they undergo oxidation or reduction via charge transfer, we introduce an additional Warburg short diffusion term (Ws) (details in Note S2) in series with the charge transfer resistance **R2** and the double-layer capacitance **CPE2**. The impedance of the multifunctional hydrogel can be modeled well by the following equation:

$$ Z_{all} = Z_{Rs} + \frac{1}{{\frac{1}{{Z_{CPE1} }} + \frac{1}{{Z_{R1} }}}} + \frac{1}{{\frac{1}{{Z_{CPE2} }} + \frac{1}{{Z_{WS} + Z_{R2} }}}} + \frac{1}{{\frac{1}{{Z_{CPE3} }} + \frac{1}{{Z_{R3} }}}} $$

**Rs** is the external resistance that accounts for all resistances associated with wires, clips, or other contacts. Since this value is significantly smaller compared to the resistance of the hydrogel, **Rs** is ignored and treated as 0 for the following discussions. The modeling and measured results of the multifunctional hydrogel with Cu-Al metal pair are shown in Fig. 2f. An external AC voltage with an amplitude of 0.7 V with varying frequencies is applied to measure the impedance of the device (details in **Experimental Section**). The measuring results show great accordance with the modeling results (with parameters listed in Table S2), proving the efficiency of the proposed model. The EIS results of the hydrogel-based device with other metal pairs can also be found in Figs. S6-S10.

### Multifunctional Hydrogel as Energy Harvester with Stable DC Output, High Durability, and Resilience in Extreme Environments for Powering Outdoor IoT Systems

**Fig. 3:**
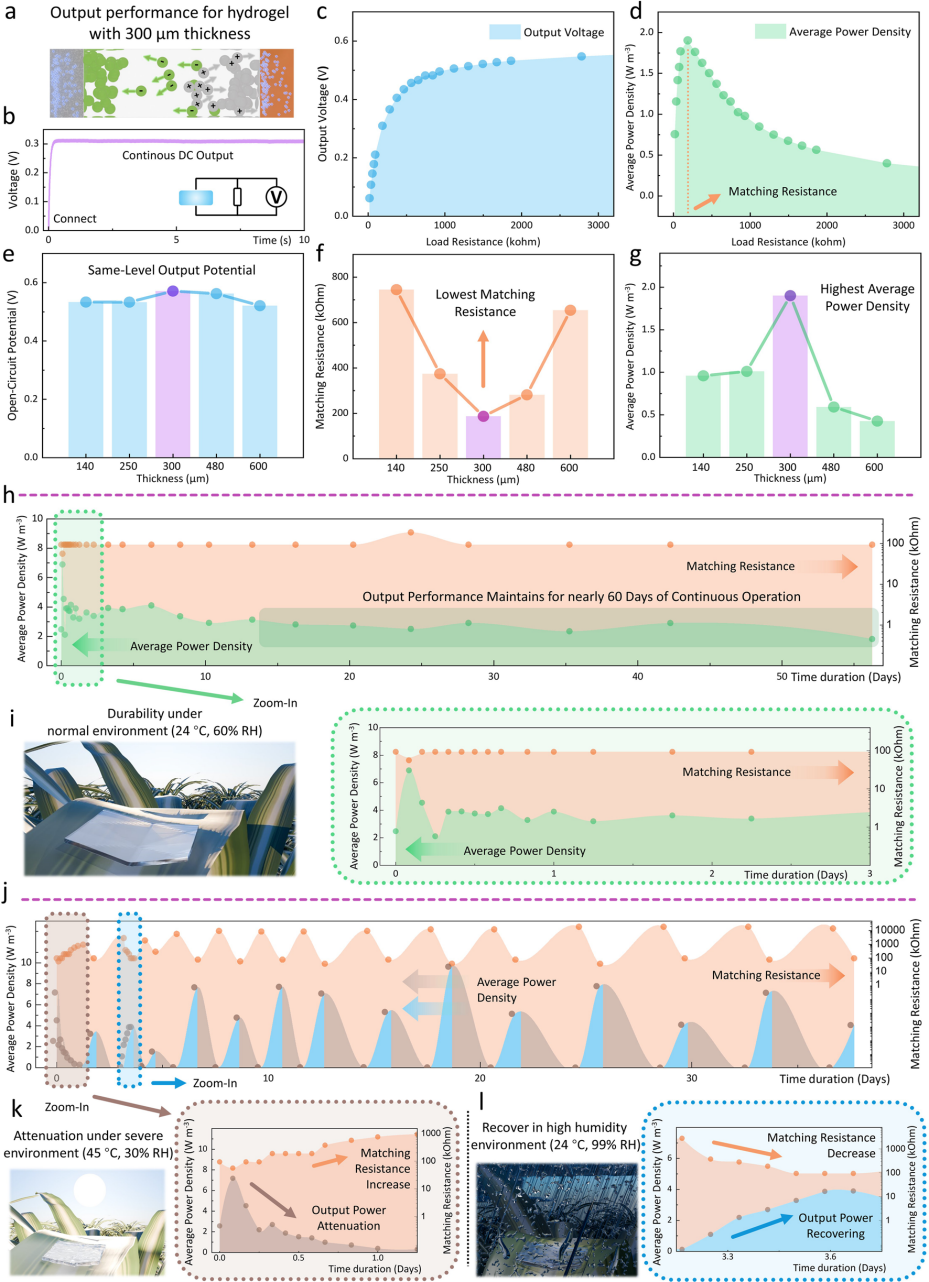
Characterization of multifunctional hydrogel output performance and its durability and resilience in extreme environments. **a-d** Characterization of the output performance for the hydrogel-based EH with 300 μm thickness: **a** Schematic illustration of the hydrogel-based EH. The oxidation–reduction reaction between ions and electrodes creates a stable potential for electrons to flow. **b** The generated continuous DC output when connected to the matching resistance. The variation of **c** output voltage and **d** average power density under varying load resistance. The matching resistance is the load resistance that realizes the largest output power. Comparison of the **e** saturated open circuit potential, **f** matching resistance, and **g** average output density for hydrogel-based EHs with different thicknesses (140, 250, 300, 480, and 600 μm), showing the 300-μm thickness device has the optimized output performance. (Detailed measured curves are provided in Figs. S12-S16) **h-i** The durability of the hydrogel-based EH under a normal outdoor environment (24 °C and 60% RH). **h** Monitoring of output performance attenuation for the hydrogel-based EH under 56.25 days of continuous operation. (detail testing results for each data point are provided in Figs. S19 and S20). **i** An illustration of one hydrogel-based EH placed in a normal environment with zoom-in curves to show the variation of its output performance in the first three days. **j-l** The durability of the hydrogel-based EH under severe environment (45 °C and 30% RH) and self-recovering under high humidity environment (24 °C and 99% RH). **j** Monitoring of output performance attenuation for the EH under 37.67 days of continuous operation with a total of 13 attenuation-recover cycles. **k** Zoom in to show the attenuation of the ionic hydrogel under severe environments. **l** Zoom in on the self-recovering process of the ionic hydrogel under a high-humidity environment, such as on rainy days. The capacitor charging performance for the multifunctional hydrogel at each time period in **i**, **k,** and **l** is also tested and provided in the Fig. S21

As previously discussed, the oxidation–reduction reactions between ions and electrodes in the multifunctional hydrogel (Fig. 3a) generate a stable and continuous DC output (Fig. 3b), which can become a desired power source for IoT systems. In addition to the metal pairs selection, the thickness of the ionic hydrogel also influences the output performance of the hydrogel-based EH, especially the matching resistance and power density. Therefore, such phenomena are evaluated to determine the optimized thickness. Figure 3c, d shows the characterization results of a 300 μm thickness ionic hydrogel. With increasing load resistance (from 18.6246 to 3.708 MOhm), the measured voltage keeps increasing (from 61.6 to 571 mV) and becomes close to its open-circuit saturated potential, while its output power reaches the maximum value at its matching resistance (186.617 kOhm). As a low-cost device with scalable fabrication targeting large-area applications, the average power density is a crucial factor to consider and, therefore, is the main parameter to be optimized. Ionic hydrogels with different thicknesses, including 140, 250, 300, 480, and 600 μm, are all measured in the same manner (**Experimental Section**), with detailed results provided in Figs. S12-S16. Besides, in Fig. S17 and Note S3, we have also discussed that further applied external load force will not alter the output performance by improving the contact between the metal layers with the ionic hydrogel, meaning the fabricated hydrogel-based EHs already shows good stability and reliability. Figure 3e-g depicts the comparison of the matching resistance, saturated output potential, and average power density for those hydrogel-based EHs with various thicknesses. As shown in Fig. 3e, the open circuit potential exhibits minimal variation across different hydrogel thicknesses. This consistency arises from the fact that the open circuit potential is predominantly dictated by the chemical reactions rather than the thickness of the hydrogel, which remains the same as the materials that are not changed. However, it’s noteworthy that the matching resistance and average power density do fluctuate with varying thicknesses. In the initial stages, as the thickness of the ionic hydrogel increases, there is a corresponding rise in the amounts of ions within it. This leads to an increase in current flow, indicated by a decrease in matching resistance. However, as the thickness continues to increase, the length of ion channels within the hydrogel electrolyte also increases, which poses a hindrance to the movement of ions, causing a subsequent decrease in current flow and an increase in matching resistance. To reconcile the effects stemming from ion amounts and ion channel length, as illustrated in Fig. 3e, g, it can be noticed that the 300 μm-thick hydrogel demonstrates the most favorable balance. This thickness showcases the lowest matching resistance and the highest average power density compared to the other thicknesses under consideration. Hence, it suggests that the 300 μm thickness represents an optimized configuration for the hydrogel-based EH, which achieves an average power density of 1.9 W m^−3^.

Furthermore, compared to previous EHs, such average power density does not require external stimulation like vibrations, winds, or sunlight. The hydrogel-based EH can keep stable output by harvesting chemical energy from the oxygen and water, which are everywhere in the ambient environment, through the following dominant cathode reaction at the copper cathode electrode (which can be noticed from the cyclic voltammetry (CV) curve shown in Fig. S18):

$$ O_{2} + 2H_{2} O + 4e^{ - } \to 4OH^{ - } $$

To assess the long-term performance of the hydrogel-based EH, we first tested its durability performance under normal outdoor environments (24°C, 60% RH) for nearly two months (56.25 days). It is worth highlighting that this is not just to test the self-attenuation of the hydrogel under such an environment, but the device is also always linked to a 4 F supercapacitor to be kept in the powering mode that continuously generates output in 2 months (details provided in **Experimental Section**). The variance of average power density and matching resistance in these two months, as the two most direct parameters to evaluate the performance of EHs, are selected and shown in Fig. 3h (detail testing results for each data point are provided in Figs. S19 and S20). It can be noticed that there is some fluctuation in both the matching resistance and the average power density of the hydrogel-based EH during the initial 3 days, but it gradually becomes stable and maintains consistent performance till the end, proving the good durability and stability of its powering capability. Figure 3i provides the details of the variance in average power density and matching resistance during the initial 3 days. On day 1, the matching resistance decreases while the average power density increases. This phenomenon can be attributed to the relatively abundant water content right after the hydrogel is fabricated, which promotes the movement of ions. As the water in the hydrogel gradually evaporates to a stable level, the output performance becomes stable. In large-area IoT applications, the hydrogel primarily operates in this state where the water content has evaporated to a stable level and the output performance has also stabilized. The capacitor charging performance of the capacitor is also evaluated in this process, which is provided in Fig. S21.

The resilience of the proposed multifunctional hydrogel has also been tested under simulated severe outdoor environments for 37.67 days with high temperature and drought conditions (45 °C, 30% RH), as shown in Fig. 3j. Similarly, the hydrogel-based EH is also always connected to a 4 F supercapacitor to be kept in the operation mode and generate power. Such severe environments lead to increased water loss of the hydrogel, consequently reducing its ion mobility. This results in an increase in matching resistance and a decrease in average power density (Fig. 3k). However, in the high humidity environment (24 °C, 99% RH), such as on rainy days, the hydrogel can automatically absorb moisture from the ambient environment and recover to its normal state. This, in turn, results in a decrease in matching resistance and an increase in average power density, which can be noticed in Fig. 3l. A total of 13 attenuation and self-recovering cycles have been tested for the hydrogel-based EH, after which the hydrogel can still be recovered to an average power density same as its initial level.

Based on the stability and durability testing results, the energy density generated by the hydrogel-based EH under normal outdoor environments (24 °C, 60% RH) can also be estimated. From Fig. 3h to Fig. 3i, it can be noticed that the power density of the EH, at most of the time, can be treated as stable without suddenly and unforeseen abruptly changes. Besides, any variations in power density can also be assumed to be nearly linear—either increasing or decreasing gradually from one measurement interval to the next. Because even significant fluctuations in output performance can be observed under extreme environmental conditions (45 °C, 30% RH and 24 °C, 99% RH), as shown in Fig. 3j-l. These fluctuations still followed a predictable, nearly linear pattern over extended periods (e.g., from 8–24 h in Fig. 3k and 76–88 h in Fig. 3l), even under these harsh conditions. Addtionaly, the device is continuously generating power in DC mode. Therefore, the total energy density it generates under normal outdoor environments (24 °C, 60% RH) can be calculated through:

$$ E_{total} = \mathop \sum \limits_{i = 1}^{n - 1} \left( {\frac{{P_{i} + P_{i + 1} }}{2}} \right) \times (t_{i + 1} - t_{i} ) $$

where *P*_*i*_ represents the power density measured at the *i-th* time point, and *t*_*i*​_ represents the time at which the *i-th* power density measurement was taken. The utilization of this formula is also based on the experiment results that the output performance can be treated for the 56.25 days of continuous charging shown in Fig. 3h, the total energy density is 1.36 × 10^7^ J m^−3^. The above results show that the proposed multifunctional hydrogel, for its good durability, recoverability, and stable output without the requirement of specific stimulations such as vibrations, winds, or sunlight, makes it an ideal candidate for powering the outdoor IoT monitoring systems.

### Multifunctional Hydrogel as Self-Powered Real-Time and Noninvasive Leaf RWC Sensor to Monitor Outdoor Plants’ Health Status

Apart from its application as a stable EH, the multifunctional hydrogel can also serve as a noninvasive self-powered sensor for detecting water content in leaves. Traditionally, there are two methods to assess leaves’ real-time RWC on-site and determine their health status. The first approach involves utilizing multispectral image-based sensors to analyze the absorption intensity of plant leaves at various wavelengths [101, 102]. However, this method faces several challenges. Not only does it require high power consumption, making it difficult to implement IoT monitoring on a large scale, but it also has increased cost by necessity for fabricating specific wavelength lenses. The second method relies on ultrasonic waves, examining changes in the intensity and frequency of the ultrasonics after penetration of the leaf compared to their initial emission to analyze the water content within the leaf [103]. Yet, this method also suffers from high power consumption, typically needing around 20 V of high-frequency voltage to drive the piezoelectric plate to emit ultrasonic waves of sufficient intensity. Moreover, this invasive technique may cause some damage to the plant. A detailed comparison table is provided in Table S4.

One of the complications in noninvasively monitoring leaf water content lies in the fact that the moisture in the leaf is primarily distributed within the inner layers of plants, specifically the spongy mesophyll layer and veins. This distribution makes it challenging to gauge internal water content through surface characteristics alone. A common method exists to characterize changes in leaf water content, viewing the leaf itself as a high-impedance electrolyte with an electrochemical structure. By performing EIS analysis on the leaf, its impedance variations at different frequencies can be used to understand changes in its water content [104, 105]. However, the EIS measurements can only be performed in the laboratory using the equipment, which is unable to do on-site sensing and is impossible for large-area applications in outdoor environments. Inspiring from this principle, the leaf can be treated as an entire part of an electrolyte and be directly brought into the electrochemical system through contact with the hydrogel, which is also an electrolyte. Thereafter, the water content variance inside the leaf can be detected through the variance of the electrochemical model, which is directly noticeable from the variations of the measured voltage output generated by the system.

**Fig. 4:**
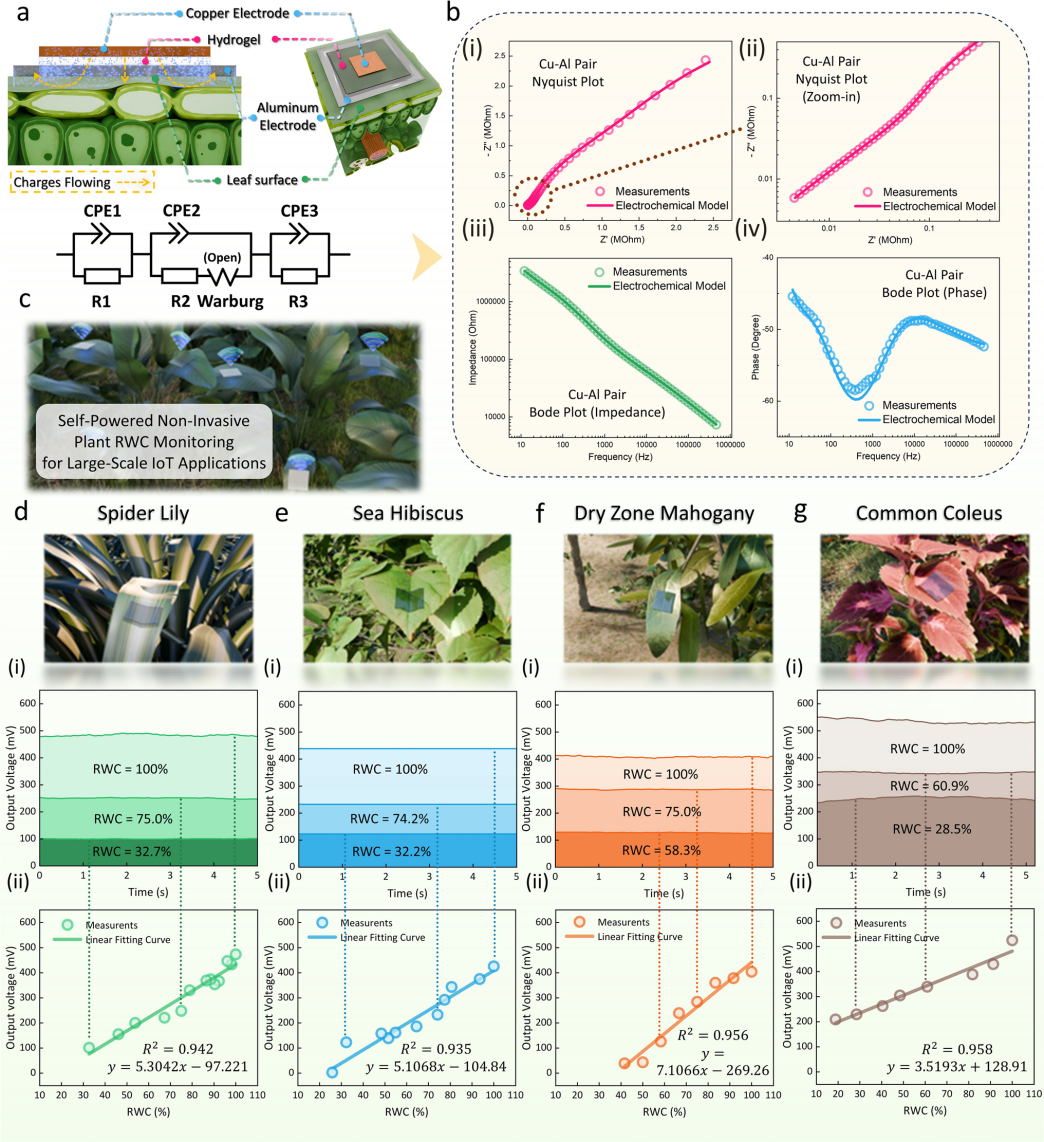
Self-powered noninvasive leaf RWC monitoring based on the multifunctional hydrogel. **a-c** Illustrations showing the ionic hydrogel can be directly attached to the leaf and the basic characterization of the modified electrochemical model. **a** The cross-section view shows the hydrogel directly contacts with the leaf to introduce the leaf to the whole system, as well as the corresponding electrochemical circuits. **b** The modeling results show good accordance with the measurements. Detailed discussion is provided in Note S4. **c** A demonstration of how noninvasive leaf RWC sensing can be applied in large areas for future outdoor IoT monitoring. **d-g** RWC measurement results for different types of leaves (**d** Spider Lily, **e** Sea Hibiscus, **f** Dry zone mahogany, and **g** Common Coleus) based on the variation of self-generated potentials of the ionic hydrogel, with **(i)** shows the data obtained for 5 s has good stability and **(ii)** shows the linear fitting results

Figure 4a illustrates how the hydrogel-based sensor is attached to the leaf surface for monitoring leaf RWC. In this scenario, the copper electrode is still placed on top of the hydrogel, for it’s the main interface where chemical reactions happening to generate a static potential and make the sensing system self-powered. Different from previous hydrogel-based EH, the aluminum electrode is now positioned around the hydrogel, making contact with the leaf surface but not with the ionic hydrogel (Fig. S22). In this configuration, the hydrogel and the leaf function as an entire electrolyte, where charges flow from the copper electrode through the hydrogel to the leaf and then to the aluminum electrode. Since the main reaction interface remains unchanged, the saturated open-circuit potential between electrodes stays at the same value as the previous EH, around 0.6 V. This similar structure ensures compatibility with previous analyses while expanding the utility of the hydrogel in interfacing directly with leaves. The electrochemical model for this system with further introducing leaves is also shown in Fig. 4a. Similarly, two CPE elements are used to describe the non-ideal interface between the electrode layers and the hydrogel or the leaf surfaces. One major difference here to be highlighted is the Warburg diffusion element. In the electrochemical model proposed in Fig. 2e, considering only the ionic hydrogel is the electrolyte, which has a relatively thick layer near the electrode surface, and ions are not spatially restricted in their diffusion, the Warburg short element (Ws) is applied. However, when including the leaf in the electrolyte, which has poor ion mobility, increased spatial resistance, and larger transverse area compared to the vertical depth, the Warburg open element (Wo) should be applied instead. In addition, the contact resistance also increases because the leaf surface has more complex microstructures and fewer conductive ions compared to the hydrogel, which can be noticed from the parameters listed in Table S3. The EIS measurement results of the updated system, which includes the leaf, are also compared with the modeling results in Fig. 4b. It can be noticed that the proposed model is in good accordance with the measuring results, proving its efficiency. Simultaneously, such results also prove that the whole leaf is successfully introduced to the system, and the sensors can extract its characteristics without invading it. Such a self-powered RWC monitoring method with low-cost and scalable fabrication can be more easily applied in large-scale outdoor IoT systems compared to traditional methods (Fig. 4c and Table S4).

The hydrogel-based sensor is applied to measure 4 different plants (spider lily, sea hibiscus, dry zone mahogany, and common coleus) to verify the feasibility of monitoring leaf RWC, as shown in Fig. 4d-g (photos provided in Fig. S23). The RWC of a leaf can be calculated with the following equation:

$$ RWC \left( {\text{\% }} \right) = \frac{{W_{C} - W_{D} }}{{W_{T} - W_{D} }} \times 100 $$

In the above equation, W_C_ represents the current weight of the leaf when measuring, W_D_ is the dry weight of the leaf, and W_T_ is the turgid (re-saturation) weight. The detailed process for calibrating the leaf RWC and measuring the sensed voltage output of the hydrogel-based sensor under that RWC can be found in the **Experimental Section.** A fixed load of 2.52 MOhm is applied to measure the output voltage from the system. Since the open-circuit potential always remains steady, as explained above, when the impedance of the whole system is altered, the measured voltage also alters correspondingly. Figure 4d-g(i) demonstrate that the output voltage of the hydrogel-based sensor remains steady at a constant value at a specific RWC value of leaves. Figure 4d-g(ii) show the results of the output voltage under different RWC values of leaves. The results from all four different plant leaves demonstrate that as the RWC increases, the output voltage of the hydrogel-based sensor also increases. This is because, as the water content in the leaf increases, the ion mobility in the leaf-hydrogel electrolyte also increases, resulting in lower impedance of the system and, thereafter, higher measured output voltage. The observed results indicate a commendable linear relationship from the value of R^2^ (R-squared) between the sensor’s output and the leaf’s RWC. This self-powered leaf RWC sensing approach offers a more efficient, cost-effective, and noninvasive method for monitoring leaf water content, which benefits large-area self-sustained IoT outdoor plant monitoring.

**Fig. 5:**
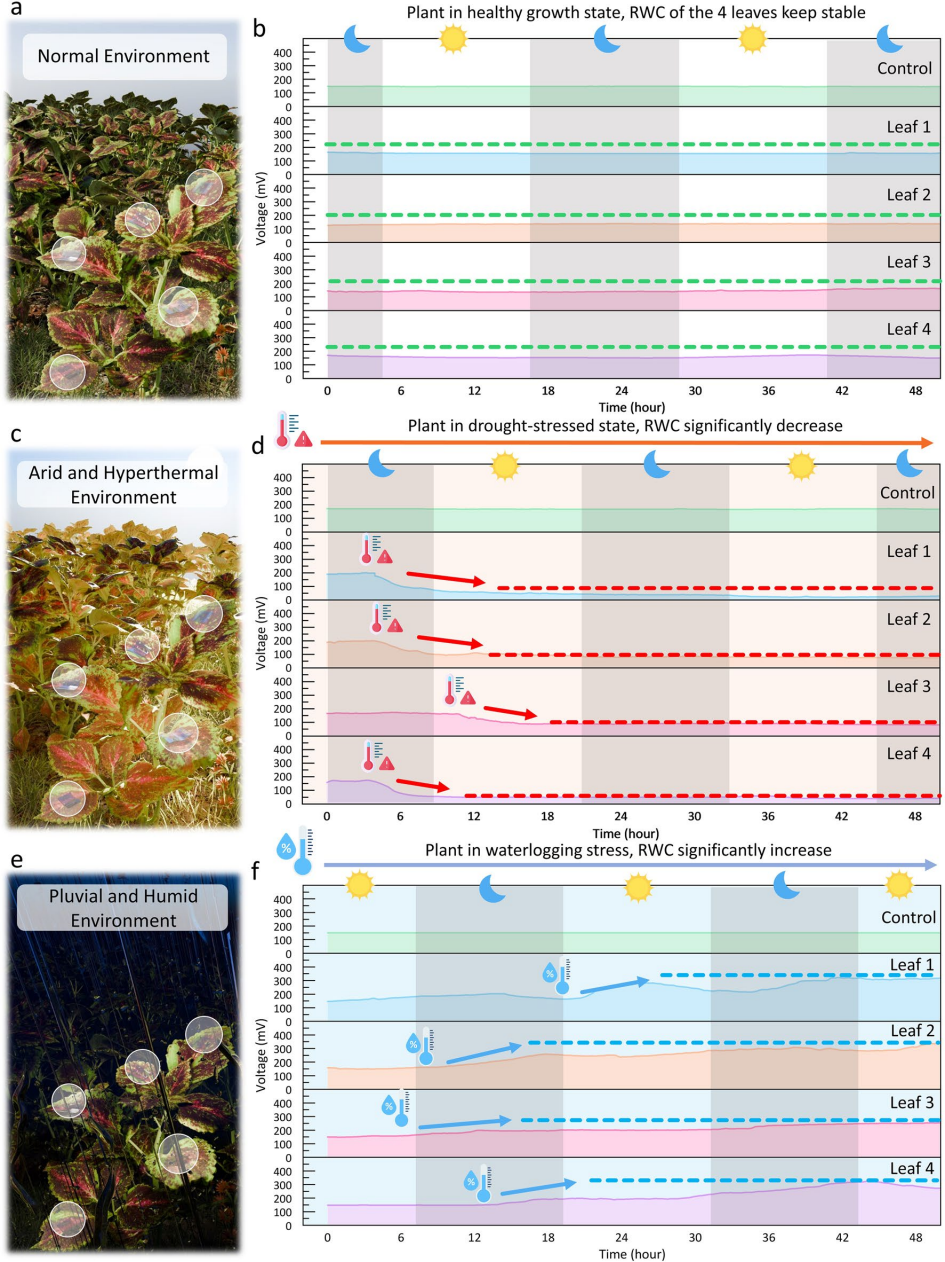
Long-Term IoT monitoring of leaves RWC by the multifunctional hydrogel under three environments.** a** Illustration of the hydrogels placed on a plant in a normal environment. **b** The signals from four leaves on the plant keep stable for 48 h, meaning the plant is growing healthily, and no intervention is required. **c** Illustration of the plant in the arid and hyperthermal environment. **d** The signals from leaves start to fall after 4 h, meaning the plant becomes extremely water-deprived and watering is required. The severe conditions are applied manually with high-power light source, as discussed in the **Experimental Section**. The sun and moon mean the actual diurnal variations in the environment. **e** Illustration of the plant in these extreme flood conditions. **f** The signals from leaves start to increase after 6–12 h, meaning improving drainage and elevating the soil is required. The severe conditions are applied manually with excessive watering, as discussed in the **Experimental Section.** The sun and moon mean the actual diurnal variations in the environment

To demonstrate how the application of such an RWC monitoring system can give useful information for smart farming in the outdoor environment, three different long-term monitoring scenarios are applied. These three scenarios include a normal environment (Fig. 5a, b) in which the plant grows healthily and two severe environments (Fig. 5c-f) where human intervention is required to protect the plant. Under the stimulating environments, four hydrogel-based sensors are attached to four different leaves on a plant to monitor the RWC, and one hydrogel is placed in the environment near the plant as the control group. Under the simulated normal environment (24 °C, 60% RH), as shown in Fig. 5b, the sensing voltages from four leaves remain stable for 48 h, indicating that the plant is in a healthy growth state. To manually simulate a hot and drought environment, a 400-lm incandescent lamp is suspended 40 cm above the plants from 0 h till the end of the experiment to induce hot and drought stress directly to the plant. As shown in Fig. 5d, the output voltages from sensors attached to the four leaves start to drop significantly after keeping stable for around four hours. At the same time, the output from the control group remains steady, meaning such variance from the four sensors is mainly caused by the intrinsic changes in the leaves rather than the hydrogel. This indicates the plant itself is starting to fail to maintain its water content after 4 h in such a severe environment and is in a drought-stressed state, where human intervention is urgently required. To manually simulate a waterlogging environment, we excessively water the plants every 12 h from 0 h, providing far more water than they require for normal growth. As depicted in Fig. 5f, over 48 h, the output voltage of the hydrogel-based sensor attached to the 4 leaves gradually surpasses the normal RWC value. This indicates that the RWC of the leaves gradually exceeds the normal healthy level, alerting an improving drainage and elevating of the soil is required.

### Self-Sustainable Outdoor IoT Smart Farming Systems Realized by the Multifunctional Hydrogel

**Fig. 6:**
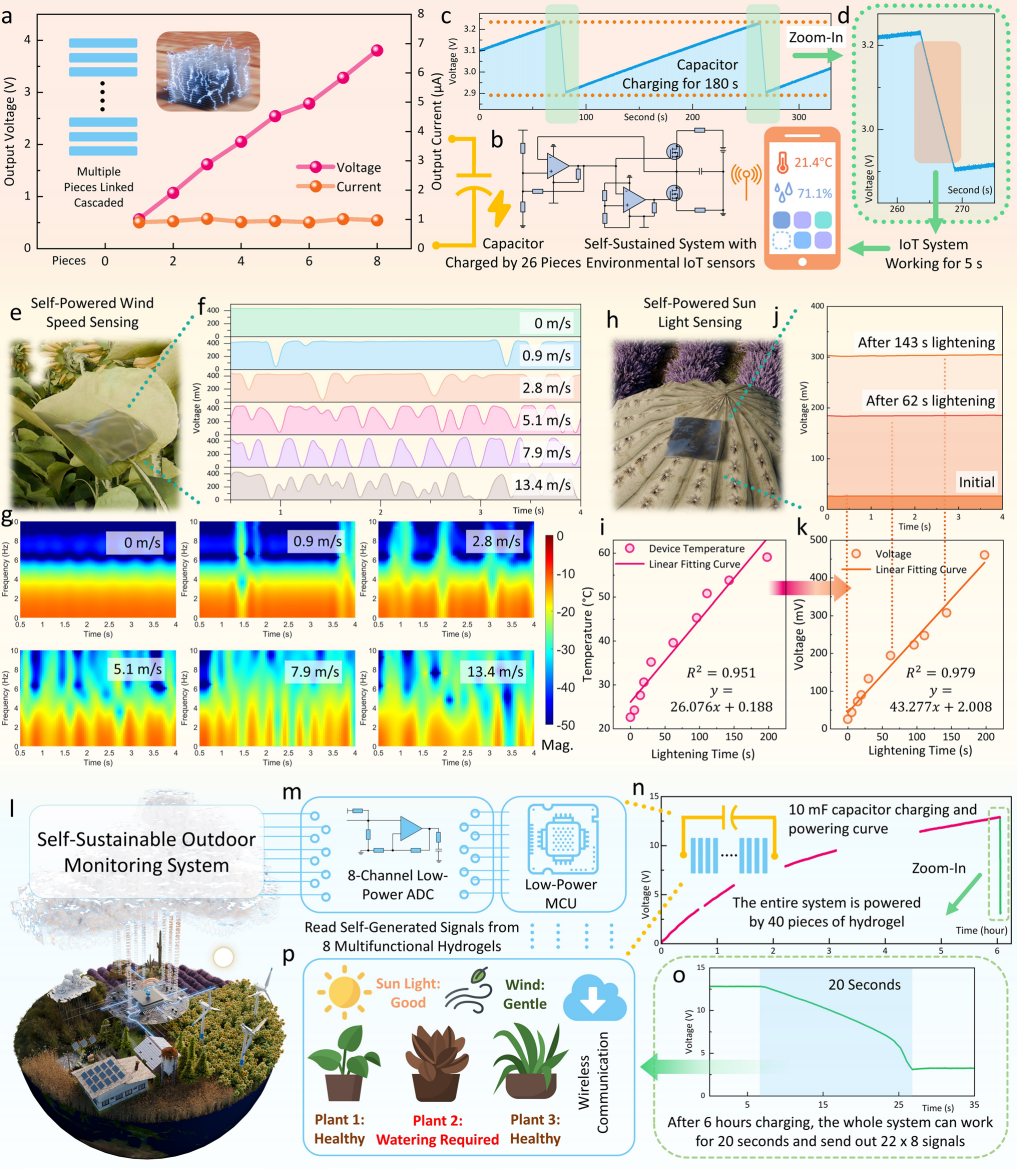
Multifunctional Hydrogel-Based Self-Sustainable IoT outdoor plant monitoring systems. **a-d** A self-sustainable IoT monitoring system with an automatic charging circuit and commercial temperature and humidity sensor powered by the multifunctional hydrogel: **a** The power output can be increased by cascading multiple pieces of multifunctional hydrogels. **b** The circuit diagram for the automatic charging control circuit, with details provided in Note S5. **c** The charging and powering curves of the capacitor: the capacitor can be fully charged for about 180 s; after charging, the capacitor can work for about 5 s to power the whole system. **d** Zoom in on the green zone. During this period, the self-sustainable system can sense the environmental temperature and humidity and send the information wirelessly to a mobile phone through Bluetooth. **e-p** Self-sustainable multimodal environmental sensing and plant health monitoring system enabled by the multifunctional hydrogel: **e** Schematic illustration of the multifunctional hydrogel can also be applied as a self-powered wind speed sensor on the leaf. **f** Output voltages of the sensor under different wind speeds. **g** Time–frequency analysis of the sensing signals. **h** Schematic illustration of the multifunctional hydrogel as a self-powered sunlight sensor. **i** The lightening leads to an increase in device temperature. **j** Output voltages generated at a specific lighting time keeps stable. **k** The sensed output voltage increases with the lightening time with good linearity. **l** Illustration of the multifunctional-hydrogel-enabled multimodal self-sustainable outdoor monitoring system. **m** Illustration of a designed MCU with wireless communication for collecting 8-channel self-generated signals from 8 multifunctional hydrogel-based sensors, with details provided in Note S5. **n** Curves of the 10 mF capacitance charging by 40 pieces linked cascaded hydrogels for 6 h and **o** powering the MCU for wireless sending out 8 channel signals, with each channel having 22 data points. **p** Information on sunlight, wind speed, and the plant status is transferred to the host with wireless communication

The proposed multifunctional hydrogel can realize multiple self-sustainable outdoor monitoring systems for future smart farming for its advantages in generating stable output in all outdoor conditions and self-powered multimodal sensing capability. Firstly, the scalability and low-cost nature of the hydrogel-based EHs allow for the fabrication of multiple units that can be connected in series to further enhance their output power. As shown in Fig. 6a, connecting eight devices in series raises their output voltage to approximately 4 V, thereby enabling direct power supply to conventional IoT sensors that typically operate at 3.3 V. The capacitor charging performance can also be efficiently improved with cascading multiple hydrogels, as shown in Fig. S24. This multifaceted design offers a flexible, robust, and cost-effective solution for powering various IoT systems. Based on the preceding findings, a self-sustainable IoT sensing system with environmental sensors and wireless communication modules has been constructed. This system comprises a commercial IoT temperature and humidity sensor, SHT35, operating with an average power consumption of around 20 μW. It also includes a Bluetooth module, HC-05, for wireless transmission, which consumes an average working power of approximately 100 μW. Additionally, the system features an automated charging control circuit, the detailed components of which are illustrated in Fig. 6b. Within this circuit, a Schmitt trigger based on the LM393D low-power comparator is utilized to monitor the potential difference across a 500 μF capacitor and, in turn, to switch the output voltage. This regulation controls the conduction and cut-off of the MOSFET, thereby governing the charge and discharge loop of the capacitor. A more detailed discussion can be found in Note S5. Figure 6c illustrates the charging curve for the 500 μF capacitor applied in this circuit for powering the whole system by 26 cascaded pieces of hydrogels, along with the power supply curve for the entire system when the capacitor reaches the preset voltage. Figure 6d provides a zoomed-in view of the power supply curve. Upon charging to 3.2 V, the automatic control circuit allows the capacitor to power both the IoT sensor and the Bluetooth module. When the voltage of the capacitor drops below 2.9 V, a Schmitt trigger will help to ensure the capacitor enter the charging phase again, during which the hydrogel will charge the capacitor back up, ensuring the voltage rises above 3.234 V before discharging again. In this system, the hydrogels can charge for 180 s to enable the system to operate for 5 s, thereby transmitting the environmental temperature and humidity information to a mobile device. This configuration realizes a self-sustainable IoT environmental monitoring system, highlighting the effective integration of the hydrogel’s energy harvesting characteristics with the operational demands of the sensors. The arrangement ensures a balanced cycle between charging and data transmission, fostering efficiency in continuous outdoor monitoring.

Furthermore, by using different setups, the multifunctional hydrogels can also act as multimodal self-powered environmental sensors, such as wind speed sensing (Fig. 6e-g) and sunlight sensing (Fig. 6h-k). When it acts as a self-powered wind speed sensor, as shown in Fig. 6e, the aluminum electrode on the top is not fully adhered to the hydrogel but only a side of it, which can be separated when there is wind. At no wind conditions, it acts similarly to previous devices, for it is in contact with the ionic hydrogel, generating a stable voltage output (Fig. 6f). As the wind blows, the aluminum electrode and the hydrogel on the leaf surface become separated, causing the output voltage to drop to nearly zero. Based on such operations, different wind speeds introduce different frequency responses to the output voltages, as depicted in the time–frequency analysis in Fig. 6g. In addition, the multifunctional hydrogel can also be used for sunlight sensing by simply coloring the upper layer with black to absorb light radiation (Fig. 6h), with preparation details provided in the Experimental Section. As the exposure time to sunlight increases, the temperature of the device rises (Fig. 6i). This increase in temperature enhances ion mobility, which reduces the sensor’s impedance while maintaining a constant open-circuit voltage. Consequently, the output voltage read by a fixed load increases with rising lightning time, enabling self-powered sunlight sensing (Fig. 6j). Besides, the sensed voltage also shows a good linear correlation with the lighting time (Fig. 6k).

At last, a self-sustainable IoT multimodal monitoring system for outdoor smart farming applications can be realized entirely based on this multifunctional hydrogel (Fig. 6l). In this system, the self-generated signals from the multifunctional hydrogels are collected by a designed low-power microcontroller unit (MCU), as shown in Fig. 6m, with details provided in Note S5. 40 pieces of hydrogels are linked in series to charge a 10 mF capacitor, which is used to power the entire system (Fig. 6n). After charging for 6 h, the capacitor can reach approximately 13 V, sufficient to power the low-power MCU for around 20 s (Fig. 6o). During this period, eight-channel signals from the self-powered leaf RWC sensor, wind speed sensor, and sunlight sensor can be sent to the host wirelessly, with each channel having 22 data points. The system’s performance can be further improved through more cascaded hydrogels or optimized circuit design in the future, through which whether the environment is conducive to plant growth or the plant is in a healthy growing status can be monitored, helping the host determine if human intervention is required for those plants (Fig. 6p).

## Conclusions

In addressing global challenges such as climate variability, resource depletion, and increased food demand, smart farming emerges as a critical component of contemporary agricultural strategies. The integration of self-sustainable outdoor monitoring systems is vital within this framework. These systems offer continuous and autonomous monitoring capabilities that are crucial for adapting to environmental changes and enhancing management decisions. However, traditional EHs depend on intermittently available environmental energies, such as wind or sunlight, leading to highly unstable long-term outputs. Furthermore, the dual capabilities of energy harvesting and self-powered plant health monitoring have rarely been achieved in the same device for outdoor use, presenting significant challenges in system integration and fabrication complexity, which hinders their widespread deployment in outdoor settings. Moreover, the materials used in these devices often lack the necessary transparency, flexibility, and biocompatibility for application on plants.

In this work, a multifunctional hydrogel has been fully characterized and optimized to realize a self-sustainable multimodal IoT monitoring system solely based on this device for future smart farming applications. Based on the biocompatible and transparent ionic hydrogel composed of PVA, PAAm, LiCl, and Gly, together with Cu-Al metal pairs as its electrode layers, the fabricated device can self-generate a stable potential. This stable and continuous DC output can be more efficiently applied for powering commercial IoT devices, achieving an average power density of 1.9 W m^−3^. Due to its main reaction being based on harvesting the ambient oxygen and water, such output power can be maintained in normal environments (24 °C and 60% RH) for 56.25 days, continuously operating in powering a supercapacitor, where it achieves an energy density of 1.36 × 10^7^ J m^−3^. Furthermore, in severe conditions that could be faced in outdoor environments, such as scorching and arid environments (45 °C and 30% RH), it can self-recover to normal output on rainy days (24 °C and 99% RH) by absorbing the ambient water from the air. Such resilience is maintained after 13 cycles of fatigue and recovery in nearly 40 days. In addition to acting as a stable power supply, the multifunctional hydrogel can also realize real-time, self-powered, and noninvasive leaf RWC monitoring. By coupling the leaf as an electrolyte to the system, its RWC information can be directly obtained from the value of the self-generated voltages, which provides important information in evaluating plant health status. Besides, due to its scalable fabrication capability and low-cost nature, it can be easily cascaded with multiple pieces to power some complicated IoT systems or be applied in large areas. At last, a self-sustainable outdoor IoT system with self-powered plant monitoring and environmental wind and speed sensing has been fully realized solely by the multifunctional hydrogel.

In the future, such a multifunctional hydrogel characterized by stable power generation, multimodal self-powered sensing, high durability and resilience across various environments, along with scalability and biocompatibility, holds significant potential for widespread applications in outdoor smart farming.

## Supplementary Information

Below is the link to the electronic supplementary material.

Supplementary file1 (DOCX 86358 kb)
