# Supplementary material for: A Multifunctional Hydrogel with Multimodal Self-Powered Sensing Capability and Stable Direct Current Output for Outdoor Plant Monitoring Systems
Source: Nanomicro Lett. 2024 Nov 27;17:76. doi: 10.1007/s40820-024-01587-y (PMC11602912; doi:10.1007/s40820-024-01587-y)
Supplement: Supplementary file 1 — Supplementary file1 (DOCX 86358 kb) [file 40820_2024_1587_MOESM1_ESM.docx]

Supporting Information for

**A Multifunctional Hydrogel with Multimodal Self-Powered Sensing Capability and Stable Direct Current Output for Outdoor Plant Monitoring Systems**

Xinge Guo^1,2, †^, Luwei Wang^1,2, †^, Zhenyang Jin^1,2,†^, and Chengkuo Lee^1,2,3,4,^*

^1^ Department of Electrical & Computer Engineering, National University of Singapore, 4 Engineering Drive 3, 117576, Singapore

^2^ Center for Intelligent Sensors and MEMS (CISM), National University of Singapore, 5 Engineering Drive 1 Singapore 117608, Singapore

^3^ NUS Graduate School - Integrative Sciences and Engineering Program (ISEP), National University of Singapore, Singapore 119077, Singapore

^4^ Research Center for Sustainable Urban Farming (SUrF), National University of Singapore, Singapore 117558, Singapore

^†^Xinge Guo, Luwei Wang, and Zhenyang Jin contributed equally to this work.

* Corresponding author. E-mail: [elelc@nus.edu.sg](mailto:elelc@nus.edu.sg) (Chengkuo Lee)

**Supplementary Figures and Tables**


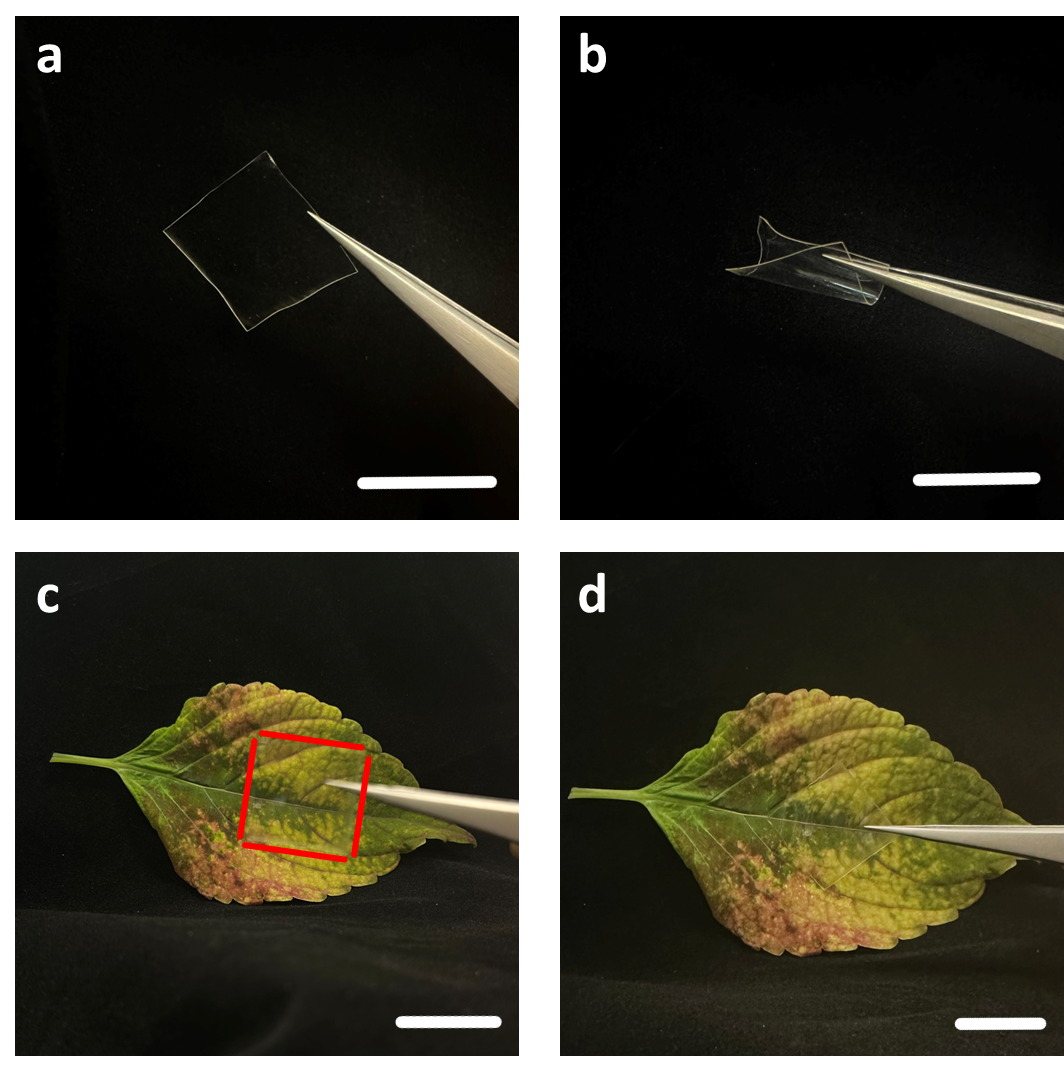


# Fig. S1 Images of (a, b) fabricated PVA/PAAm/LiCl/Gly ionic hydrogel and (c, d) the fabricated PVA/PAAm/LiCl/Gly ionic hydrogel placed above a leaf

The white scale bar for each figure stands for 2 cm. The PVA/PAAm/LiCl/Gly ionic hydrogels shown in these figures all have 300 μm thickness, the optimized thickness introduced in **Fig. 3**, and is applied throughout the manuscript. After molding, the fabricated PVA/PAAm/LiCl/Gly ionic hydrogel can be noticed as having good flexibility and transparency.


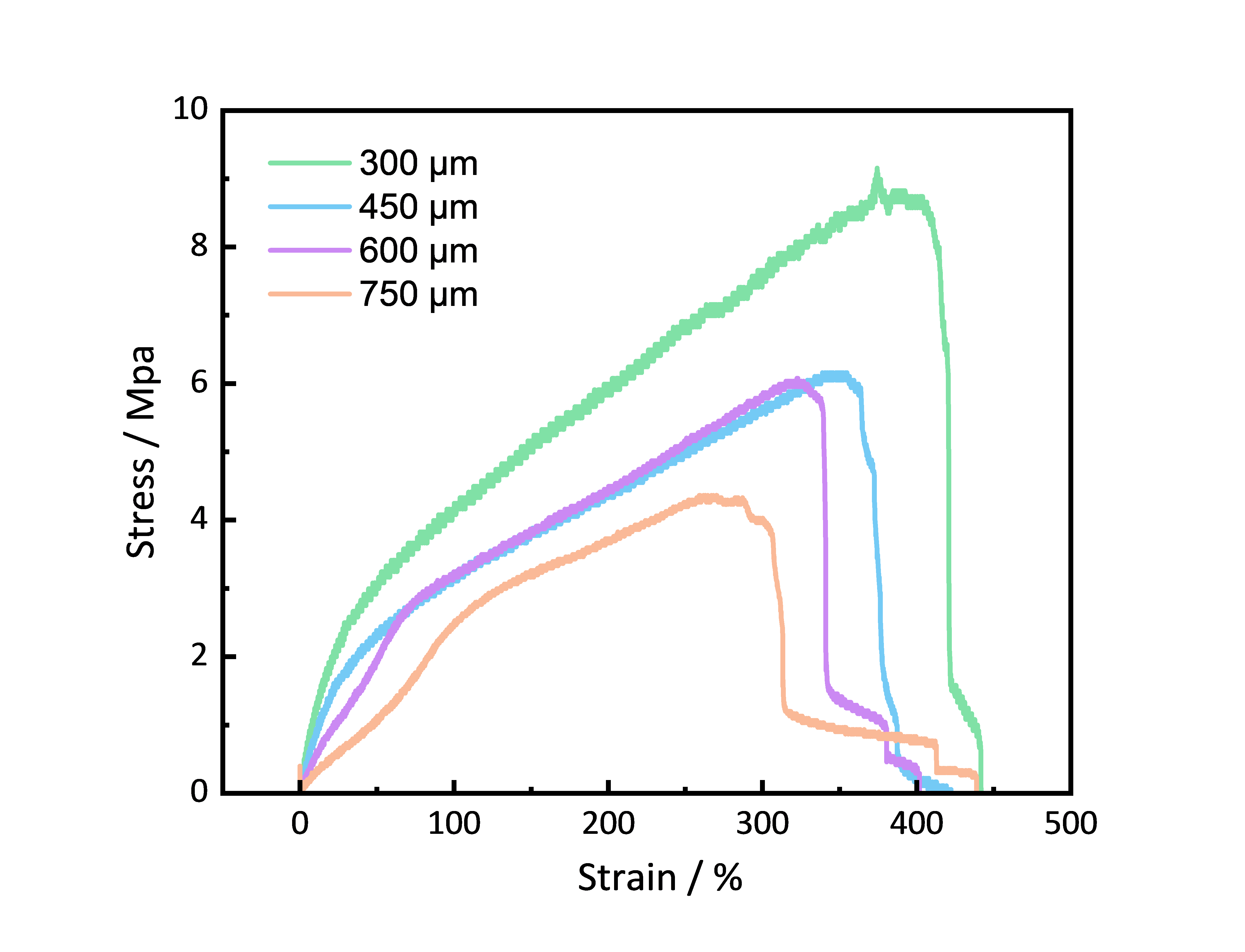


# Fig. S2 Mechanical properties of fabricated PVA/PAAm/LiCl/Gly ionic hydrogel with thickness varying from 300 μm to 750 μm


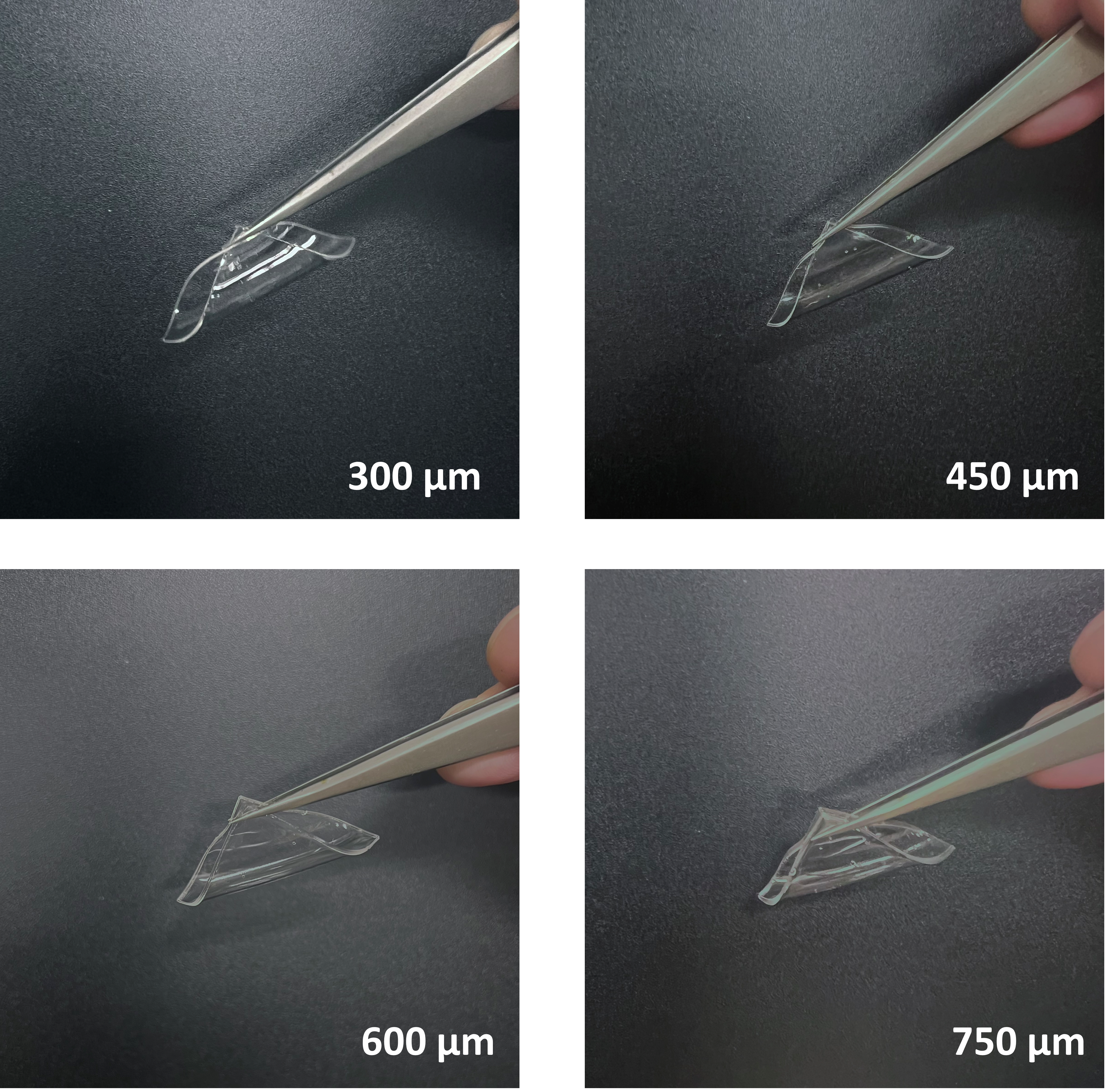


# Fig. S3 Flexibility of fabricated PVA/PAAm/LiCl/Gly ionic hydrogel with thickness varying from 300 μm to 750 μm. All the four hydrogels are 3 cm × 3 cm squares.

# Note S1 Characterization of PVA/PAAm/LiCl/Gly ionic hydrogel-based device with different metals as electrode materials for optimized voltage and response for energy harvesting and self-powered sensing

Regarding that different potential differences could be generated with respect to different metallic working electrode pairs due to their distinctive oxidation-reduction qualities, which typically arise from the faradaic process and are universally recognized by their standard cell potentials in analytical chemistry, we conduct an experiment in order to test the performance of six different metallic materials (Al, Ni, Zn, Cu, Ag, and Mo) in pairwise combination as electrodes along with the PVA/PAAm/LiCl/Gly hydrogel serving as the electrolyte of the ionic hydrogel-based device.

The choice of metal pairs (Al, Ni, Zn, Cu, Ag, and Mo) was driven by several factors including their electrochemical properties, cost-effectiveness, and compatibility with the hydrogel system.

1. **Electrochemical Properties**: The selected metals have differing redox potentials and capabilities to generate a potential difference when paired. For instance, the Cu-Al combination was found to provide an optimized balance between high output potential and fast build-up rate, essential for the device’s energy harvesting and sensing functionalities.

2. **Cost-Effectiveness and Commercial Availability**: The metals chosen are not only cost-effective but also readily available as commercial thin films. This availability simplifies the fabrication process and enhances scalability for large-scale applications. Metals like aluminum (Al-tape), copper (Cu-tape), and nickel (Ni mesh) are available in bulk and come in very thin layers (the thickness of the copper layer is 66 μm and the aluminum layer is 75 μm), providing excellent flexibility. This characteristic is crucial for ensuring that our devices can be easily integrated into various settings without compromising performance.

3. **System Compatibility**: The selected metals are compatible with the hydrogel matrix, avoiding potential issues like metal leaching or degradation that could compromise the hydrogel’s structure and function.

While inert electrodes like platinum or gold offer higher stability and durability, they would significantly increase the cost of the device. More importantly, the use of such inert electrodes would limit the necessary redox reactions that are essential for enhancing the output voltage of our device. Our design requires electrodes that can actively participate in redox reactions to improve the efficiency of the device as both self-powered sensors and energy harvesters.

The performance of the device, composed of various electrode pairs, is directly analyzed and compared based on their corresponding open circuit voltage curves, as shown in **Figs. S4** and **S5**. These curves are generated upon the electrodes' contact with the hydrogel and the establishment of the circuit loop. The parameters T and V, specifically noted in each sub-figure, denote the build-up time and the saturated open circuit potential for different electrode pairs. By examining **Figs. S4, S5, and 2c**, it is evident that the Cu-Al electrode pair exhibits the shortest build-up time and the highest saturated potential. This indicates optimal performance, undoubtedly making it the best choice for the device, whether utilized as a power source or a self-powered sensor. It is worth noting that the positivity or negativity of the potential curve only represents the potential difference between the specifically chosen anode and cathode. Switching the positions of the two electrodes in the same electrode pair will only change the polarity of the output curve without affecting the fundamental nature and characteristics of the electrochemical reaction.

On the other hand, the electrochemical impedance spectrum (EIS) curves plotted in both Bode and Nyquist formations are also measured with respect to different electrode pairs. Similarly, as introduced in **Experimental Section**, because the applied small excitation is a kind of AC sinusoidal signal, the exchange of the two electrodes in the same electrode pair has no influence on the attributes of the device's impedance. Thus, the same as the presentation way of the last experiment for potential tests, only one of these permutations is selected for illustration.


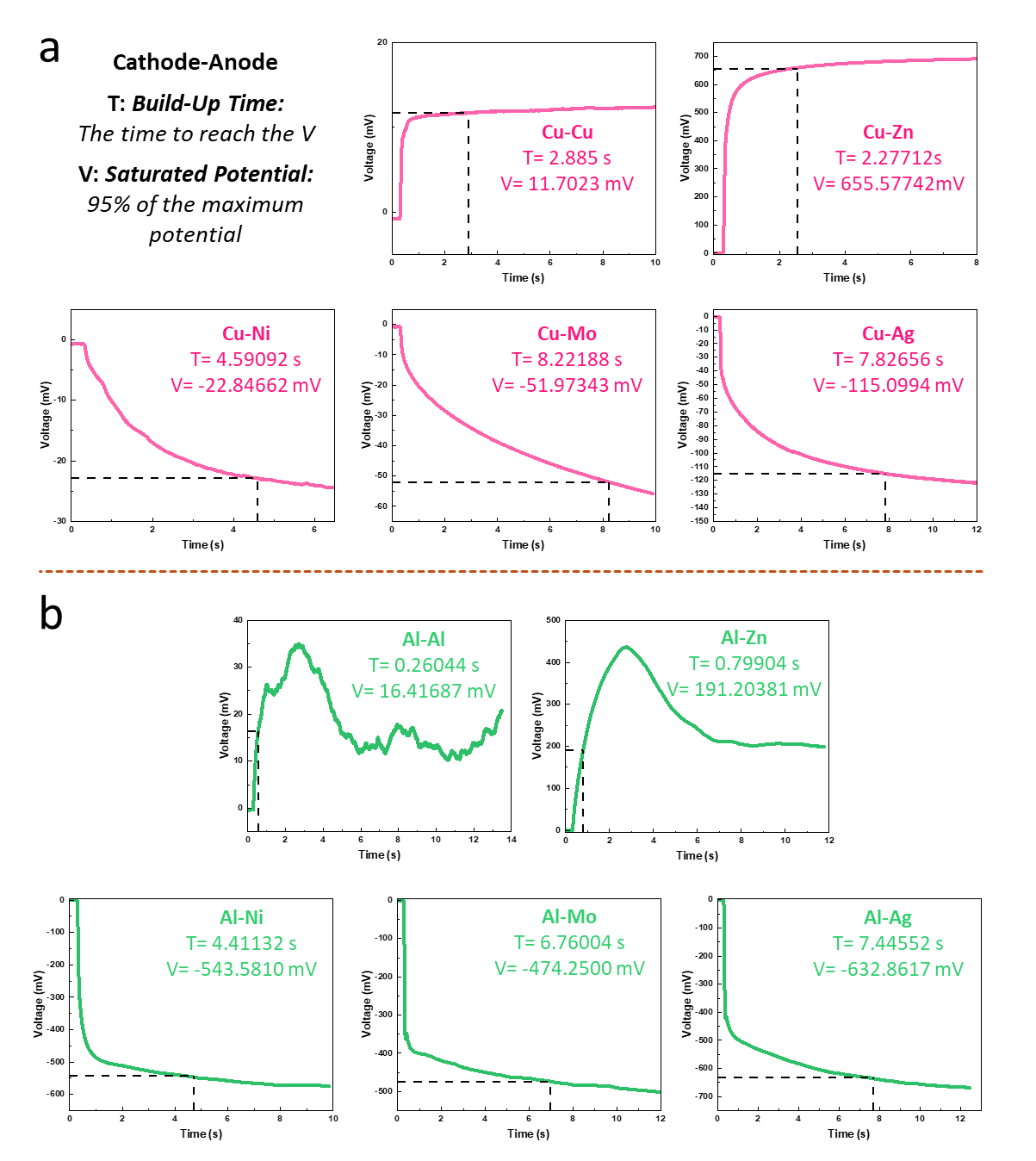


## Fig. S4 The potential of various electrode metal materials (Cu and Al as the cathode) after connection and the build-up time to reach 95% of the maximum potential (The results for Cu-Al metal pair is shown in Figure 2 in the main text): (a) Cu with other metals (Cu, Zn, Ni, Mo, and Ag), and (b) Al with other metals (Al, Zn, Ni, Mo, and Ag)


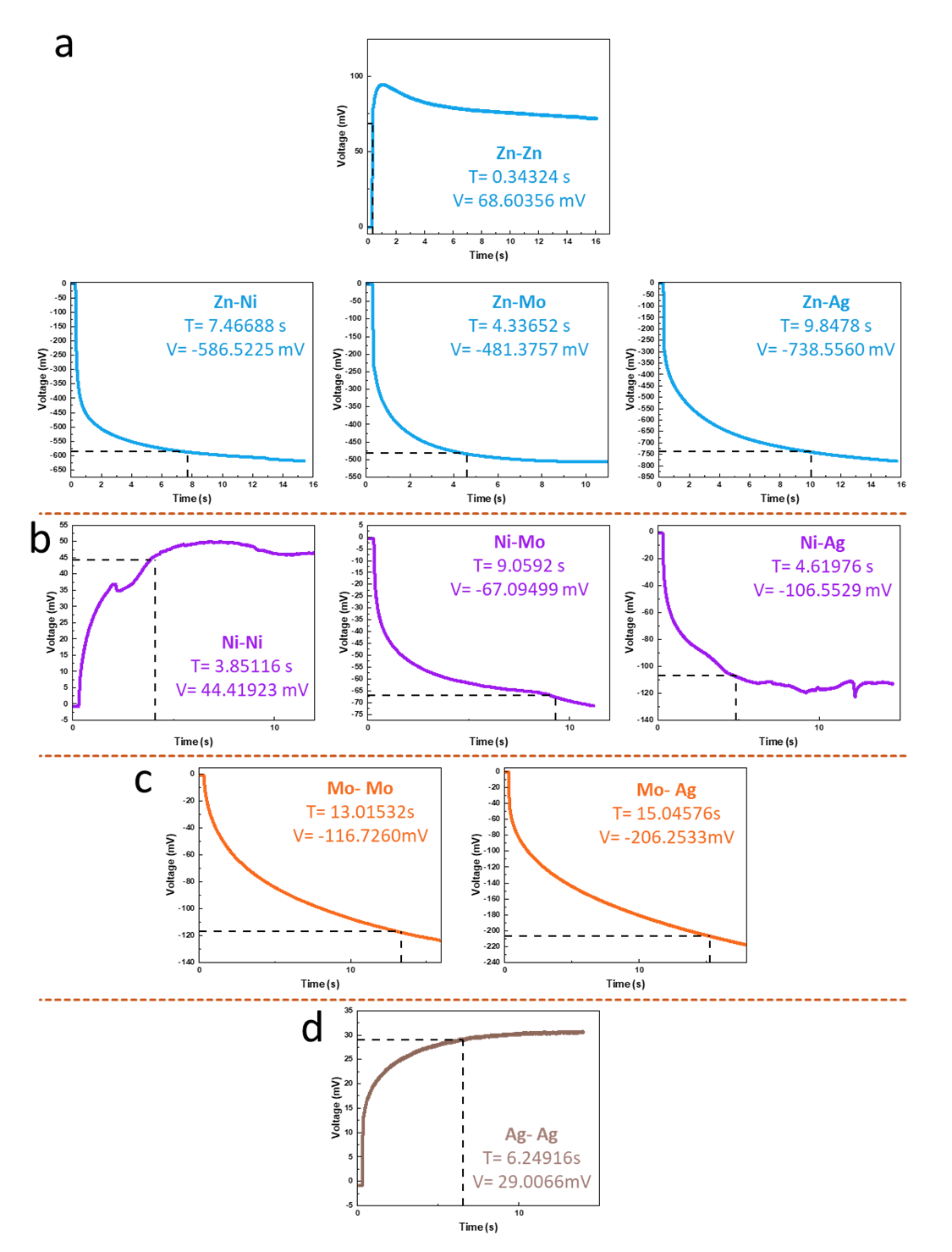


## Fig. S5 The potential of various electrode metal materials (Zn, Ni, Mo, and Ag as the cathode) after connection and the build-up time to reach 95% of the maximum potential: (a) Zu with other metals (Zn, Ni, Mo, and Ag), and (b) Ni with other metals (Ni, Mo, and Ag); (c) Mo with other metals (Mo and Ag); and (d) Ag with Ag

## Table S1 Summary of the potential of various electrode metal materials (Zn, Ni, Mo, and Ag as the cathode) after connection and the build-up time to reach 95% of the maximum potential, build-up time, and build-up rate

| Electrode Pair (Cathode-Anode) | Potential Value (mV) | Absolute Potential Value (mV) | Time (s) | Rate (1/s) |
| --- | --- | --- | --- | --- |
| Cu-Cu | 11.7023 | 11.7023 | 2.885 | 0.34662 |
| Cu-Al | 583.66411 | 583.66411 | 0.09988 | 10.01201 |
| Cu-Zn | 655.57742 | 655.57742 | 2.27712 | 0.43915 |
| Cu-Ni | -22.84662 | 22.84662 | 4.59052 | 0.21784 |
| Cu-Mo | -51.97343 | 51.97343 | 8.22188 | 0.12163 |
| Cu-Ag | -115.09936 | 115.09936 | 7.82656 | 0.12777 |
| Al-Cu | -583.66411 | 583.66411 | 0.09988 | 10.01201 |
| Al-Al | 16.41687 | 16.41687 | 0.26044 | 3.83966 |
| Al-Zn | 191.20381 | 191.20381 | 0.79904 | 1.2515 |
| Al-Ni | -543.58096 | 543.58096 | 4.41132 | 0.22669 |
| Al-Mo | -474.24998 | 474.24998 | 6.76004 | 0.14793 |
| Al-Ag | -632.86167 | 632.86167 | 7.44552 | 0.13431 |
| Zn-Cu | -655.57742 | 655.57742 | 2.27712 | 0.43915 |
| Zn-Al | -191.20381 | 191.20381 | 0.79904 | 1.2515 |
| Zn-Zn | 68.60356 | 68.60356 | 0.34324 | 2.91341 |
| Zn-Ni | -586.52247 | 586.52247 | 7.46688 | 0.13392 |
| Zn-Mo | -481.37574 | 481.37574 | 4.33652 | 0.2306 |
| Zn-Ag | -738.55597 | 738.55597 | 9.8478 | 0.10155 |
| Ni-Cu | 22.84662 | 22.84662 | 4.59052 | 0.21784 |
| Ni-Al | 543.58096 | 543.58096 | 4.41132 | 0.22669 |
| Ni-Zn | 586.52247 | 586.52247 | 7.46688 | 0.13392 |
| Ni-Ni | 44.41923 | 44.41923 | 3.85116 | 0.25966 |
| Ni-Mo | -67.09499 | 67.09499 | 9.0592 | 0.11039 |
| Ni-Ag | -106.55289 | 106.55289 | 4.61976 | 0.21646 |
| Mo-Cu | 51.97343 | 51.97343 | 8.22188 | 0.12163 |
| Mo-Al | 474.24998 | 474.24998 | 6.76004 | 0.14793 |
| Mo-Zn | 481.37574 | 481.37574 | 4.33652 | 0.2306 |
| Mo-Ni | 67.09499 | 67.09499 | 9.0592 | 0.11039 |
| Mo-Mo | -116.72598 | 116.72598 | 13.01532 | 0.07683 |
| Mo-Ag | -206.2533 | 206.2533 | 15.04576 | 0.06646 |
| Ag-Cu | 115.09936 | 115.09936 | 7.82656 | 0.12777 |
| Ag-Al | 632.86167 | 632.86167 | 7.44552 | 0.13431 |
| Ag-Zn | 738.55597 | 738.55597 | 9.8478 | 0.10155 |
| Ag-Ni | 106.55289 | 106.55289 | 4.61976 | 0.21646 |
| Ag-Mo | 206.2533 | 206.2533 | 15.04576 | 0.06646 |
| Ag-Ag | 29.0066 | 29.0066 | 6.24916 | 0.16002 |


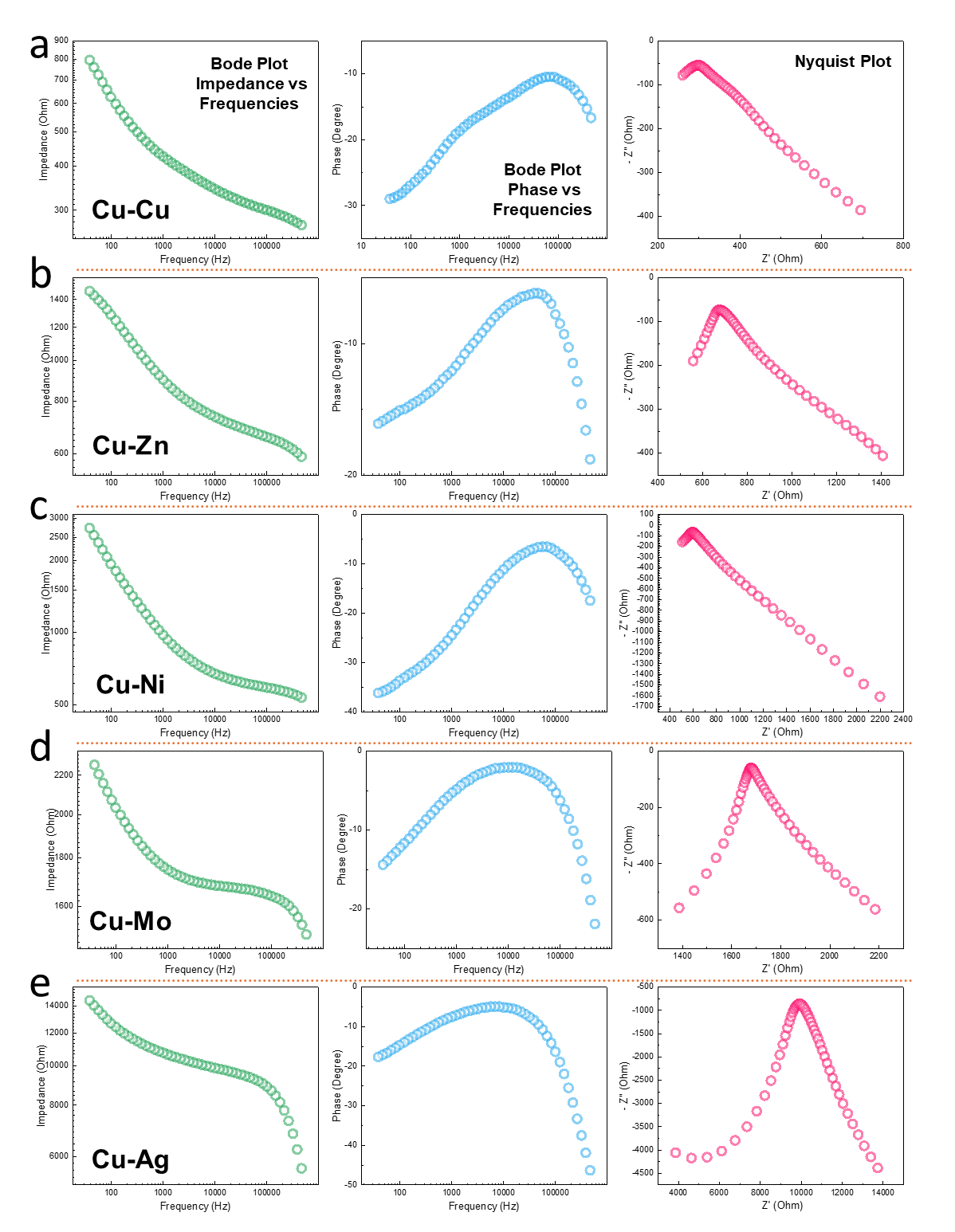


## Fig. S6 Bode plot of the absolute amplitude of the impedance under various frequencies (first row), bode plot of the phase under various frequencies (second row), and Nyquist plot of the measured results (third row) for the multifunctional hydrogel with Cu as cathode material, and (a) Cu, (b) Zn, (c) Ni, (d) Mo and (e) Ag as anode materials


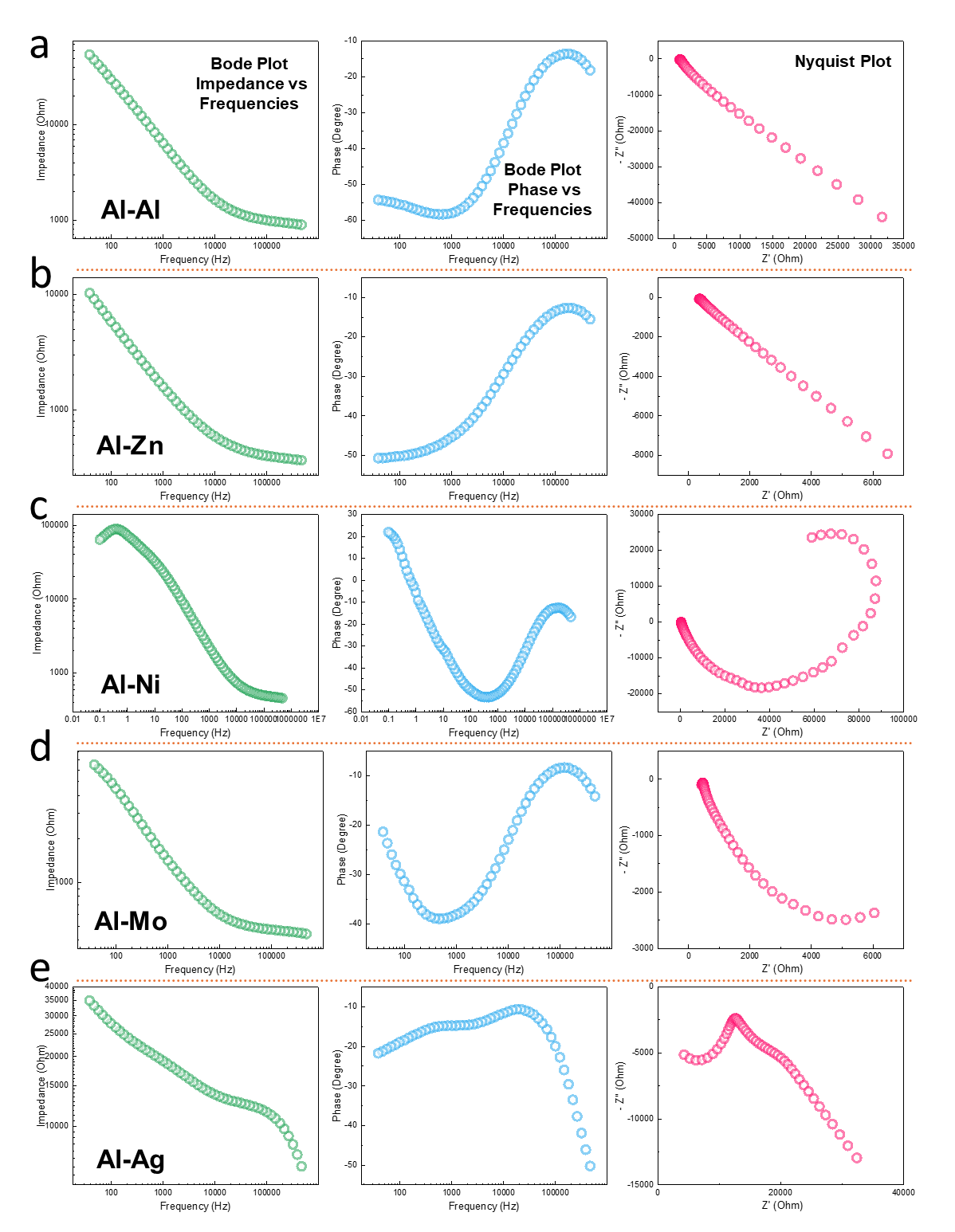


## Fig. S7 Bode plot of the absolute amplitude of the impedance under various frequencies (first row), bode plot of the phase under various frequencies (second row), and Nyquist plot of the measured results (third row) for the multifunctional hydrogel with Al as cathode material, and (a) Al, (b) Zn, (c) Ni, (d) Mo and (e) Ag as anode materials


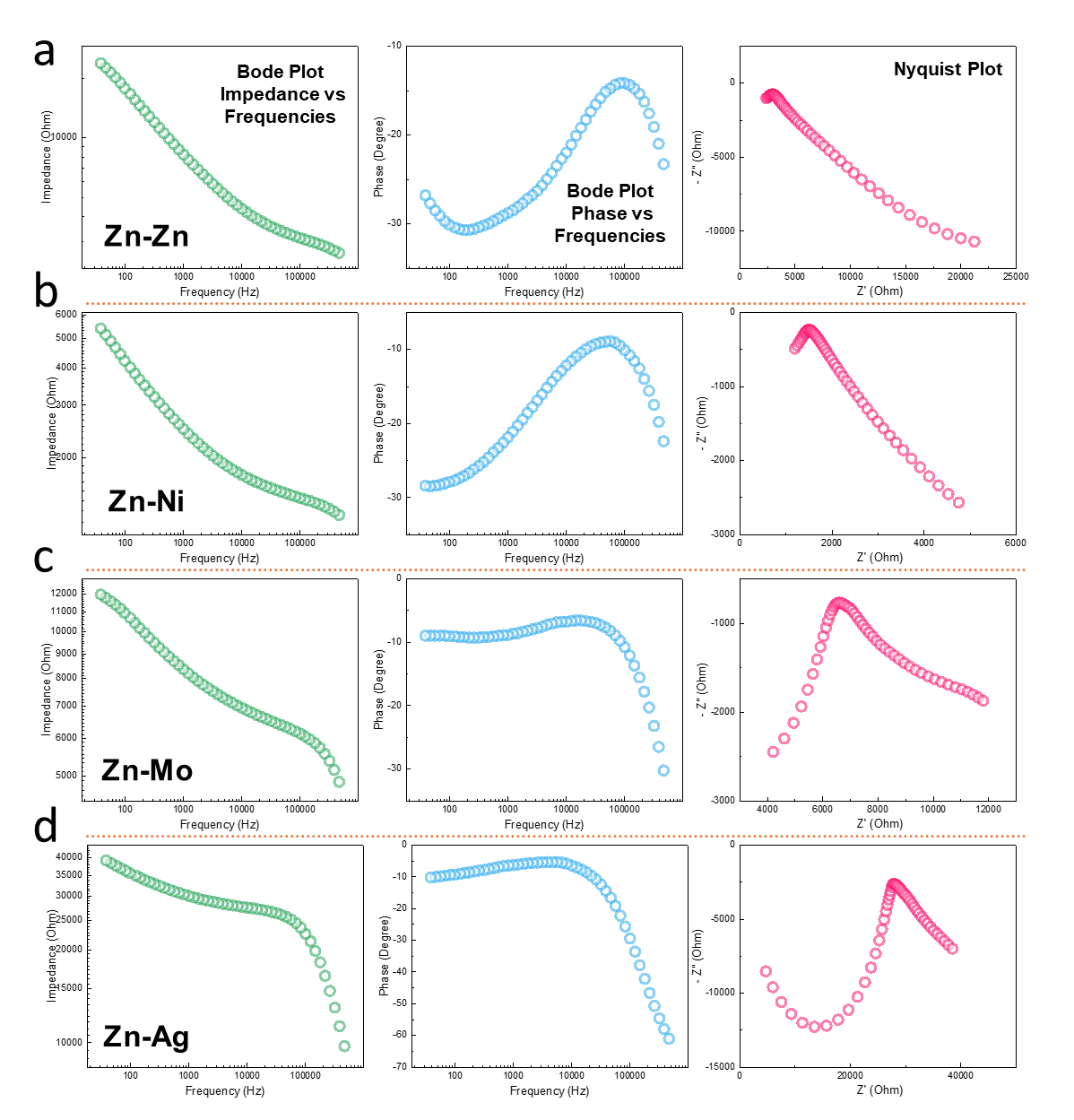


## Fig. S8 Bode plot of the absolute amplitude of the impedance under various frequencies (first row), bode plot of the phase under various frequencies (second row), and nyquist plot of the measured results (third row) for the multifunctional hydrogel with Zn as cathode material, and (a) Zn, (b) Ni, (c) Mo and (d) Ag as anode materials


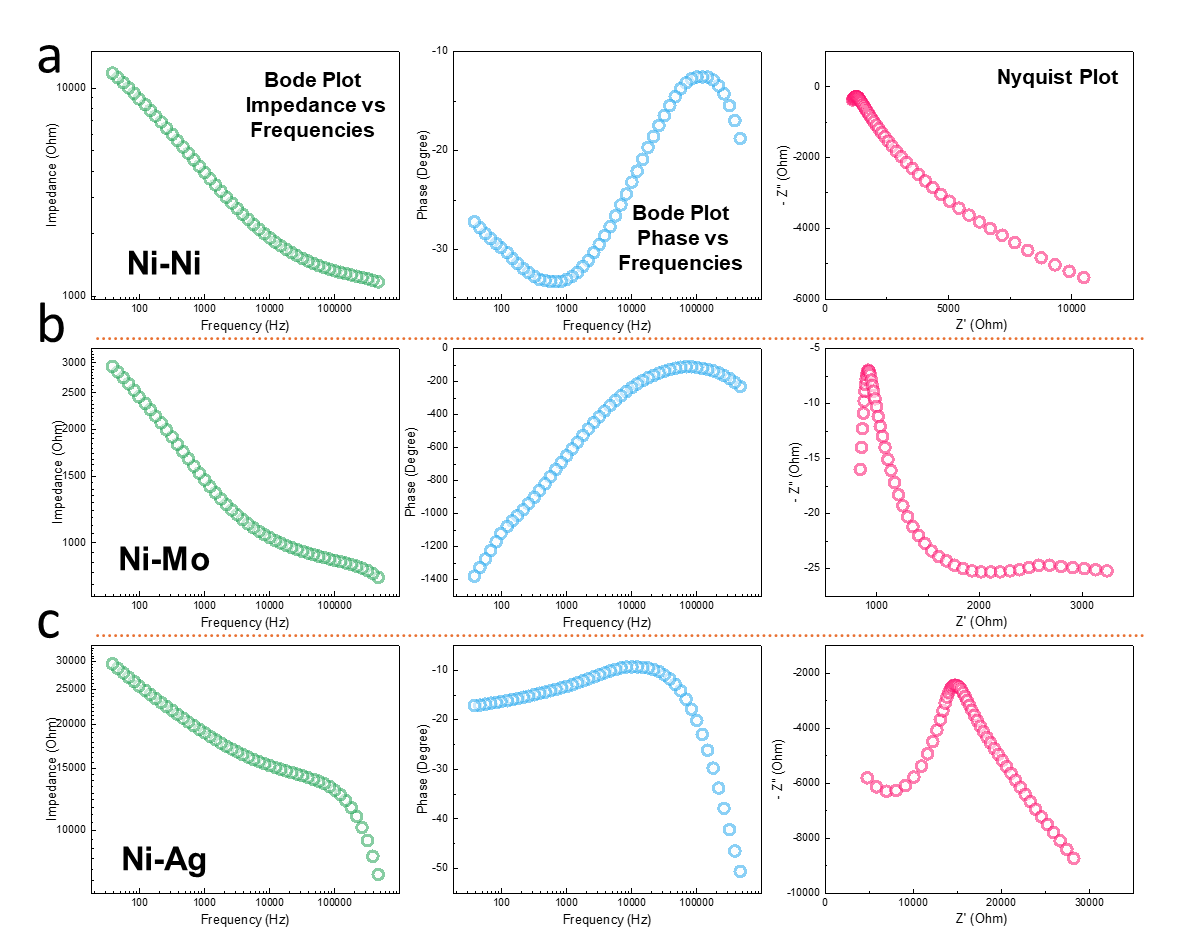


## Fig. S9 Bode plot of the absolute amplitude of the impedance under various frequencies (first row), bode plot of the phase under various frequencies (second row), and Nyquist plot of the measured results (third row) for the multifunctional hydrogel with Ni as cathode material, and (a) Ni, (b) Mo and (c) Ag as anode materials


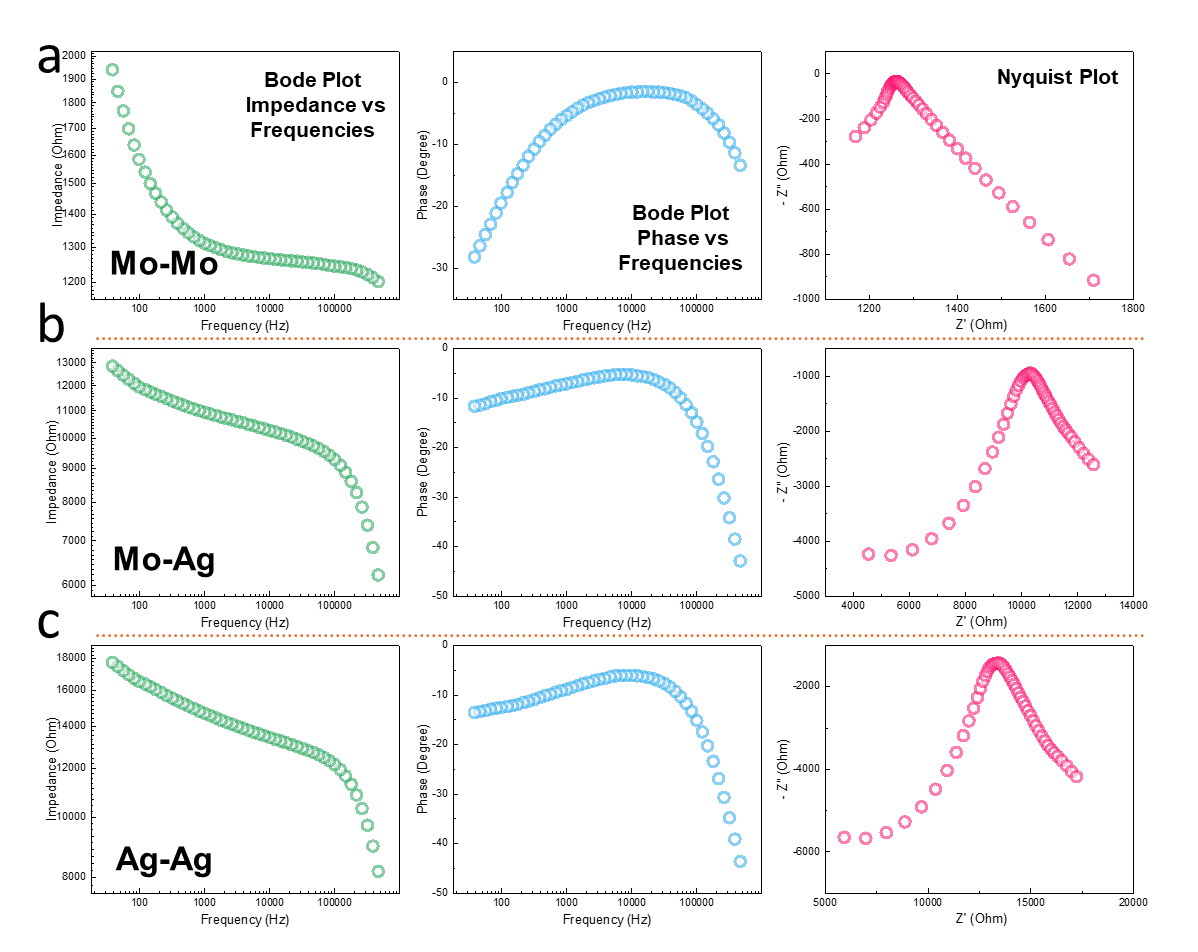


## Fig. S10 Bode plot of the absolute amplitude of the impedance under various frequencies (first row), bode plot of the phase under various frequencies (second row), and Nyquist plot of the measured results (third row) for the multifunctional hydrogel with following metal pairs (cathode - anode): (a) Mo–Mo, (b) Mo-Ag and (c) Ag-Ag

# Note S2 Discussion of the electrochemical model of multifunctional hydrogel with Cu-Al metal pair as electrodes

**Description of Electrical Double-layer (EDL)**

In electrochemical reactions, an electric double-layer (EDL) (as illustrated in **Fig. S11**) arises due to the interaction between ions in the electrolyte and the charged surface of the electrode. The EDL consists of two distinct layers of charge: the inner Stern layer, which includes the specifically adsorbed ions directly on the electrode surface, and the outer diffuse layer, where ions are distributed in a gradient influenced by electrostatic forces.

The inner Stern layer, also known as the compact layer, is tightly bound to the electrode surface and includes both the Outer Helmholtz Plane (OHP) and the Inner Helmholtz Plane (IHP). The OHP is situated slightly farther from the electrode surface, just beyond the IHP, and is composed of ions that are not adsorbed but are held near the electrode by electrostatic forces. The amount of charge in the OHP is matched by the magnitude of counter-charges in the electrode. The IHP is a monolayer that consists of solvated ions or neutral molecules that are strongly bound to the electrode due to specific adsorption and separates the oppositely polarized ions from each other, forming a molecular dielectric at the electrode-electrolyte interface. Therefore, the separated layers of polarized ions in the double-layer store charge similarly to that of a conventional capacitor, creating an electric field within the molecular-scale IHP. The diffuse layer extends from the OHP into the bulk electrolyte, which contains ions that are distributed loosely and associated with the electrode. The ion concentration decreases with increasing distance from the electrode surface, and this layer is relatively more dynamic than the inner Stern layer.

A thinner EDL is often preferred in experiments as it results in a larger electric field intensity and higher capacitance. This enhances the efficiency of charge migration and ion diffusion, which is critical for improving the performance of electrochemical devices. Thinner EDLs can be achieved by using high surface area electrodes, optimizing electrolyte concentration, and operating at conditions that minimize the thickness of the diffuse layer [S1–S3].


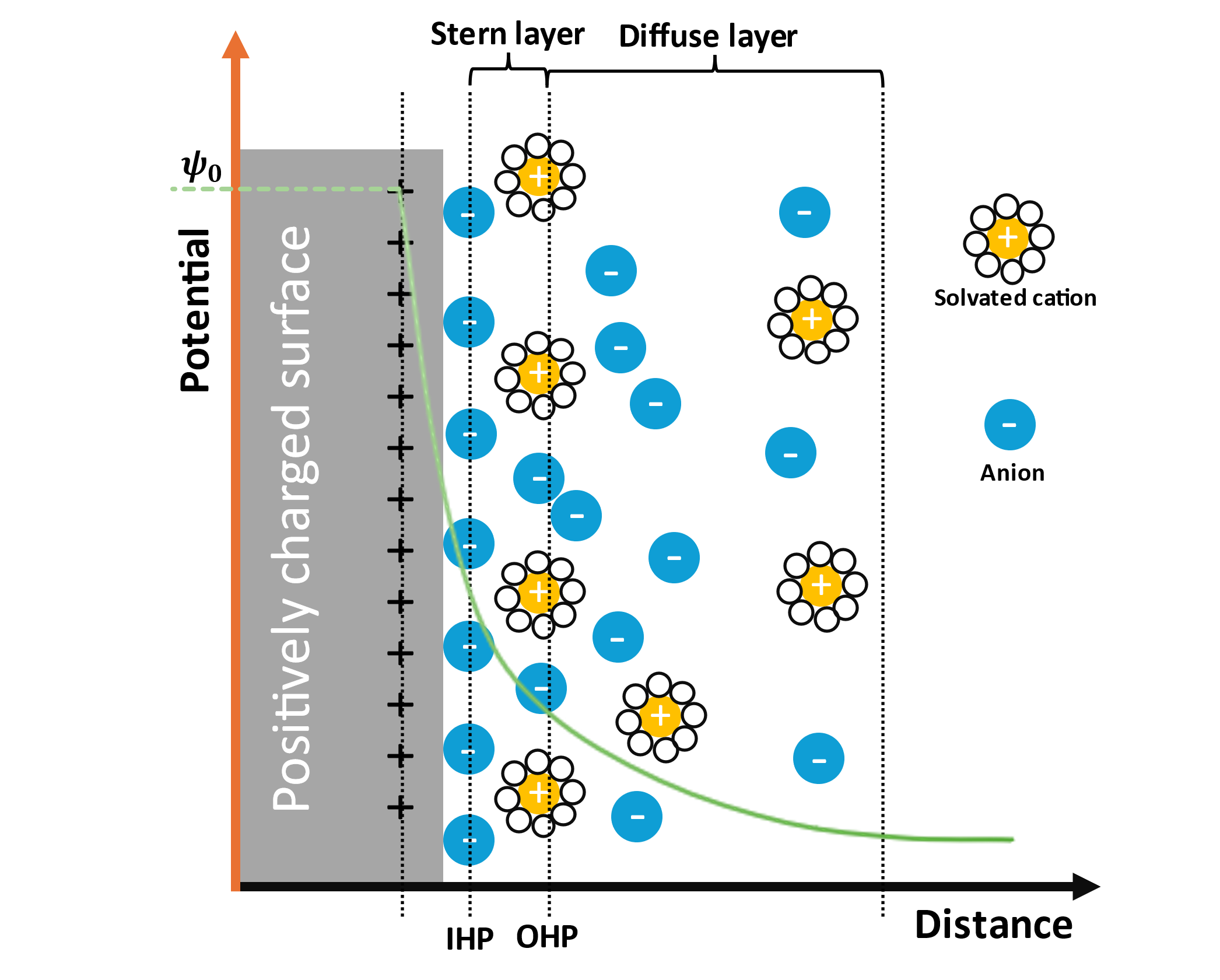


## Fig. S11 Schematic representation of the electrical double layer of the proposed multifunctional hydrogel based on Stern's model

## Description of Faradaic and non-Faradaic process

In electrochemistry and electrochemical engineering, it is crucial to distinguish between two types of processes that can occur in an electrode-electrolyte system: Faradaic and non-Faradaic processes. This distinction is significant because it reflects fundamentally different modes of electrode operation, influencing how these processes can be characterized experimentally.

In Faradaic processes, current or electrons cross the interface between the conducting (metallic) bulk phase and the electrolyte by virtue of an electrochemical reaction such as the reduction or oxidation of water or of some ion if electrons move to the electrolyte, the ions (or other molecules) that enter the electrode in reduced form leave as oxidized species when they return to the electrolyte phase (or alternatively to another bulk phase). If ions cross the electrode, from an electrolyte phase into the bulk metal phase, which supplies the electronic charge, as seen in metal plating processes. (Conversely, atoms can dissolve from the electrode as ions.)

In non-Faradaic processes, charged particles do not cross the interface. Instead, the current is due to the charging and discharging of the aforementioned electrical double-layer at the electrode-electrolyte interface rather than through redox reactions, behaving like a capacitor in series with the Ohmic resistance of the electrolyte. This occurs when there is no actual electrode reaction or when the electrode reaction involves atoms that are part of the electrode structure itself, or when reacting species are oxidized/reduced in the electrode but remain within it, thus truncated in the electrode [S4].

**Description of Constant Phase Element (CPE)**

Here, we use the CPE instead of an ideal capacitor to model the electrical double-layer capacitance typically formed at the solid/electrolyte interface, which universally exhibits nonideal capacitive behavior due to surface roughness and irregularities in surface termination, porosity, inhomogeneities, and complexity in the double-layer structure [5].

The impedance of a CPE is sufficiently defined by the relation:

$$\begin{aligned} Z_{CPE}=\frac{1}{(j\omega)^{\beta}Q}\#\left( S1 \right) \end{aligned}$$

Where:

$Z_{CPE}$ is the actual impedance of the CPE.

$Q$ is the CPE constant containing the nonideal capacitance information, which has units of $F\cdot s^{\beta-1}$ (or equivalently $\left( \Omega\cdot s \right)^{-\beta}$)

$\beta$ is the CPE exponent or ideality factor, which typically ranges between 0 and 1. It characterizes the deviation from ideal capacitive behavior and is related to the angle $\theta$ as $\theta={90}^{\circ}\left( 1-\beta\right)$, where $\theta$ is the phase deviation from the ideal case ($\varphi=90^{\circ}$). If $\beta=1$, $Z_{CPE}= {(j\omega Q)}^{-1}$, the CPE behaves like an ideal capacitor, which can be experimentally obtained only in the case of perfectly flat electrodes, as for example the liquid Hg drop electrode. On the other hand, if $\beta=0$, $Z_{CPE}= Q^{-1}$, the CPE behaves like a pure resistor. If $0<\beta<1$, the CPE represents a nonideal capacitor [S6]. Normally, $\beta$ is found to be in the range of 0.8-1 [S5].

**Description of Warburg diffusion term**

Warburg is a circuit element that represents the diffusion process in the electrochemical system. It accounts for the additional impedance of an electroactive species diffusing to, away from, or through a solid/liquid interface due to the mass transfer limitations.

Herein, the Warburg element is associated with the thin-film hydrogel, indicating the difficulty of mass transport of the redox species to the electrode surface considering a Finite-Length diffusion. For Warburg diffusion in a layer of finite thickness, two scenarios can be distinguished based on the boundary condition at a distance $L$ from the electrode:

Open Boundary (Reflective Boundary): The concentration gradient of the redox species is zero and thus, no charge transfer occurs at boundary $x=L$. This condition implies that the electroactive species are reflected back into the diffusion layer, and there is no flux of species across the boundary. Typical examples of finite diffusion with reflective boundaries include lithium diffusion in thin solid-state oxide films [S7] and the mass transport of charge carriers in redox polymer film-coated electrodes [S8], etc.

Short Boundary (Transmissive Boundary): Charge transfer occurs at boundary $x=L$. This condition allows the electroactive species to pass through the boundary, meaning there is a flux of species across the boundary, facilitating further electrochemical reactions. Typical examples of finite-length linear diffusion with transmissive boundaries include solid oxide [S9] or polymer membrane fuel cells [S10].

Depending on the two different boundaries, there are two corresponding equations for expressing the Warburg impedance:

$$\begin{aligned} Z_{WO}=\frac{\sigma\sqrt{2}}{(j\omega)^{0.5}}coth[\frac{L}{D^{0.5}}(j\omega)^{0.5}]\#\left( S2 \right) \end{aligned}$$

$$\begin{aligned} Z_{WS}=\frac{\sigma\sqrt{2}}{(j\omega)^{0.5}}tanh[\frac{L}{D^{0.5}}(j\omega)^{0.5}]\#\left( S3 \right) \end{aligned}$$

Where:

$L$ is the effective finite thickness of the Nernst diffusion layer.

$D$ is the effective diffusion coefficient of the diffusing species.

$\sigma$ is the Warburg Coefficient (mass transfer coefficient) describing the resistance associated with diffusion as a function of the concentration of charge carriers and their diffusion coefficients, ranging from 0 to 1.

$$\begin{aligned} \sigma=\frac{RT}{n^{2}F^{2}A\sqrt{2}}\left( \frac{1}{C_{O}\sqrt{D_{O}}}+\frac{1}{C_{R}\sqrt{D_{R}}} \right)\#(S4) \end{aligned}$$

In which, n represents the number of electrons involved, $F$ is the Faradays constant, $A$ is the surface area of the electrode, $R$ is the gas constant, $T$ is the temperature, $D_{O}$ is the diffusion coefficient of the oxidant, $D_{R}$ is the diffusion coefficient of the reductant, $C_{O}$ is the concentration of oxidant and $C_{R}$ is the concentration of reductant. Choosing between the above two equations is determined by the charge transfer resistance. Herein, in the proposed hydrogel, because it still can be treated as a conductor for ions to transmit (zero charge transfer resistance), the Warburg diffusion element with short boundaries is applied.

On the other hand, it is obvious that the Warburg impedance is frequency-dependent. At high frequencies, since diffusing reactants avoid moving very far, the Warburg impedance is approaching zero. It could be quantitatively demonstrated based on the function $\lim_{\omega\to\infty}\tanh[\frac{L}{D^{1/2}}(j\omega)^{1/2}]\cong1$, and thus $Z_{WS}\left( \omega\to\infty\right)=0$. In contrast, at low frequencies, the redox species have the force to diffuse, thereby increasing the Warburg resistance. It could also be demonstrated by the function: $\lim_{\omega\to0}\tanh[\frac{L}{D^{1/2}}(j\omega)^{1/2}]\cong\frac{L}{D^{1/2}}(j\omega)^{1/2},$and thus $Z_{WS}\left( \omega\to0 \right)=\frac{\sqrt{2}\sigma L}{\sqrt{D}}$, which represents that it becomes real and frequency-independent [S11, S12].

**Electrochemical Impedance Spectroscopy (EIS) Measurement for Electrical Circuits Modelling**

Electrochemical impedance spectroscopy (EIS) is a sophisticated analytical method that gathers kinetic and mechanistic insights from various electrochemical systems. It finds applications in diverse fields such as corrosion analysis, semiconductor research, energy systems, chemical sensors, biosensors, and noninvasive medical diagnostics. The principle behind EIS involves disturbing a balanced or steady-state electrochemical system with a small sinusoidal AC signal—either in voltage or current—across a broad frequency spectrum. This method records the system's sinusoidal response in terms of current or voltage to this perturbation. EIS operates on the assumption that the electrochemical system is linear and time-invariant, meaning the response is directly proportional to the input and remains consistent over time. This approach allows EIS to function as a "transfer function" technique, where it models the relationship between the input excitation signal (either AC current or voltage, herein is AC voltage) and the corresponding output signal (voltage or current, respectively, herein is AC current) across various frequencies. Thus, the impedance of the whole system can be derived based on the equation in phasor form:

$$\begin{aligned} Z=E/I=Z_{0}\exp\left( ⅈ\phi\right)\#\left( S5 \right) \end{aligned}$$

Where $Z$, $E$, $I$, $\phi$ are impedance, potential, current, and phase shift between $E$ and $I$, respectively. Subsequently, The impedance $Z$ is expressed by both a real and an imaginary component. When these components are graphically represented, with the real part ($Z_{real}$) along the X-axis and the imaginary part ($Z_{imag}$}), typically negative, along the Y-axis, it results in the formation of a "Nyquist Plot." This type of plot is particularly effective for analyzing resistive processes. In the Nyquist plot, each point corresponds to an impedance value at a specific frequency. The impedance itself can be visualized as a vector with magnitude |Z| that extends from the origin to each plotted point. The phase shift (angle) $\phi$, is represented by the angle between this vector and the X-axis, as shown in Fig. 2 a, b. Alternatively, impedance can also be expressed through a "Bode plot," which is prevalent in the engineering field and provides a distinct analysis compared to the Nyquist plot. Bode plots are particularly advantageous for examining capacitive systems like the electric double layer (EDL). These plots feature two logarithmic graphs: one plotting magnitude vs. frequency and another displaying phase vs. frequency, offering a comprehensive view of the impedance behavior over a range of frequencies [S12], as shown in **Fig. 2g**.

Afterward, the detailed electrochemical characteristics of the PVA/PAAm/LiCl/Cly ionic hydrogel-based device with varying combinations of electrode materials can be obtained and plotted in both Bode and Nyquist form through EIS measurement, operated by an electrochemical workstation which is configured to apply sinusoidal AC signal with an amplitude of 0.7 V in a frequency scope from 10 Hz to 0.464 MHz, as illustrated in **Note S1.**

This capability makes EIS superior to other electrochemical methods because it can differentiate and elucidate intrinsic material properties and numerous concurrent electrical, electrochemical, and physical phenomena occurring within a real system. The challenge lies in isolating these processes, as they manifest across a broad spectrum of time scales—from extremely rapid to quite slow. EIS can dissect complex behaviors such as liquid electrolyte resistance, conductivity variations across different materials like solid polymer electrolytes, charging/discharging dynamics at the electrode/electrolyte interfaces where EDL is generated, the influence of electrode surface morphology and electrolyte composition on capacitive behaviors of the EDL, electrode charge-transfer reaction kinetics, etc. Each is characterized by distinct time constants ($\tau$), which indicate the time behavior of each process. The time constant of a process is given by $\tau=RC$, where $R$ is the resistance of a resistor in $\Omega$ and $C$ is the capacitance of a capacitor in farad.

Remarkably, EIS allows for the interpretation of measurements within an electrochemical system through the modeling of an equivalent electrical circuit, which typically comprises standard passive elements like resistors, capacitors, and inductors, along with more complex components referred to as distributed elements. These components are interconnected in various configurations to mirror the electrochemical processes, each characterized by a unique time constant. To facilitate this simulation, most electrochemical analyzers come equipped with software designed to map impedance data onto a modeled circuit. Additionally, specialized software for equivalent circuit modeling, such as Zview and Zplot (Scribner Associates, Inc.), is available to enhance these simulations. In this context, Zview is employed to perform detailed circuit modeling of our devices.

It's crucial to understand that there is no single definitive model circuit for any given impedance spectrum. Therefore, it is vital that each component of the circuit is logically associated with a specific electrochemical process, ensuring that the model remains scientifically coherent and useful^[6]^. Thus, prior to utilizing the hydrogel in practical applications, an electrochemical circuit model is initially established to analyze the inherent properties of the hydrogel as well as the basic characteristics when attached to two electrodes with different materials, as shown in **Fig. 2e**. Modeling of the proposed hydrogel-based device contains three important parts:

1. Two metallic electrode/hydrogel interface: The electrical components framed in orange are connected in series with a resistance $R_{electrode/electrolyte}$ is the simplest circuit, and often employed, which is called a Randles circuit. It could capture the most fundamental description of an electrode surface where both Faradaic (charge transfer resistance, $R_{1}$ and $R_{3}$) and non-Faradaic (nonideal EDL capacitance, $\mathrm{CPE}_{1}$ and $\mathrm{CPE}_{3}$) currents flow through the solid/electrolyte interface. These two elements are represented in parallel to reflect the fact that total current is the sum of the Faradaic and non-Faradaic pathways. Two same models (Randles circuit) are invoked in order to represent two electrode/electrolyte interfaces.
2. The thin-film hydrogel: In this scenario, the thin-film hydrogel can be treated as a thin-film electrolyte solution with significantly increased resistance (hundreds of $K\Omega$ to several $M\Omega$), thus the diffusion of mobile charges in the hydrogel (electrolyte) should also be described, which is often described using the equivalent circuit that is framed in blue along with an extra resistance $R_{electrolyte\_diffusion}$ connected in series. The circuit is a modification to the Randles circuit where a Warburg impedance term is included in series with the charge transfer resistance $R_{2}$.
3. $R_{S}$: The term included in series for denoting external resistance, which accounts for all resistances associated with wires, clips, or other contacts. Compared to the resistance of the hydrogel-based device, which is in the range of hundreds of kOhms to several Mohms, Rs is considered as 0 and ignored in this work.

Following the circuit prototype (Cu/Al electrode pair, universally adopted for all subsequent experiments and IoT applications) established, the parameters of each electrical component are also fitted by Zview based on the EIS data derived before, as illustrated in **Table S2.** $\mathrm{CPE}-T$ and $\mathrm{CPE}-P$ correspond to CPE constant $Q$ and CPE exponent or ideality factor $\beta$, respectively. $W-T=\frac{L^{2}}{D}$. $W-P$ should be set to 0.5 for a Finite-Length Warburg (FLW) corresponding to the square root in equation (S3), but for a Generalized Finite Warburg element (GFW) as an extension of FLW, it should become a continuously varying exponent such that $0<W-P<1$. $W-R=\frac{\sqrt{2}\sigma L}{\sqrt{D}}$, and at very low frequencies, $Z^{'}$ approaches $W-R$ and $Z^{''}$ goes to zero. Thus equation (S2) could be modified as (S3) (Warburg short element), representing the diffusion for the proposed hydrogel-based stable power supply.

**Table S2** Parameters of each electrical element in the EIS model of PVA/PAAm/LiCl/Cly ionic hydrogel-based device with Cu-Al electrode pair

| Element | Value | Unit |
| --- | --- | --- |
| W_R | 5301 | Ω |
| W_T | 0.014195 | s |
| W_P | 0.50364 | Ω⋅s^1/2^ |
| R1 | 280.6 | Ω |
| CPE1_T | 1.0497E-6 | Ω^−1^⋅s ^CPE2_P^ |
| CPE1_P | 0.8906 | Dimensionless |
| R2 | 1040 | Ω |
| CPE2_T | 3.663E-10 | Ω^−1^⋅s ^CPE1_P^ |
| CPE2_P | 0.92751 | Dimensionless |
| R3 | 3918 | Ω |
| CPE3_T | 1.144E-6 | Ω^−1^⋅s ^CPE3_P^ |
| CPE3_P | 0.87489 | Dimensionless |
| Rs | The resistance of wires is considered as 0 in this work due to too small compared to the hydrogel-based device. | |

# Note S3 Supplementary characterization results and durability test for the multifunctional hydrogel as a stable power supply for self-sustainable IoT sensing

In this supplementary note, additional details are provided regarding the testing of power density, thickness optimization, and durability evaluation of the multifunctional hydrogel as a stable power supply, related to the content in **Fig. 3**.

**Evaluating if the external load force will influence hydrogel output performance by modulating contact intimacy**

A concern with the device arises from the use of Cu and Al as contact electrode materials. Despite the electrodes' thickness being less than 100 μm, their limited flexibility may impact the device's output depending on the degree of contact. To demonstrate that the device achieves optimized output stability immediately after fabrication due to design optimizations, hydrogels of different thicknesses (140, 250, 300, 480, and 680 μm) are tested under three contact scenarios: no external force, an external force of 5N, and an external force of 9N. The detailed testing results for them are depicted in **Figs. S12-S16.** For these figures, axis ranges are consistently controlled across these graphs to facilitate comparison. A comparison figure is summarized and provided in **Fig. S17.** It is observed that further increasing the external load to enhance the contact level hardly affects the device's output performance, with the output characteristics primarily depending on its thickness, as discussed in **Fig. 3**.

**Consultation about the potential electrochemical reactions that may occur to support the operation of the multifunctional hydrogel**

Cyclic Voltammetry (CV) is a widely used electrochemical technique to study the redox behavior of compounds and materials. In CV, the potential of a working electrode is swept linearly back and forth between two set values at a fixed scan rate while measuring the current response. The resulting plot of current (I) versus potential (V) is called a cyclic voltammogram. When the potential reaches a value where an electrochemical reaction can occur (either oxidation or reduction), an increase in current is observed. Peaks in the cyclic voltammogram correspond to the potential at which these reactions occur, and the position and shape of the peaks provide insights into the kinetics and thermodynamics of the reactions. An oxidation peak appears when the potential is sufficient to remove electrons from a species (oxidation reaction), while a reduction peak appears when the potential is sufficient to add electrons to a species (reduction reaction) [S13]. As depicted in **Fig. S18**, a dominant peak at 0.4 V indicates that there is a redox-active species in the system that undergoes a reduction reaction at this potential. In our case, by comparing the peak potential with known standard redox potentials based on the basic materials or ingredients of our multifunctional hydrogel, one can infer the nature of the electrochemical reaction, which may dominate the full redox reaction given below:

$$O_{2}+2H_{2}O+4e^{-}\to4OH^{-} E^{0}=+0.4 V$$

This reaction could happen at the air-cathode end, where the water in the electrolyte reacts with oxygen from the air, and the oxygen may be reduced to hydroxide ions [S14].


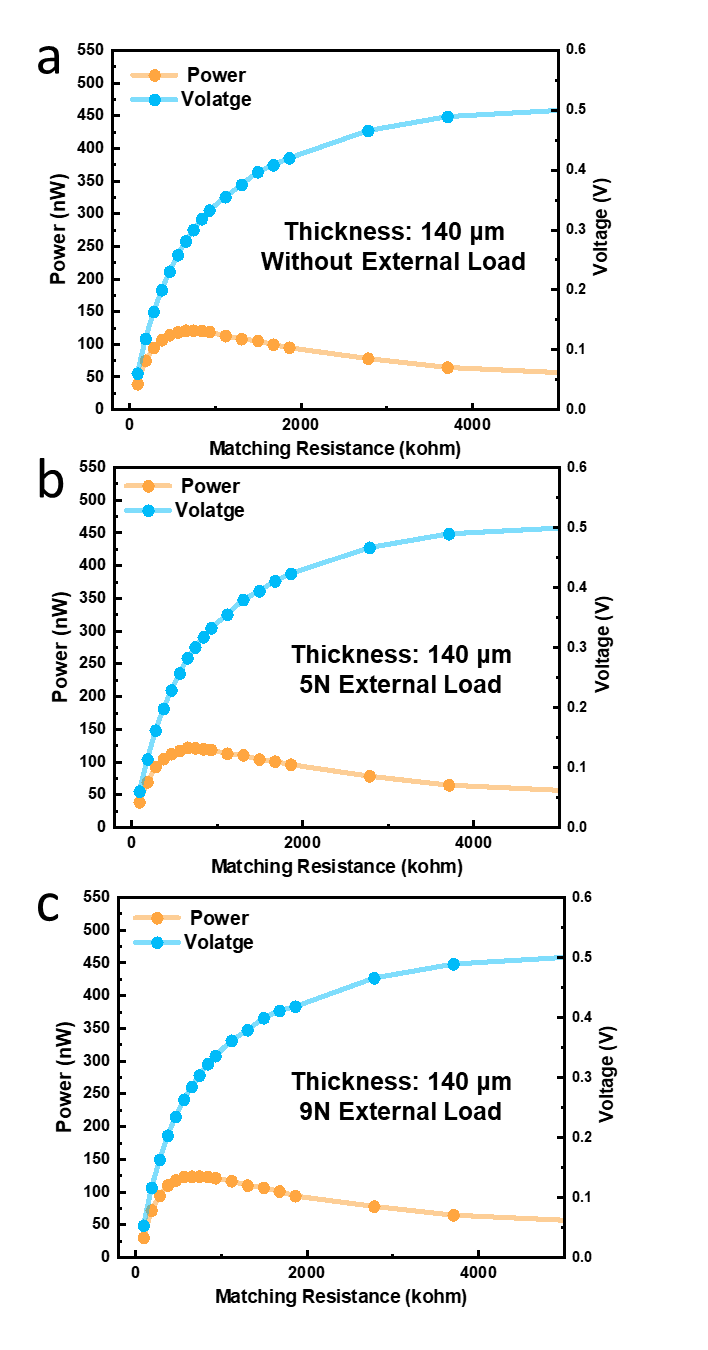


**Fig. S12** Characterization of output performance of the multifunctional hydrogel with 140 μm thickness with different loads

##
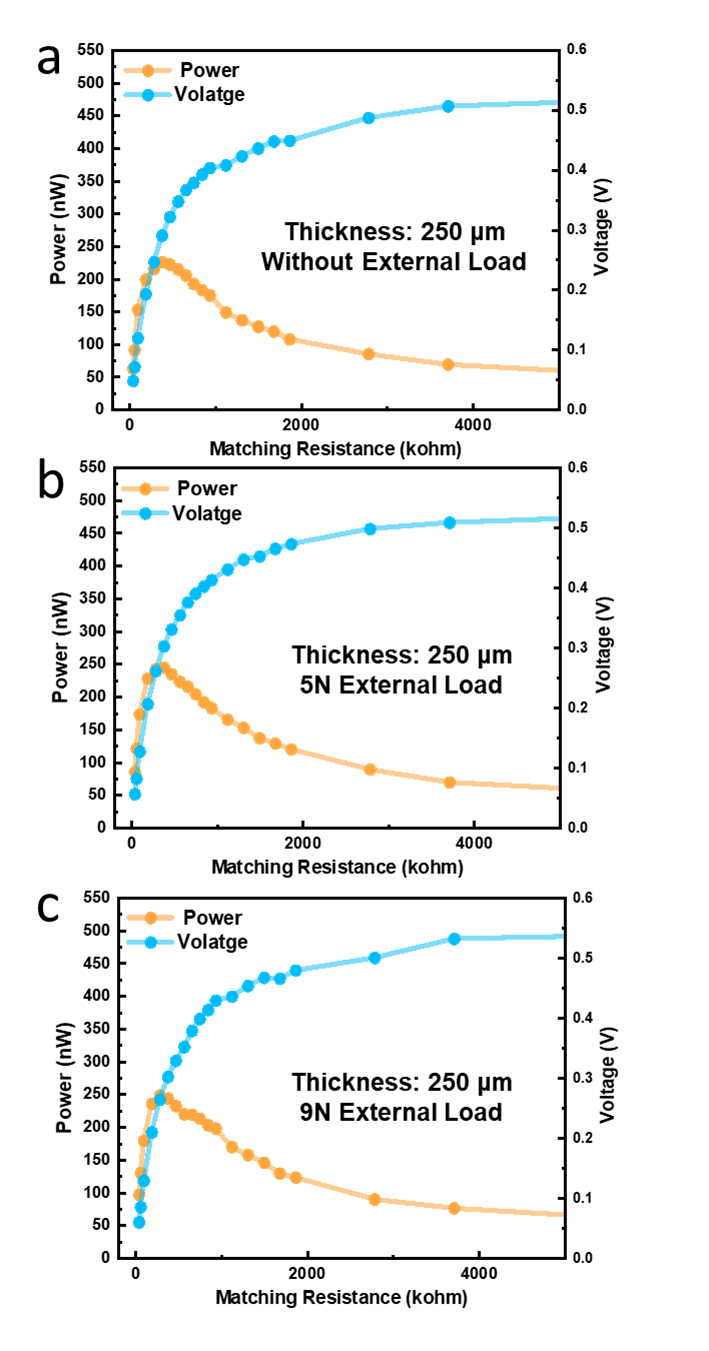


## Fig. S13 Characterization of output performance of the multifunctional hydrogel with 250 μm thickness with different loads


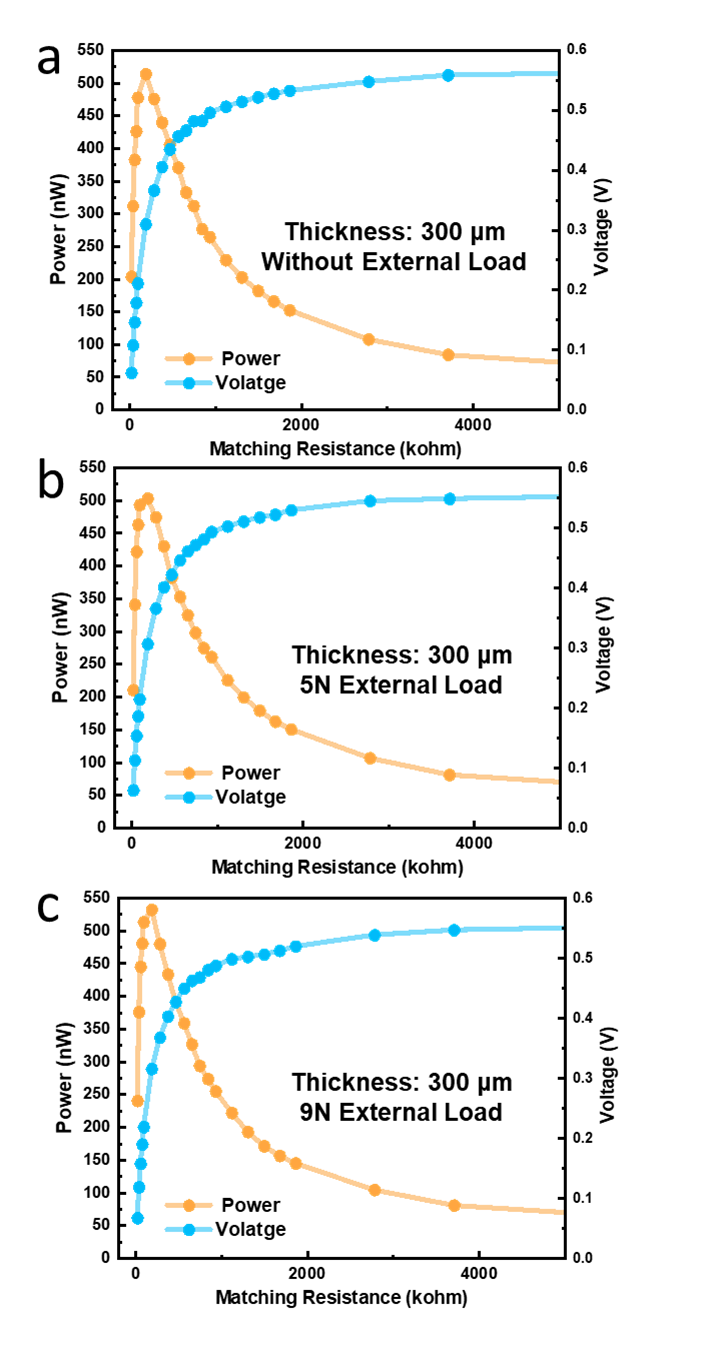


## Fig. S14 Characterization of output performance of the multifunctional hydrogel with 300 μm thickness with different loads


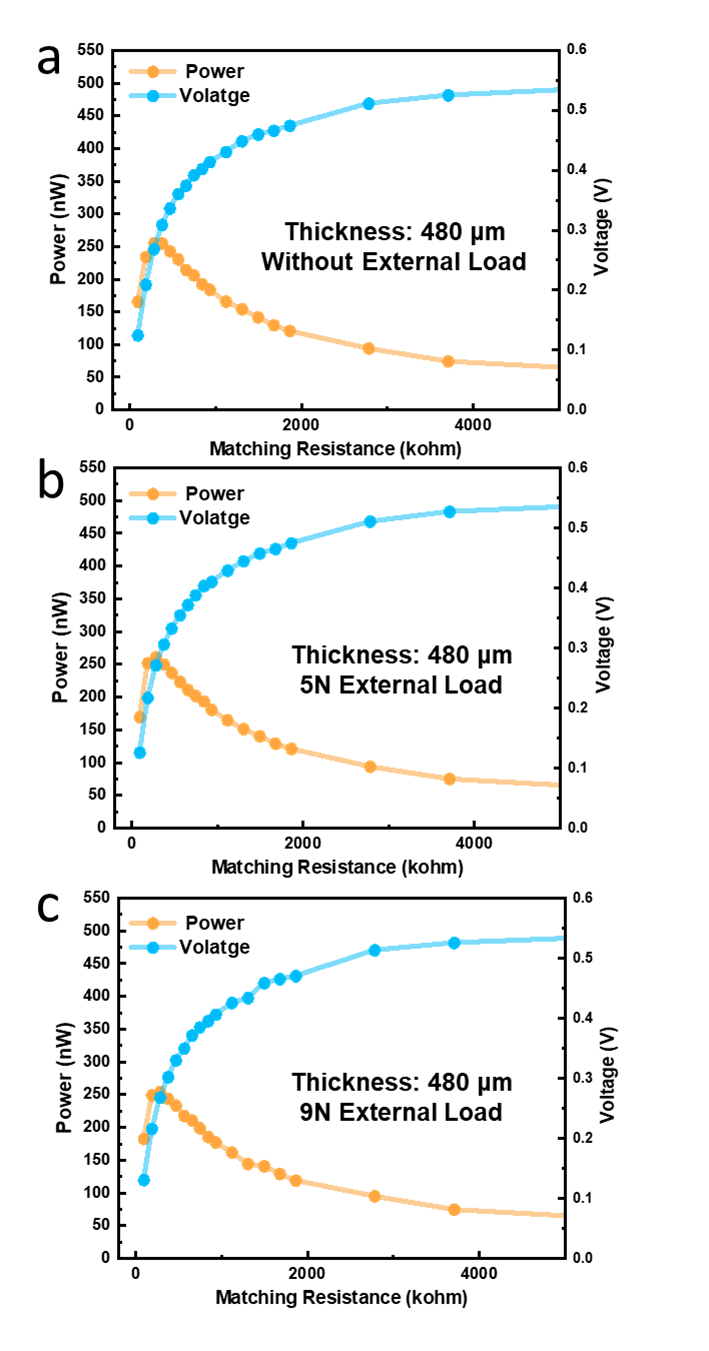


## Fig. S15 Characterization of output performance of the multifunctional hydrogel with 480 μm thickness with different loads


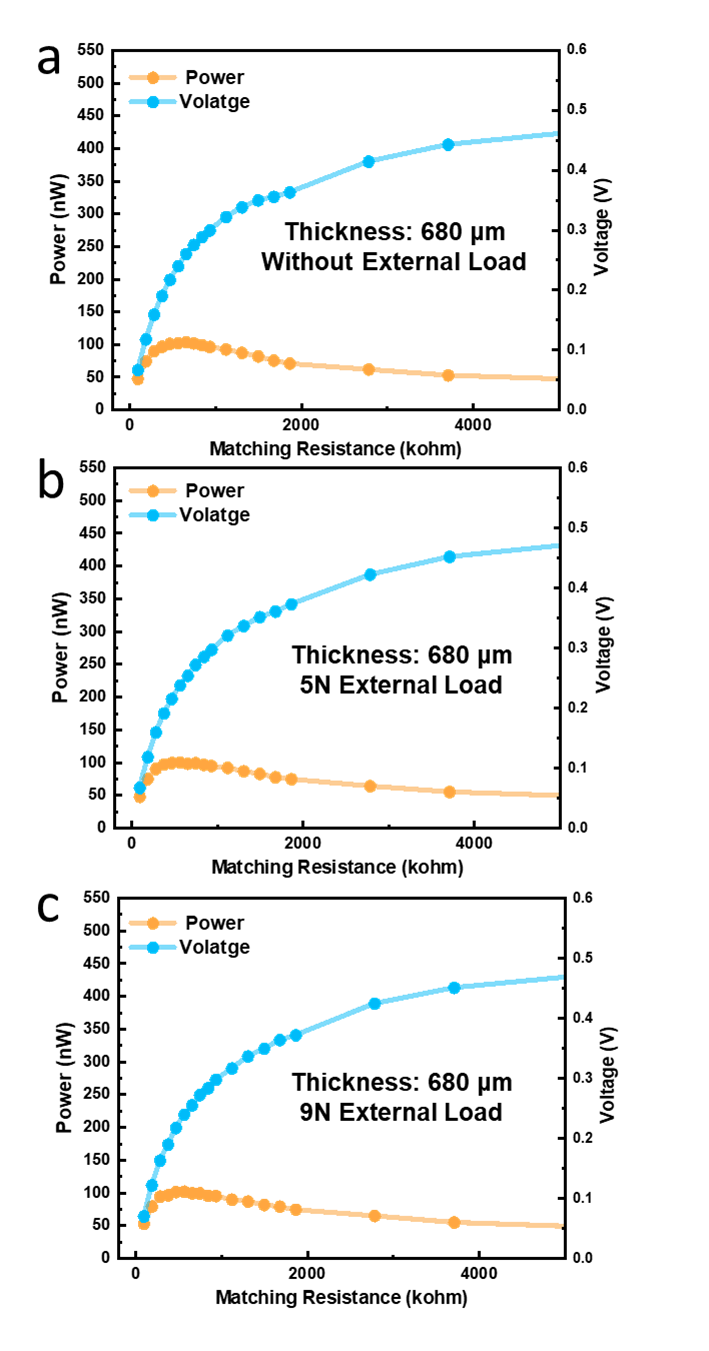


## Fig. S16 Characterization of output performance of the multifunctional hydrogel with 680 μm thickness with different loads


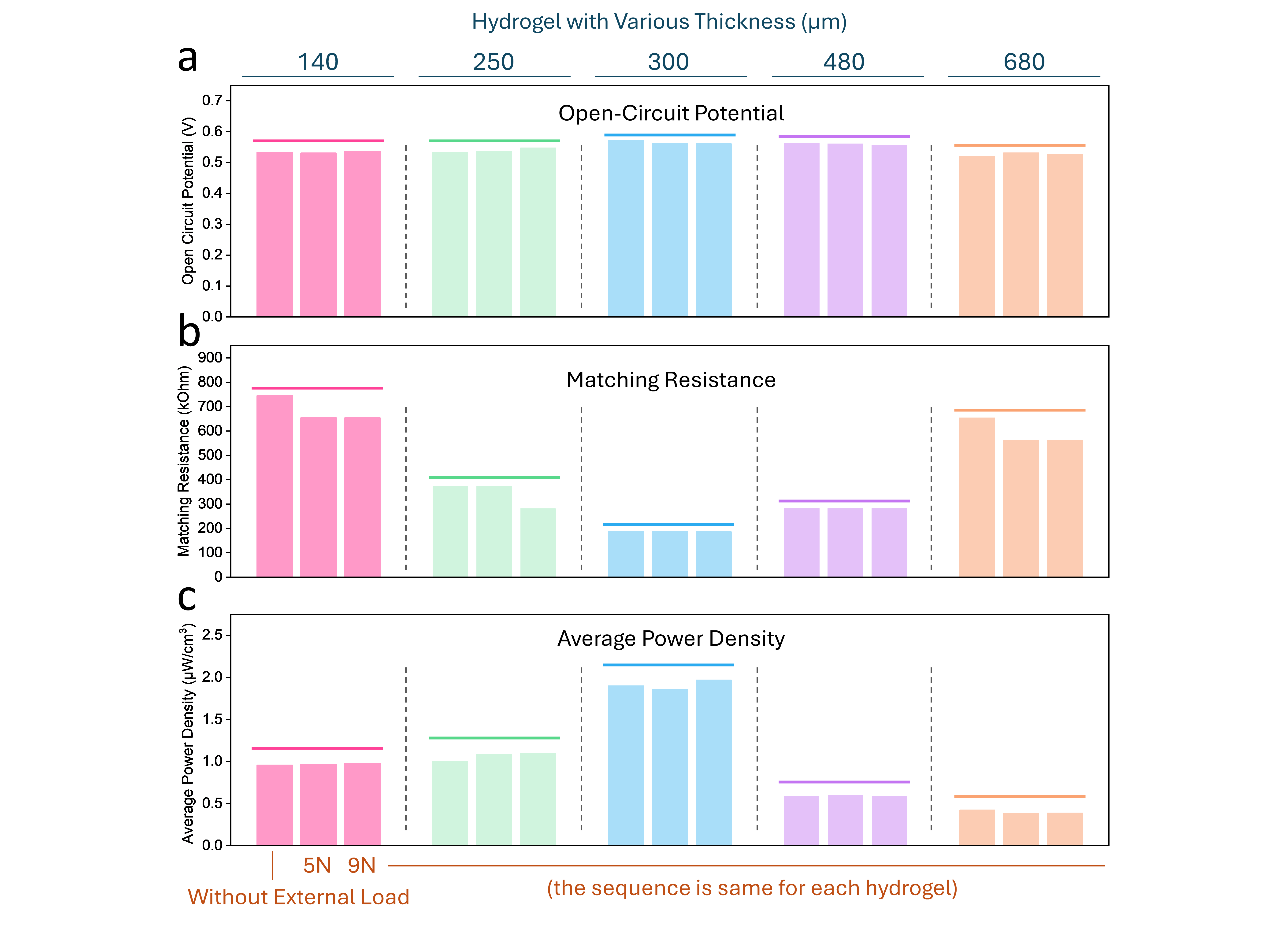


## Fig. S17 Comparison of output performance for multifunctional hydrogels with various thicknesses under different external loads: (a) open-circuit potential, (b) matching resistance, and (c) average power density

**Fig. S18** Cyclic voltammetry (CV) of the multifunctional hydrogel with Cu-Al metal pair as the electrodes at a scanning rate of 0.1V/s for 40 cycles, a pair of redox peaks appears at around 0.4 V


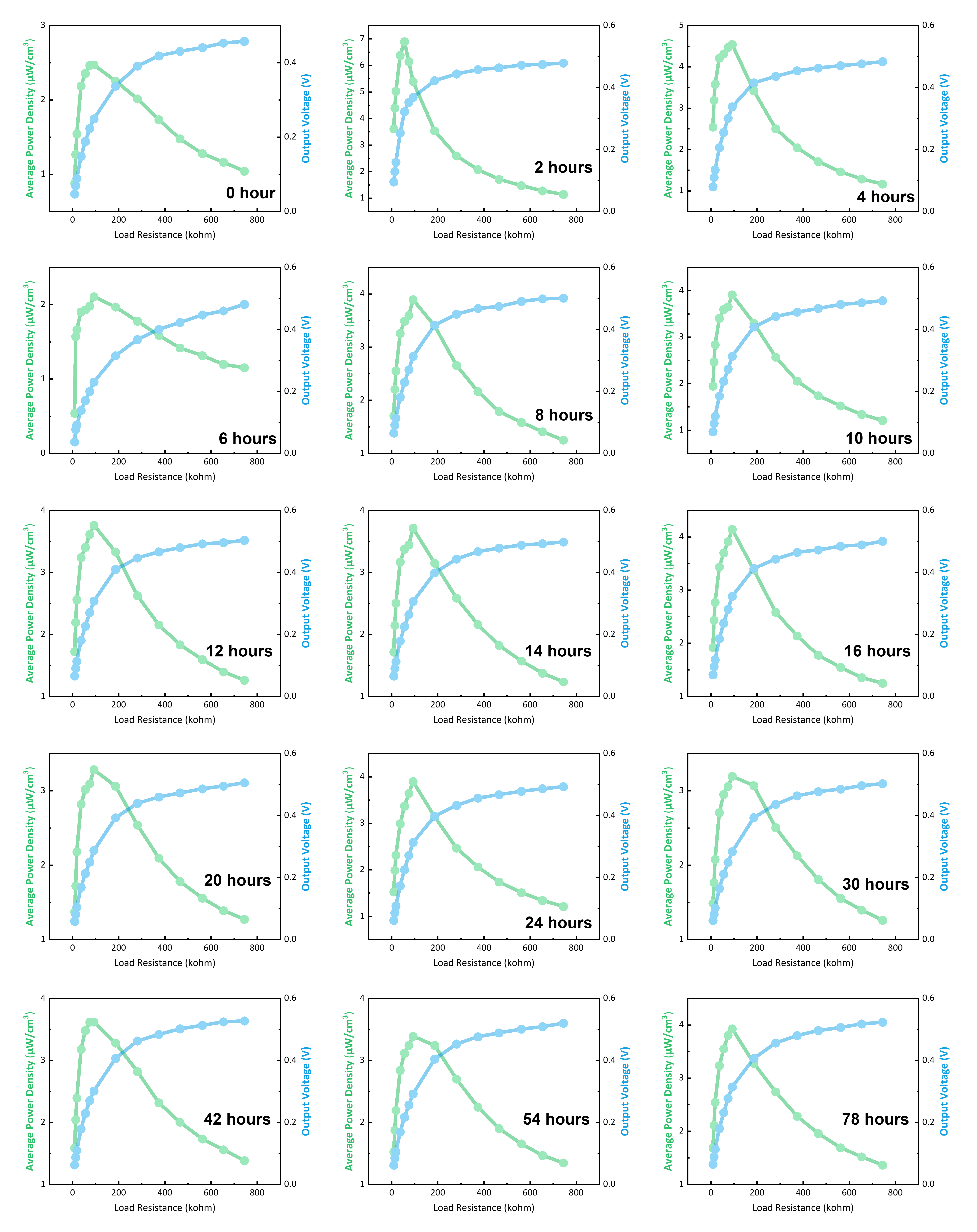


## Fig. S19 Detailed measurements results (0-78 hours) of the durability test for the hydrogel-based EH under normal outdoor environments (24 ℃, 60% RH)


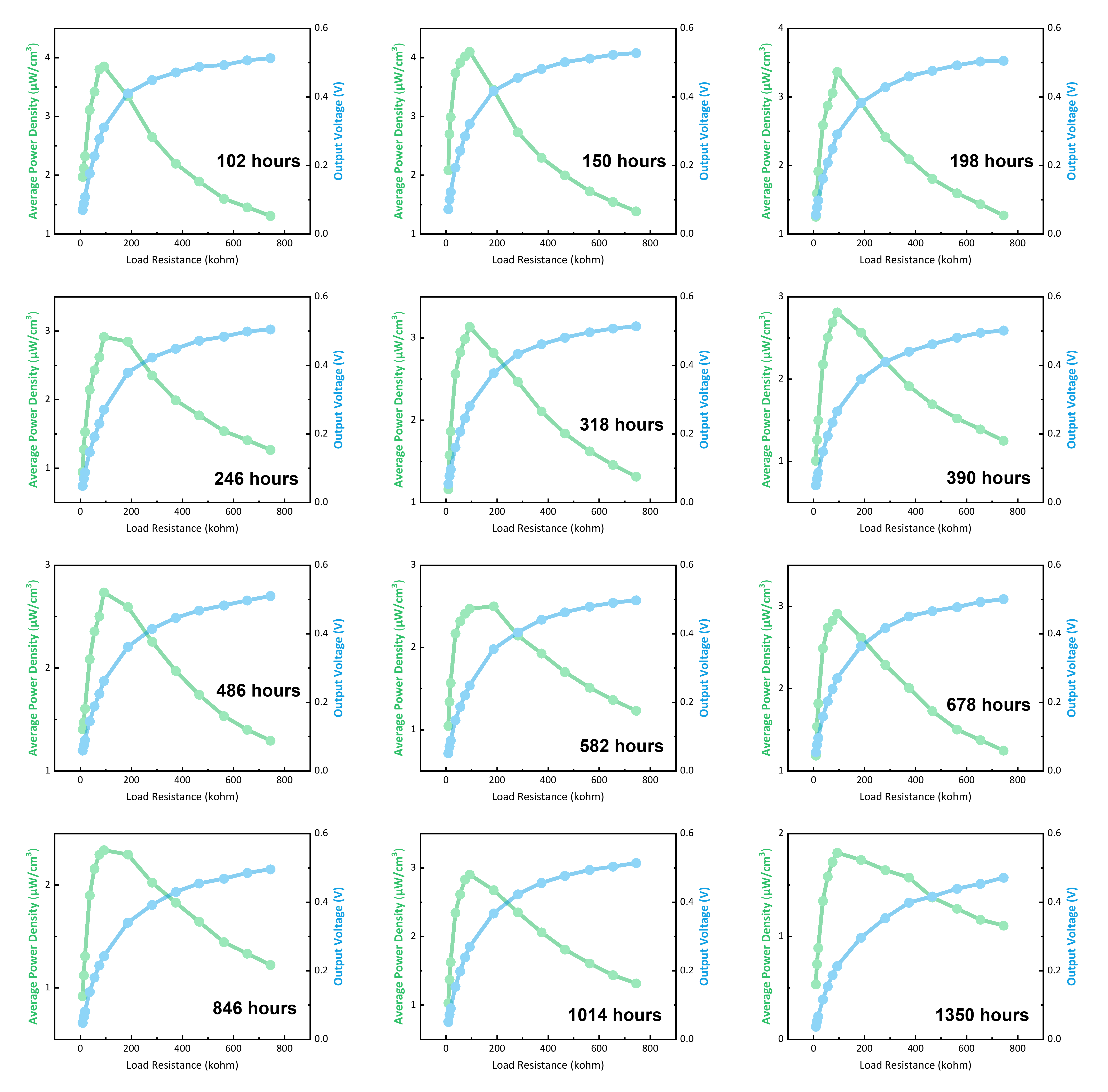


## Fig. S20 Detailed measurements results (102-1350 hours) of the durability test for the hydrogel-based EH under normal outdoor environments (24 ℃, 60% RH)


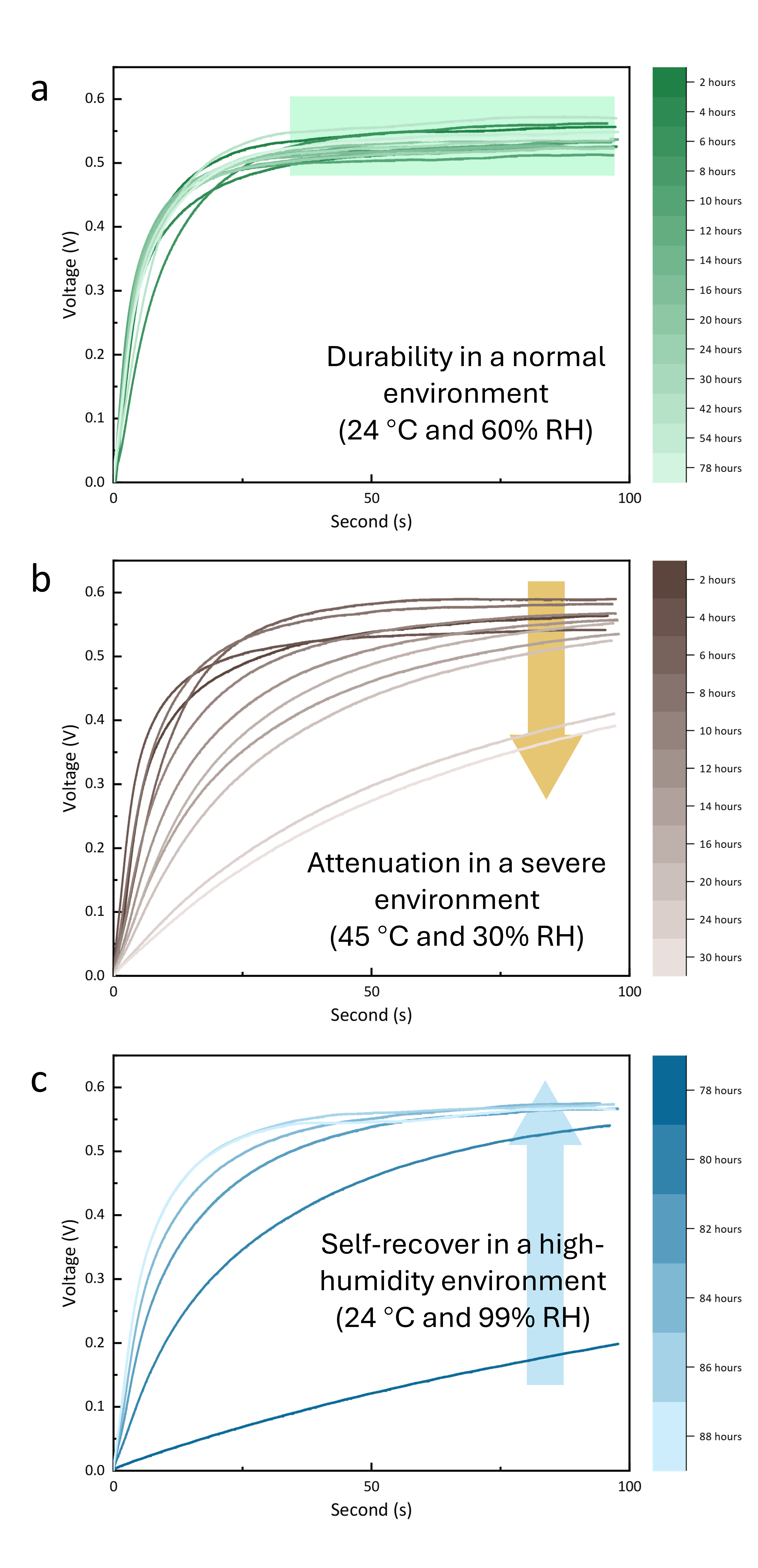


## Fig. S21 Capacitor charging curves of a 100 μF in the durability test of the multifunctional hydrogel as a stable power source with different operation durations: (a) corresponding to the datapoints in Fig. 3i in normal environments; (b) corresponding to the datapoints in Fig. 3k in severe environments; (c) corresponding to the datapoints in Fig. 3l in high-humidity environments;

# Note S4 Electrochemical model and supplementary characterization results for the multifunctional hydrogel as a self-powered noninvasive leaf water content sensor

**Discussion of the modified electrochemical model with introducing the leaf as electrolyte into the sensing system (Fig. S22 and Table S3)**

With regards to the self-powered leaf RWC sensor based on the proposed PVA/PVA/PAAm/LiCl/Cly ionic hydrogel, A widely used method to characterize changes in leaf water content involves viewing the leaf as a high-impedance electrolyte with an electrochemical structure. Through EIS analysis of the leaf, impedance variations at different frequencies can reveal changes in its water content. By leveraging this principle, the leaf can be brought into direct contact with hydrogel, treating it as an electrolyte. Integrating the leaf into the hydrogel's electrochemical model, as described in the paper, enables the detection of changes in leaf water content based on variations in the hydrogel's output. Fig. S23 shows how the hydrogel-based sensor is directly attached to the leaf surface. The hydrogel measures $2 cm \times2 cm$, while the entire device spans $3 cm \times3 cm,$ including the peripheral Aluminum electrode and the substrate. The electrode material remains Cu/Al metallic materials as before, but a notable change is that the aluminum electrode now encircles the hydrogel, avoiding direct contact with it and instead making contact with the leaf surface. This adjustment alters the current electrochemical model compared to the one discussed above:

1. Change in Interface: One of the interfaces has been modified from hydrogel-copper to leaf surface-hydrogel-copper. The leaf's surface, characterized by its more complex microstructures and fewer conductive ions compared to the hydrogel, results in a significant increase in contact resistance. This change can impact the overall performance of the device, as the leaf's unique surface properties introduce new variables into the electrochemical interactions.
2. Alteration of Electrolyte Properties: With the leaf acting as an electrolyte, its higher resistance and poorer ion mobility must be accounted for. The Warburg element, previously considered as a short element in the hydrogel model, now needs to be treated as an open element. This reclassification is necessary to accurately reflect the leaf's influence on the electrochemical behavior of the system.

The EIS measurement results of the updated system with the leaf are compared with the fitting results in **Fig. 4c**. The data show that the proposed model aligns well with the measurement results, demonstrating its accuracy and efficiency. Similarly, the parameters of each electrical element are listed in **Table S3**.

**Measurement of Leaf Relative Water Content (RWC)**

Relative water content (RWC) is a key measure of plant water status, reflecting the physiological impact of cellular water deficit. While water potential is useful for understanding water transport within the soil-plant-atmosphere continuum, it does not account for osmotic adjustment (OA). OA is a crucial mechanism for maintaining cellular hydration under drought stress, and RWC effectively expresses the influence of OA. Therefore, RWC is an appropriate estimate of plant water status, as it considers cellular hydration under the possible effects of both leaf water potential and OA.

The measurement method is straightforward. It estimates the current water content of the sampled leaf tissue relative to its maximum water content at full turgidity, serving as a measure of water deficit in the leaf. Normal RWC values range from 98% in fully turgid and transpiring leaves to about 40% in severely desiccated and dying leaves. In most crop species, the typical RWC at wilting is around 60% to 70%, with some exceptions [S15].

The first step is to sample the top-most fully expanded leaves are sampled, unless interested in profiling leaves on the plant. For large broad leaves such as those of sunflowers and cotton, leaf discs are typically cut to obtain samples of about 5-10 cm². The sample size does not need to be identical for all samples, but it is important to avoid including large veins. Leaf discs should be approximately 1.5 cm in diameter to minimize the cut leaf surface area. Laboratories often design various leaf disc cutters, which may also be available commercially. Alternatively, a sharp cork borer can be used, cutting the leaf over a piece of dense rubber or a large rubber stopper. In contrast, for smaller composite leaves such as groundnuts, common coleus, sea hibiscus, and chickpeas, several leaflets can be combined to quickly and conveniently form a sample. It is crucial that the sampling process be conducted quickly to preserve the integrity of the samples. Meanwhile, samples should reach the lab as soon as possible, which could enlist as much help as possible for the job [S16].

In the laboratory, leaves are first weighed to determine the initial weight or fresh weight of the leaf sample (FW). The samples are then immediately hydrated to full turgidity, immersed in tap water for 4 hours in an environment of 24 ℃ and 60% RH. After soaking, the leaves are gently dried of excess surface moisture and weigh them immediately to obtain the turgid weight (W_T_). At this saturated stage, the RWC is considered 100%. Afterwards, partially dry leaves in an oven at a constant temperature of 50 ℃ to achieve partial dehydration. Take out the samples from the oven and weigh them every 15 minutes to obtain the intermediate weight (W_C_). Repeat the procedure periodically until the leaves weight doesn't decrease, which could be recognized as the dry weight (W_D_). Then we could calculate every intermediate RWC based on equation [S17]:

$$\begin{aligned} \mathrm{RWC}\left( \% \right)=\frac{W_{C}-W_{D}}{W_{T}-W_{D}}\times100\#\left( S6 \right) \end{aligned}$$

For the calibration of the self-powered RWC sensor, an additional process should be conducted after each weighing of partially dried leaves. The hydrogel-based sensor is attached and affixed to the leaf, whose voltage output across the cascaded load resistance (2.52 Mohm) is then measured by the Electrometer (Keithley 6514), and an oscilloscope (Agilent, InfiniiVision, DSO-X 3034A) is used to record the self-generated voltage curve.


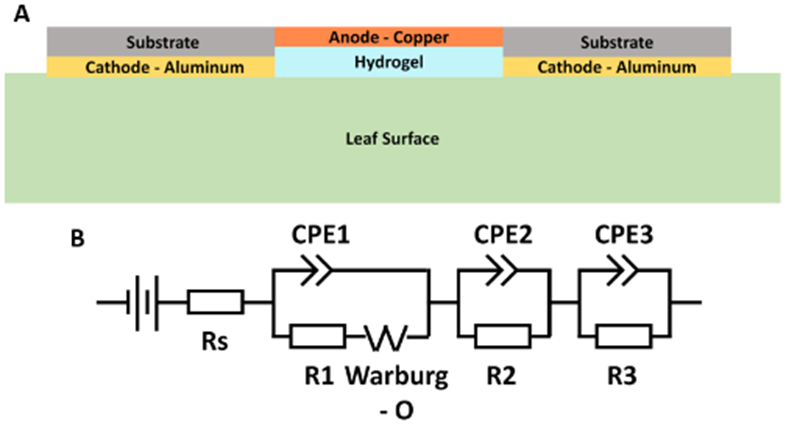


## Fig. S22 Cross-section illustration of the hydrogel-based noninvasively self-powered leaf relative water content sensor attached to the leaf surface

## Table S3 Parameters of each electrical element in the EIS model of PVA/PAAm/LiCl/Cly ionic hydrogel-based device with Cu-Al electrode pair combined with leaves as a noninvasively self-powered leaf relative water content sensor

| Element | Value | Unit |
| --- | --- | --- |
| W_R | 48210 | Ω |
| W_T | 0.00087202 | s |
| W_P | 0.69694 | Ω⋅s^1/2^ |
| CPE1_T | 3.663E-10 | Ω^−1^⋅s ^CPE1_P^ |
| CPE1_P | 0.57291 | Dimensionless |
| R1 | 176580 | Ω |
| R2 | 1.8495E6 | Ω |
| CPE2_T | 2.8379E-8 | Ω^−1^⋅s ^CPE2_P^ |
| CPE2_P | 0.87272 | Dimensionless |
| R3 | 107440 | Ω |
| CPE3_T | 3.751E-10 | Ω^−1^⋅s ^CPE3_P^ |
| CPE3_P | 1.644 | Dimensionless |
| Rs | The resistance of wires is considered as 0 in this work due to too small compared to the hydrogel-based device. | |

## Fig. S23 Photos of the hydrogel-based sensor attached to different leaves to measure the relative water content: (a) Spider Lily (corresponding to Fig. 4d); (b) Sea Hibiscus (corresponding to Fig. 4e); (c) Dry Zone Mahogany (corresponding to Figure 4f) and (d) Common Coleus (Corresponding to Fig. 4g). All the sub-figures have a back scale bar on the bottom of it representing 3 cm length

**Table S4** Comparison of proposed hydrogel-based leaf RWC sensor with other leaf RWC sensors

| **Refs.** | **Year** | **Method** | **Non-Invasive** | **On-Site** | **Large-area Outdoor Monitoring** | **Device Size (cm)** | **Equipment Required**  **for Sensing System** | **System Cost ($)** | **System Power Consumption (W)** | **Resolution** | **Sensitivity** | **Sensing range (RWC, depending on species)** |
| --- | --- | --- | --- | --- | --- | --- | --- | --- | --- | --- | --- | --- |
| [S18] | 2009 | Air-coupled ultrasounds | \ | √ | \ | 30×30 | Ultrasonic transmitter and receiver​, oscilloscope | 950-2700 | 18-65 | 1.5% | 40KHz/% | 97%-100% |
| [S19] | 2012 | Air-coupled ultrasonic resonance | \ | √ | \ | 30×30 | Air-coupled piezoelectric transducers (0.25 MHz, 0.5 MHz, and 1.0 MHz) , oscilloscope​ | 1200-3800 | 18-65 | 1.5% | sigmoid-like, average 2.35MPa/% | 60%-100% |
| [S20] | 2016 | Non-contact Resonant Ultrasonic Spectroscopy | \ | \ | \ | 25×25 | Air-coupled ultrasonic transducers, signal processing electronics, oscilloscope, control software​ | 10000-30000 | 50-150 | 2.0% | 5KHz/% | 70%-100% |
| [S21] | 2019 | Non-contact Resonant Ultrasound Spectroscopy + Deep Learning | \ | \ | \ | 25×25 | Air-coupled ultrasonic transducers (0.15–1.6 MHz), signal processing electronics, CNN | 5000-15000 | 50-150 | 1.9% | 5.1KHz/% | 52%-100% |
| [S22] | 2010 | Air-coupled broadband ultrasonic spectroscopy | \ | \ | \ | 20×20 | Air-coupled piezoelectric transducers (0.3–1.2 MHz), signal processing electronics, oscilloscope | 950-2700 | 50-100 | 1.7% | 5.6KHz/% | 70%-100% |
| [S23] | 2009 | Spectral/optical, Remote sensing (R1300/R1450 - Water Index) | √ | √ | \ | 15×15 | Custom-built sensor with LEDs (1300 nm & 1450 nm) + InGaAs photodiode detector + Reflectance calibration system​ | 460-1400 | 2-8 | 5.0% | 0.0071/% | 40%-100% |
| [S24] | 2020 | Hyperspectral imaging with deep learning (DeepRWC) | √ | \ | √ | 100×100 | VNIR hyperspectral camera, lighting module, conveyor system, high-performance computer with GPU for deep learning model training​ | 18000-73000 | 630-1200 | / | / | 60%-100% |
| [S25] | 2024 | Electrical Impedance Spectroscopy (EIS) | √ | √ | \ | 20×30 | IM3570 impedance analyzer, custom polyurethane foam electrodes for continuous measurement​ | 5050-15200 | 50-200 | 1.0% | / | 89%-93% |
| [S26] | 2011 | Microwave L-band (1730 MHz) | √ | \ | √ | 10×10 | Microwave DCT patch antenna (1730 MHz), signal processing unit | 100-500 | 2-5 | 1.0% | 0.0015/% | 25%-100% |
| [S27] | 2006 | Infrared Thermography | √ | √ | \ | 50×50 | Digital infrared thermography camera (8-12 μm range), software | 5000-20000 | 50-150 | / | / | 50%-100% |
| [S28] | 2015 | Impedance Spectroscopy | √ | √ | \ | 30×30 | ECG electrodes, impedance analyzer (1 kHz–100 kHz) | 5050-15200 | 50-200 | 4.0% | 2.3Kohm/% | 65%-100% |
| [S29] | 2024 | Impedance Spectroscopy | √ | √ | \ | 16×16 (IDC), 16×38 (LC) | Screen-printed IDC/LC ((interdigital capacitor/planar inductorcapacitor sensors)) as E-tattoo, impedance analyzer | 5050-15200 | 150-250 | / | / | / |
| [S30] | 2023 | Impedance Spectroscopy | √ | √ | \ | 16×16 | Liquid Metal Plant Electronic Tattoo (LM-PET), impedance analyzer | 5100-15500 | 50-200 | 4.0% | 25.4Kohm/% | 50%-100% |
| [S31] | 2009 | Impedance Sensing | \ | √ | \ | 16×16 | Needle-type water content sensor with a polyethersulfone (PES, poly(4-phenoxy-co-4-benzensulfone)) membrane, impedance analyzer | 100-500 | / | 1.5% | B=0.036/%, Z0= 3.5×10^6, Z=Z0×10^(-B×pwc%), | 30%-70% (PWC) |
| [S32] | 1988 | Pressure Chamber Method | √ | \ | \ | 25×25 | Pressure chamber, pressure gauge, compressor | 2000-5000 | 50-150 | 4.0% | -26.3KPa/% | 60%-100% |
| **This work** | **2024** | **Impedance matching** | **Non-invasive** | **On-site** | **√** | **3×3** | **Commercial IoT modules** | **0.18 (device) 1-10 (IoT)** | **Self-Sustainable** | **8.0%**  **(average)** | **5.26mV/%**  **(average)** | **20%-100%** |

## Table S5 Surface roughness measurements of four leaves

| Leaf | Ra (μm) | Rz (μm) | Rq (μm) | Rt (μm) |
| --- | --- | --- | --- | --- |
| Spider Lily | 0.95 | 4.55 | 1.27 | 10.04 |
| Sea Hibiscus | 4.55 | 16.97 | 5.31 | 21.10 |
| Dry Zone Mahogany | 3.16 | 16.54 | 4.50 | 27.06 |
| Common Coleus | 2.48 | 12.71 | 3.01 | 16.58 |

Ra: Arithmetic Mean Roughness

Rz: Maximum Height of the Profile

Rq: Root Mean Square Roughness

Rt: Total Height of the Profile

The surface roughness is measured by a surface roughness tester (TR-110, JINCHE).

**Note S5 Cascaded hydrogels to improve the power output and circuit system design for self-sustainable IoT systems**

**Automatic charging/discharging control circuit**

In order to realize the application scenario of the Internet of Things and automation, a circuit is built to control the automatic charge and discharge of the capacitor, as illustrated in **Fig. S25**. When $Q_{1}$ MOSFET is conductive ($Q_{2}$ MOSFET is cut-off), the power source $V_{Hydrogel}$ charges capacitor $C_{3}$. In contrast, when $Q_{1}$ MOSFET is conductive ($Q_{1}$ MOSFET is cut-off), the capacitor discharges to supply power for the IoT system ($R\_Load$).

A power source with output of 15 V is comprised of 26 pieces of hydrogels cascading. Schmitt trigger based on the LM393D low power dual differential comparator (Texas Instruments) is introduced to monitor the potential difference across the 500μF capacitor and switch the output voltage to control the conduction and cut-off of the back-end N-channel MOSFET BSS138 (ONSEMI), so as to control the capacitor's charge and discharge loop.

The voltage divider consisting of $R_{3}$, $R_{4}$, $R_{5}$ sets the high threshold voltage $V_{TH}$ and low threshold voltage $V_{TL}$ of the Schmitt trigger to 3.234V and 2.898V, respectively, which is the maximum and minimum potential difference that can be reached across the capacitor. The threshold voltage of the Schmitt trigger is calculated as follows:

When the LM393D (U5A) outputs high voltage, which is approximately 5V here, the potential at its non-inverse input (+), which is the high threshold voltage at this moment, is calculated by:

$$\frac{5-V_{TH}}{31.87}+\frac{5-V_{TH}}{3.8}=\frac{V_{TH}}{6.2}$$

where $V_{TH}=3.2308 V$. Similarly, when the LM393D outputs low voltage, which is approximately 0V here, the potential at its non- inverse input (+), which is the low threshold voltage at this moment, is calculated by:

$$\frac{V_{TL}}{31.87}+\frac{V_{TL}}{6.2}=\frac{5-V_{TL}}{3.8}$$

As illustrated in Figure 6c, when the capacitor C3's voltage is below 3.234V, the Schmidt trigger outputs a high level (approximately 4.9V), higher than MOSFET Q1's gate threshold voltage $V_{gs\left( th \right)}$(approximately 2V), and the power source $V_{Hydrogel}$ charges the 10mF capacitor through Q1. As for the MOSFET Q3, because its gate is connected with the inverter, whose flip input voltage is set as 3.75V, the input voltage of the gate is extremely low at this time (30mV), so Q3 is in the cut-off region in the meantime.

When the capacitor voltage continues to rise for approximately 182.78 s and exceeds 3.23 V, the output level of the Schmitt trigger switches to a low level, at which point Q1 cut off and Q3 turns on, and the capacitor C3 releases its stored energy to supply power to the IoT system for temperature and humidity measurement, typically 5.31 s.

The temperature and humidity measurement are conducted by commercial module SHT35 (Sensirion AG), whose average power consumption is 4.8 W ~ 11 μW (voltage range, 2.4 ~ 5.5 V, current (average), 2μA). The Schmitt trigger and the inverter encompassing LM393D dual comparator, will consume some power to work properly, whose actual power dissipation in our case are 49.4 and 27.5μW, respectively. The three parts of elements, consuming approximately 88μW of peak power, are powered by the hydrogel-based power source. The LM393D is designed for low-power applications and offers a wide supply voltage range (2 to 36 V). LM393D is specifically dedicated for comparing two input voltages, making them more suitable for constructing a Schmitt trigger circuit than an operational amplifier (op-amp, like the LM358N). Moreover, its open-collector output stage allows for greater flexibility in designing a Schmitt trigger circuit. On the other hand, BSS138 N-channel MOSFET is utilized to construct the charging and discharging circuit. This MOSFET has a $V_{\mathrm{gs}\left( \mathrm{th} \right)}$ of around 1.5V to 2.5V and can handle continuous drain current up to 200 mA. It operates in enhancement mode, which means that it will turn on when the gate-to-source voltage exceeds the threshold voltage.

**Design of Self-developed Low-power Arduino PCB Board for Analog-to-digital Converting (ADC) and Wireless Communication based on Bluetooth Low Energy (BLE)**

A small printed circuit board (PCB) has been meticulously designed with dimensions of 39.5 mm by 21.1 mm, making it compact yet highly functional. This PCB is specifically dedicated to handling the analog-to-digital conversion (ADC) processing for various sensors, ensuring accurate and efficient data capture. Additionally, it incorporates wireless communication capabilities based on Bluetooth Low Energy (BLE), enabling seamless and energy-efficient data transmission. This integration of ADC processing and BLE communication on a single, small PCB makes it ideal for use in compact, sensor-driven applications, particularly in the realm of the Internet of Things (IoT).

The microcontroller unit (MCU) used in this design is the NINA-B306. The NINA-B306 is a powerful and versatile module designed for Bluetooth 5.0 Low Energy (BLE) applications. It is built on the Nordic Semiconductor nRF52840 chip, which features a 32-bit ARM Cortex-M4 CPU with floating-point unit, 1 MB of flash memory, and 256 KB of RAM. The NINA-B306 is specifically designed for IoT applications, providing robust wireless communication capabilities, high processing power, and low energy consumption (typically ~16mW when in Bluetooth LE mode, Active TX @ 0 dBm). Its integrated antenna and advanced security features make it a reliable choice for secure and efficient wireless communication in a wide range of IoT devices.

For the ADC processing, the sensor's signal is first boosted by two LM324D quad operational amplifiers (op-amp), providing a magnification scale of approximately 5 times. The LM324D is known for its versatility and efficiency, consisting of four independent, high-gain, internal frequency-compensated op-amps. With two LM324D chips, we have the capability to simultaneously amplify 8 channels of analog signal, greatly enhancing the board's capacity to process multiple sensor inputs concurrently. The LM324D operates on a low power supply (3.3 V_DC_), making it particularly suitable for IoT applications where energy efficiency is crucial. Its ability to function with a single power supply and its wide range of operating voltages enhance its compatibility with various sensors and microcontroller units commonly used in IoT devices. The LM324D's low power consumption (~3 mW when all four op-amps are actively working, typical power supply current is 0.7 mA when V_CC_ = 5.0 V) ensures that the overall power budget of the IoT system is kept to a minimum, thereby prolonging the operational time of the system and enhancing the reliability of the IoT device in remote or low-maintenance applications.

The whole system is powered by either a hydrogel-based power source or directly through USB. The supply voltage is initially adjusted down to 3.3 V through a buck converter TPS62840DLCR (Texas Instruments) to power the PCB board. This converter could efficiently step down the input voltage to the required 3.3 V, ensuring stable and reliable power delivery. it is known for its high efficiency (around 90-95% at moderate loads, e.g., tens of milliamps), especially at low power levels which are common in IoT applications. Considering practical application scenarios, the whole system consumes about 30 mW, accounting for power dissipation and other real-world factors. This design ensures the system's energy efficiency and operational longevity, making it well-suited for various IoT applications.

## Fig. S24 Charging curve of 1 mF capacitor by cascaded multiple pieces of hydrogel devices


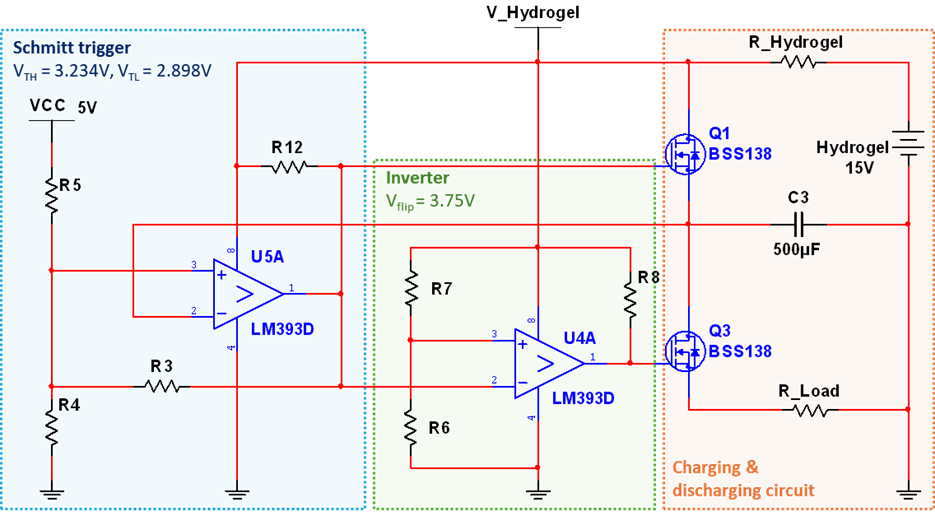


## Fig. S25 Circuit diagram for controlling the automatic charge and discharge of the capacitor in Internet of Things and automation application scenario


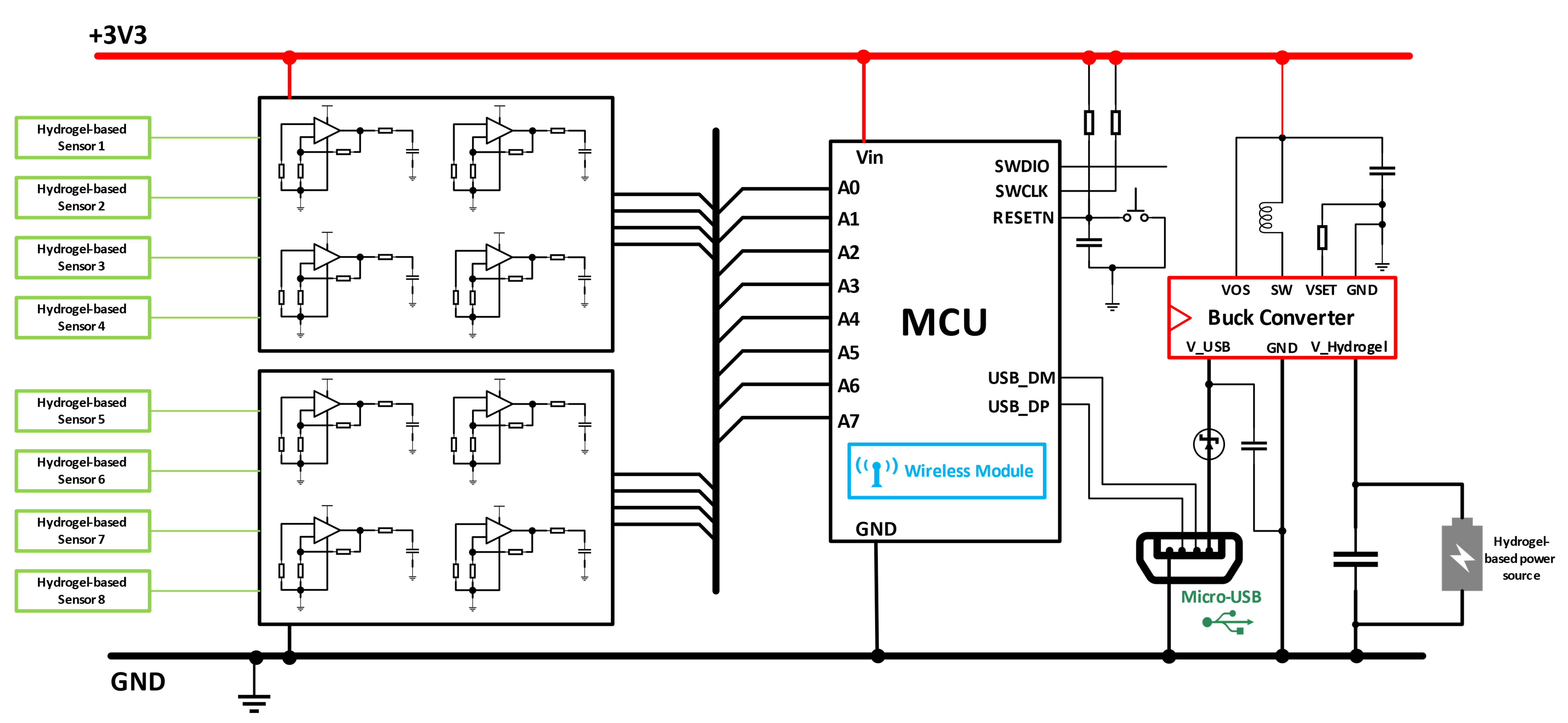


## Fig. S26 Circuit diagram for the designed low-power MCU to collect 8-channel outputs from multifunctional hydrogels and send the information wirelessly with Bluetooth

# Note S6 Discussion of the biocompatibility of hydrogel and fabrication cost

**Polyvinyl Alcohol (PVA)**

Polyvinyl alcohol is extensively studied and utilized across medical applications, attributed to its superior biocompatibility and inherent non-toxicity. It is predominantly implemented in systems for drug delivery and wound care, necessitating direct interaction with biological tissues [S33].

**Glycerol (Gly)**

Glycerol is acknowledged for its efficacy as a benign plasticizer and humectant, playing a crucial role in pharmaceutical and cosmetic formulations. Its capacity to preserve moisture and its non-toxic characteristics are fundamental to its biocompatible profile [S34].

**Lithium Chloride (LiCl)**

Lithium chloride, though minimally used in hydrogels, enhances the conductivity of these materials due to its ionic properties, without undermining biocompatibility. Ongoing investigations into its therapeutic potential support its controlled application in minimal quantities as safe [S35].

**Acrylamide (AAm), N, N'-Methylenebis-acrylamide (MBA) and Ammonium Persulfate (APS)**

The monomeric form of acrylamide presents neurotoxicity; however, when polymerized into polyacrylamide hydrogels, it becomes inert and stable. This stability is vital, as it prevents the leaching of compounds and interaction with the surrounding environment, which is essential for biocompatible sensor applications. The MBA and APS compounds are integral to the polymerization process, serving as the crosslinker and initiator, respectively. Post-reaction, they contribute to forming a stable, non-degrading polymer network that does not leach, thereby maintaining environmental safety [S36].

**3D-Printing**

Molds produced from PLA (Polylite) via 3D printing are non-toxic and extensively used in diverse fields, including medicine. The manufacturing process eschews toxic solvents and adverse conditions, safeguarding the hydrogel from contamination.


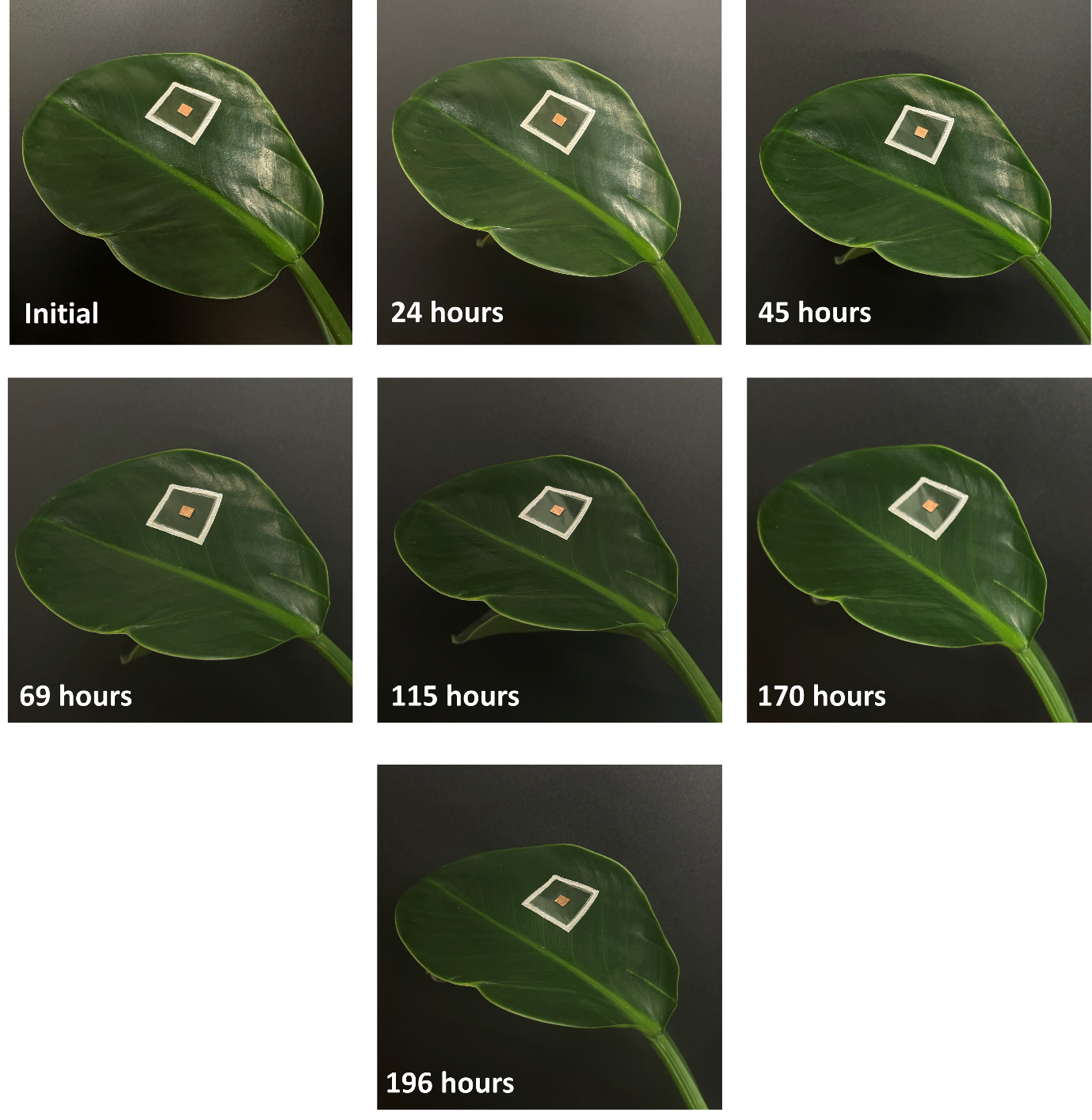


## Fig. S27 Biocompatibility test of the fabricated hydrogel-based leaf RWC sensor attaching to the leaf for more than one week

**Table S6** Cost for fabrication of single hydrogel-based leaf RWC sensor


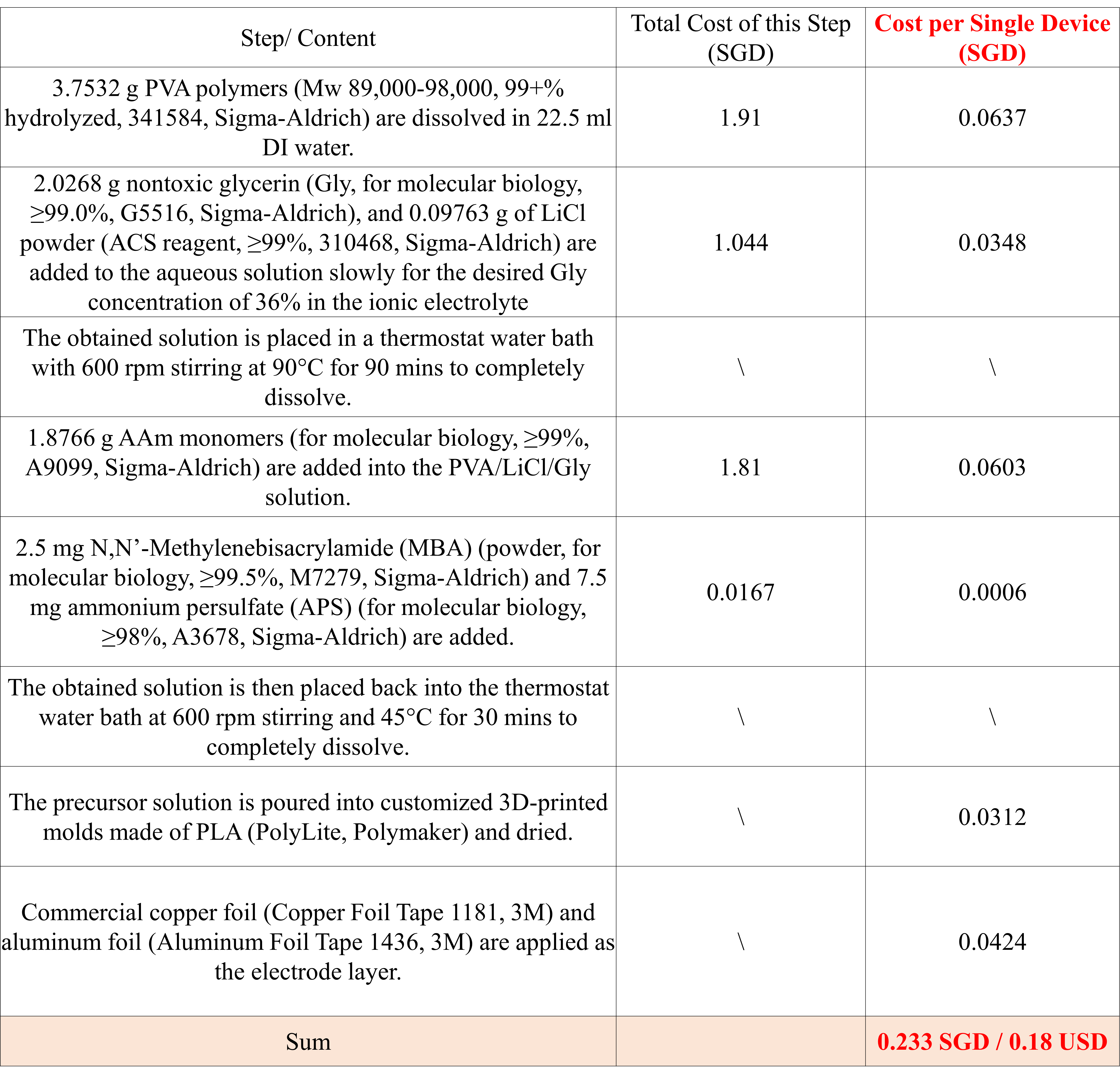


# Supplementary References

1. Q. Wu, C. Dai, F. Meng, Y. Jiao, Z.J. Xu, Potential and electric double-layer effect in electrocatalytic urea synthesis. Nat. Commun. **15**, 1095 (2024). <https://doi.org/10.1038/s41467-024-45522-6>
2. J. Wu, Understanding the electric double-layer structure, capacitance, and charging dynamics. Chem. Rev. **122**, 10821–10859 (2022). <https://doi.org/10.1021/acs.chemrev.2c00097>
3. F. Meder, I. Must, A. Sadeghi, A. Mondini, C. Filippeschi et al., Energy Conversion at the Cuticle of Living Plants. Adv. Funct. Mater. **28**, 1806689 (2018) <https://doi.org/10.1002/adfm.201806689>
4. P.M. Biesheuvel, S. Porada, J. Dykstra, The difference between Faradaic and non-Faradaic electrode processes. (2018).
5. A.R.C. Bredar, A.L. Chown, A.R. Burton, B.H. Farnum, Electrochemical impedance spectroscopy of metal oxide electrodes for energy applications. ACS Appl. Energy Mater. **3**, 66–98 (2020). <https://doi.org/10.1021/acsaem.9b01965>
6. A.C. Lazanas, M.I. Prodromidis, Electrochemical impedance Spectroscopy─A tutorial. ACS Meas. Sci. Au **3**, 162–193 (2023). <https://doi.org/10.1021/acsmeasuresciau.2c00070>
7. S.-I. Pyun, J.-S. Bae, The ac impedance study of electrochemical lithium intercalation into porous vanadium oxide electrode. Electrochim. Acta **41**, 919–925 (1996). <https://doi.org/10.1016/0013-4686(95)00386-x>
8. T.B. Hunter, P.S.Tyler, W.H. Smyrl, H.S. White, Impedance analysis of poly(vinylferrocene) films: the dependence of diffusional charge transport and exchange current density on polymer oxidation state. J. Electrochem. Soci. **134**, 2198–204 (1987). <https://doi.org/10.1149/1.2100851>
9. J.-S. Kim, S.-I. Pyun, Kinetics of oxygen reduction at composite electrodes with controlled three-phase boundaries by patterning YSZ column. Electrochim. Acta **54**, 952–960 (2009). <https://doi.org/10.1016/j.electacta.2008.08.037>
10. S.-J. Lee, S.-I. Pyun, Effect of annealing temperature on mixed proton transport and charge transfer-controlled oxygen reduction in gas diffusion electrode. Electrochim. Acta **52**, 6525–6533 (2007). <https://doi.org/10.1016/j.electacta.2007.04.081>
11. A. Lasia, *Electrochemical Impedance Spectroscopy and its Applications*. pp. 143–248. (1999).
12. H.S. Magar, R.Y.A. Hassan, A. Mulchandani, Electrochemical impedance spectroscopy (EIS): principles, construction, and biosensing applications. Sensors (Basel) **21**, 6578 (2021). <https://doi.org/10.3390/s21196578>
13. K.J. Lee, N. Elgrishi, B. Kandemir, J.L. Dempsey, Electrochemical and spectroscopic methods for evaluating molecular electrocatalysts. Nat. Rev. Chem. **1**, 0039 (2017). <https://doi.org/10.1038/s41570-017-0039>
14. A.A. Mohamad, Electrochemical properties of aluminum anodes in gel electrolyte-based aluminum-air batteries. Corros. Sci. **50**, 3475–3479 (2008). <https://doi.org/10.1016/j.corsci.2008.09.001>
15. H.D. Barrs, P.E. Weatherley, A re-examination of the relative turgidity technique for estimating water deficits in leaves. Aust. Jnl. Bio. Sci. **15**, 413 (1962). <https://doi.org/10.1071/bi9620413>
16. D. Soltys-Kalina, J. Plich, D. Strzelczyk-Żyta, J. Śliwka, W. Marczewski, The effect of drought stress on the leaf relative water content and *Tuber* yield of a half-sib family of 'Katahdin'-derived potato cultivars. Breed. Sci. **66**, 328–331 (2016). <https://doi.org/10.1270/jsbbs.66.328>
17. M. Pieczynski, W. Marczewski, J. Hennig, J. Dolata, D. Bielewicz et al., Down-regulation of CBP80 gene expression as a strategy to engineer a drought-tolerant potato. Plant Biotechnol. J. **11**, 459–469 (2013). <https://doi.org/10.1111/pbi.12032>
18. T.G. Álvarez-Arenas, D. Sancho-Knapik, J.J. Peguero-Pina, E. Gil-Pelegrín Determination of plant leaves water status using air-coupled ultrasounds. 2009 IEEE International Ultrasonics Symposium. September 20-23, 2009, Rome, Italy. IEEE, (2009)., 771–774.
19. D. Sancho-Knapik, H. Calas, J.J. Peguero-Pina, A. Ramos Fernandez, E. Gil-Pelegrin et al., Air-coupled ultrasonic resonant spectroscopy for the study of the relationship between plant leaves' elasticity and their water content. IEEE Trans. Ultrason. Ferroelectr. Freq. Contr. **59**, 319–325 (2012). <https://doi.org/10.1109/TUFFC.2012.2194>
20. T. Gómez Álvarez-Arenas, E. Gil-Pelegrin, J. Ealo Cuello, M.D. Fariñas, D. Sancho-Knapik et al., Ultrasonic sensing of plant water needs for agriculture. Sensors (Basel, Switzerland) **16**, 1–20. (2016) <https://doi.org/10.3390/s16071089>
21. M.D. Fariñas, D. Jimenez-Carretero, D. Sancho-Knapik, J.J. Peguero-Pina, E. Gil-Pelegrín et al., Instantaneous and non-destructive relative water content estimation from deep learning applied to resonant ultrasonic spectra of plant leaves. Plant Methods **15**, 1–10. (2019) <https://doi.org/10.1186/s13007-019-0511-z>
22. D. Sancho-Knapik, T. Gómez Álvarez-Arenas, J.J. Peguero-Pina, E. Gil-Pelegrín, Air-coupled broadband ultrasonic spectroscopy as a new non-invasive and non-contact method for the determination of leaf water status. J. Experimental Botany **61**, 1385–1391 (2010) <https://doi.org/10.1093/jxb/erq001>
23. H.-D. Seelig, A. Hoehn, L.S. Stodieck, D.M. Klaus, W.W. Adams et al., Plant water parameters and the remote sensing R1300/R1450 leaf water index: controlled condition dynamics during the development of water deficit stress. Irrig. Sci. **27**, 357–365 (2009). <https://doi.org/10.1007/s00271-009-0152-5>
24. T.U. Rehman, D. Ma, L. Wang, , L. Zhang, J. Jin, Predictive spectral analysis using an end-to-end deep model from hyperspectral images for high-throughput plant phenotyping. Comp. Electron. Agriculture **177**, 105713 (2020). <https://doi.org/10.1016/j.compag.2020.105713>
25. B. Zhang, Z. Dong, X. Sui, J. Gao, L. Feng, Detection of water content in tomato stems by electrical impedance spectroscopy: Preliminary study. Comp. Electron. Agriculture **219**, 108755 (2024). <https://doi.org/10.1016/j.compag.2024.108755>
26. D. Sancho-Knapik, J. Gismero, A. Asensio, J.J. Peguero-Pina, V. Fernández et al., Microwave L-band (1730MHz) accurately estimates the relative water content in poplar leaves. A comparison with a near infrared water index (R1300/R1450). Agricul. Forest Meteorology **151**, 827–832 (2011). <https://doi.org/10.1016/j.agrformet.2011.01.016>
27. E.C. Oerke, U. Steiner, H.W. Dehne, M. Lindenthal, Thermal imaging of cucumber leaves affected by downy mildew and environmental conditions. J. Exp. Bot. **57**, 2121–2132 (2006). <https://doi.org/10.1093/jxb/erj170>
28. D. Jamaludin, S. Abd Aziz, D. Ahmad, H.Z.E. Jaafar, Impedance analysis of Labisia pumila plant water status. Inform. Proc. Agriculture **2**, 161–168 (2015). <https://doi.org/10.1016/j.inpa.2015.07.004>
29. L. Milić, M. Radovanović, M. Simić, S. Qureshi, Đ. Micić et al., Development of E-tattoo sensors for monitoring of plants hydration level. 2024 IEEE 22nd Mediterranean Electrotechnical Conference (MELECON). June 25-27, 2024, Porto, Portugal. IEEE, (2024)., 36–40.
30. C. Qu, L. Cao, M. Li, X. Wang, Z. He Liquid metal-based plant electronic tattoos for *in situ* monitoring of plant physiology. Sci. China Technol. Sci. **66**, 1617–1628 (2023). <https://doi.org/10.1007/s11431-023-2431-4>
31. T. Iwashita, H. Katayanagi, N. Miki, Needle-type in situ water content sensor with polyethersulfone polymer membrane. Sens. Actuat. B: Chem. **154**, 41-45 (2011). <https://doi.org/10.1016/j.snb.2009.11.019>
32. N.C. Turner, Measurement of plant water status by the pressure chamber technique. Irrig. Sci. **9**, 289–308 (1988). <https://doi.org/10.1007/bf00296704>
33. A. Kumar, S.S. Han, PVA-based hydrogels for tissue engineering: a review. Int. J. Polym. Mater. Polym. Biomater. **66**, 159-182 (2017). <https://doi.org/10.1080/00914037.2016.1190930>
34. J.W. Fluhr, R. Darlenski, C. Surber, Glycerol and the skin: Holistic approach to its origin and functions. British J. Dermatology **159**, 2-34 (2008). <https://doi.org/10.1111/j.1365-2133.2008.08643.x>
35. H. Dechiraju, M. Jia, L. Luo, M. Rolandi, Ion-conducting hydrogels and their applications in bioelectronics. Adv. Sustain Syst. **6**, 1-10 (2022). <https://doi.org/10.1002/adsu.202100173>
36. B.B. Mandal, S. Kapoor, S.C. Kundu, Silk fibroin/polyacrylamide semi-interpenetrating network hydrogels for controlled drug release. Biomaterials **30**, 2826–2836 (2009). <https://doi.org/10.1016/j.biomaterials.2009.01.040>
